# Sustainable electrochemical synthesis of aliphatic nitro-*NNO*-azoxy compounds employing ammonium dinitramide and their in vitro evaluation as potential nitric oxide donors and fungicides

**DOI:** 10.3762/bjoc.21.211

**Published:** 2025-12-29

**Authors:** Alexander S Budnikov, Nikita E Leonov, Michael S Klenov, Andrey A Kulikov, Igor B Krylov, Timofey A Kudryashev, Aleksandr M Churakov, Alexander O Terent’ev, Vladimir A Tartakovsky

**Affiliations:** 1 N.D. Zelinsky Institute of Organic Chemistry of the Russian Academy of Sciences, 47 Leninsky prosp., 119991 Moscow, Russian Federation

**Keywords:** ammonium dinitramide, antifungal agents, azoxy compounds, electrosynthesis, NO donors

## Abstract

An atom- and step-economical electrochemical method for the synthesis of aliphatic nitro-*NNO*-azoxy compounds from the corresponding nitroso compounds was developed employing ammonium dinitramide, a prospective green oxidant for aerospace propulsion applications, as both electrolyte and source of a =NNO_2_ group. The developed method is green, practical, and scalable due to constant current electrolysis in an undivided cell at high current densities. Synthesized products demonstrated pronounced NO-donor activity and fungicidal activity against phytopathogenic fungi.

## Introduction

Organic compounds containing N–N and N–O bonds are ubiquitous in diverse fields, including pharmaceuticals, agrochemicals, natural products [1–4], as well as in applications in organic synthesis as precursors for free radicals in selective transformations [5–11] and polymerization initiators [12], energetic materials [13–15], NO donors [16–17], and organic light-emitting diodes (OLEDs) [18]. Despite the wide diversity of their applications, structures, and redox properties, effective synthetic strategies for the construction of N–N and N–O systems remain markedly underdeveloped. Unlike traditional methods for constructing C–C and C–Het bonds, the number of methods for selective N–N [1,5,19–29] and N–O [30–35] coupling is very limited. In most existing approaches, the N–N and N–O moieties are derived from hydrazine or hydroxylamine synthons, respectively. The development of novel synthetic methodologies based on non-traditional retrosynthetic disconnections offers a potential pathway to overcome existing constraints in functional group compatibility, efficiency, and reagent scope. In this regard, the direct formation of nitrogen-nitrogen or nitrogen-oxygen bonds presents a significant challenge due to the variety of possible side processes and the low thermodynamic driving force for N–N and N–O bond formation. However, it also represents a highly efficient and valuable strategy due to the synthetic availability of starting materials. Therefore, the development of new methods for direct selective N–N and N–O coupling remains an important goal in synthetic chemistry.

To date, electroorganic synthesis has become a powerful and reliable strategy for the functionalization of organic compounds under green and mild reaction conditions [36–39]. The control of selectivity through the variation of parameters such as current, voltage, electrolyte, electrodes, and the type of electrochemical cell distinguishes electroorganic synthesis from traditional organic chemistry methods. Particular attention is paid to the generation of radical intermediates via the oxidation or reduction of radical precursors [40–49]. In this regard, the anodic oxidation of nitrogen compounds leading to the formation of *N*-centered radicals has proven to be a convenient approach [15,42,47–48] to the formation of C–N [50–51] and N–Het [52–53] bonds. However, the electrochemical construction of N–N and N=N bonds remains very limited. The vast majority of developed approaches focus mainly on intramolecular radical cyclizations [42,54–63], while intermolecular [1,64–68] N–N coupling remains a poorly studied area.

In addition to the preparation of azo compounds [69–70], intermolecular N–N coupling reactions are of particular interest for the regioselective synthesis of azoxy compounds [5,71–72]. Unique and understudied representatives of the latter are nitro-*NNO*-azoxy compounds [R–N(O)=N–NO_2_], first synthesized at ZIOC RAS [73–75]. Nowadays, aromatic and heterocyclic compounds of this class have been studied in most detail and attract continuing attention as perspective high-energy materials [76–86] and physiologically active substances [87]. At the same time, aliphatic compounds containing the nitro-*NNO*-azoxy group are practically unknown, with the exception of a few representatives [88].

**Scheme 1:**
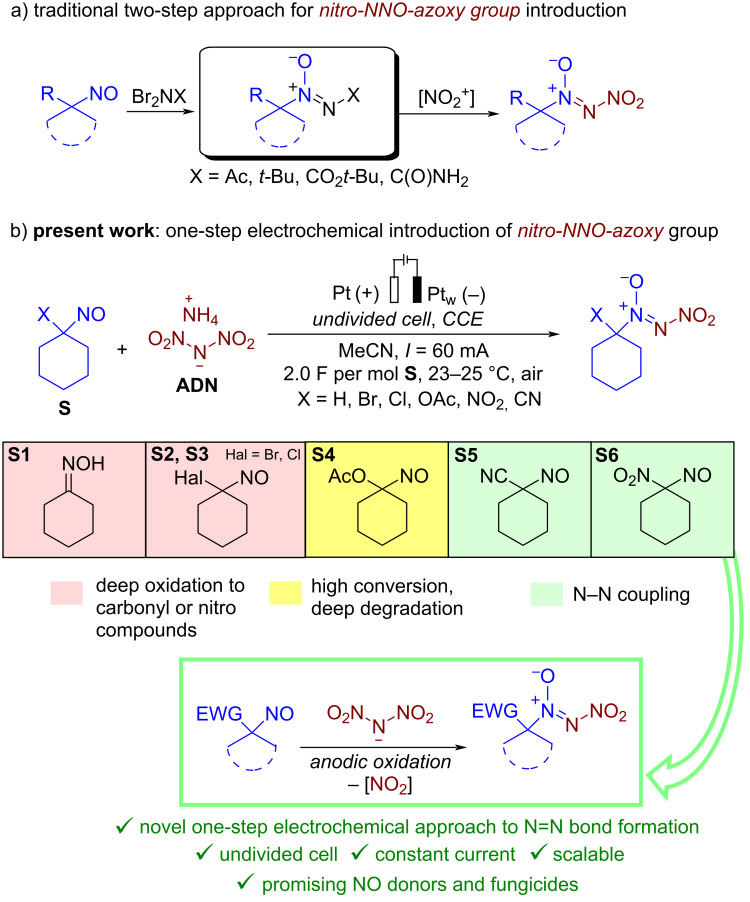
Current synthetic approaches to aliphatic nitro-*NNO*-azoxy compounds and the summary of the present work achievements.

The most general two-step approach to the synthesis of nitro-*NNO*-azoxy compounds is shown in Scheme 1a. The first step includes the reaction of nitroso compounds R–N=O with *N*,*N*-dibromoamines Br_2_NX (X = Ac [73], *t-*Bu [74], CO_2_*t-*Bu [74], C(O)NH_2_ [74]) leading to azoxy compounds containing a leaving group X. The second step is substitutive nitration of the latter with nitronium salts (NO_2_BF_4_, (NO_2_)_2_SiF_6_) or N_2_O_5_). The main limitations of this approach are the use of hazardous and expensive nitrating reagents and its inapplicability for preparation of nitro-*NNO*-azoxy compounds containing substituents that are labile towards electrophiles. Thus, there is a need for new, more efficient methods for the synthesis of nitro-*NNO*-azoxy derivatives that do not have the disadvantages of the known approach and allow the use of a wide range of substrates, including various aliphatic ones.

Recently, our group developed a one-step electrochemical approach to the synthesis of nitro-*NNO*-azoxy arenes employing ammonium dinitramide (ADN) [87]. The achieved use of ADN, known as prospective chlorine-free green oxidant for aerospace propulsion applications [89–91], as both a reagent and an electrolyte is very important, as it overcomes one of the major obstacles to the sustainability and scalability of organic electrosynthesis: the need for a supporting electrolyte in addition to reactants. The synthesized nitro-*NNO*-azoxy arenes were discovered as a novel class of fungicides, with in vitro activity against a broad range of phytopathogenic fungi that is comparable or superior to that of commercial fungicides. In the present study, we sought to expand the developed nitro-*NNO*-azoxylation strategy to aliphatic nitroso compounds. However, our numerous attempts involving various nitroso compounds or oximes (X = H) with ADN were unsuccessful, as the NO group or oxime group did not participate in the desired N=N coupling (Scheme 1, substrates **S1**–**S4**). These substrates underwent either deep oxidation or degradation. Ultimately, in the reaction of 1-nitrosocyclohexane-1-carbonitrile (**S5**) and 1-nitro-1-nitrosocyclohexane (**S6**) we observed products of N=N coupling with ammonium dinitramide. Presumably, the presence of a strong electron-withdrawing group prevents further oxidation of the NO group prior to the oxidation of ADN. Herein, we report a green and sustainable electrochemical coupling of aliphatic nitroso compounds with ammonium dinitramide, leading to efficient formation of the nitro-*NNO*-azoxy group. The developed strategy involves performing the electrosynthesis in an undivided cell under high current density with ADN employed as both a reagent and an electrolyte. The synthesized aliphatic nitro-*NNO*-azoxy compounds were identified as promising NO-donors and were found to exhibit pronounced fungicidal activity against a broad spectrum of phytopathogenic fungi.

## Results and Discussion

The conditions of the electrochemical coupling of aliphatic nitroso compounds with ammonium dinitramide (ADN) leading to the construction of a nitro-*NNO*-azoxy fragment were optimized on the model reaction of 2-nitro-2-nitrosopropane (**1a**) and ADN as coupling partners (Table 1). The influence of material of electrodes, the amount of electricity, current density, supporting electrolyte, solvent nature, and atmosphere are summarized in Table 1.

**Table 1:** Optimization of the reaction conditions.^a^

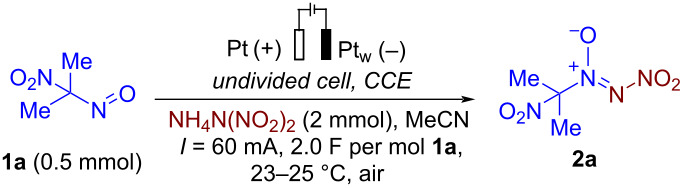		

Entry	Variations from standard reaction conditions	Yield^b^ **2a**, %

**1**	**none**	**77 (75)**
2	SS/Ni/C_F_/C/GC as anode (+)	<5/<5/56/29/68
3	Pt/SS/Ni/C_F_/C/GC as cathode (−)	63/54/70/27/53/67
4	1/1.5/3 F per mole of **1a**, *I* = 60 mA, (+) Pt/Pt_w_ (−)	48/68/76
5	*I* = 15/30/120/240 mA, (+) Pt/Pt_w_ (−)	72/77/62/32
6	ADN (0.5 mmol), *n-*Bu_4_NBF_4_ (0.5 mmol)	28
7	KDN (2 mmol), *n-*Bu_4_NBF_4_ (0.5 mmol)	26
8	1/1.5/2.5 mmol ADN	37/60/69
9	acetone/DMF/MeOH/HFIP as solvent	<5/n.d./<5/<5
10	Ar atmosphere	65
11	without electricity	n.d.

^a^Standard reaction conditions: 2-nitro-2-nitrosopropane (**1a**, 60 mg, 0.5 mmol), ADN (62–310 mg, 0.5–2.5 mmol), electrolyte or additive (0–288 mg), solvent (10 mL), undivided cell, constant current electrolysis (CCE) with *I =* 15–240 mA, *F* = 1–3 F/mol **1a** (reaction time 7–107 min), air atmosphere. C – graphite plate, GC – glassy carbon plate, C_F_ – carbon felt, Pt – platinum plate, Pt_w_ – platinum wire, SS – stainless steel plate, Ni – nickel plate. ^b^The yield was determined by ^1^H NMR using 1,1,2,2-tetrachloroethane as an internal standard; the isolated yields are given in parentheses. n.d. – not detected. The bold text of entry 1 indicates conditions as optimal.

After extensive optimization, we found that a 77% yield (75% isolated yield) of **2a** was achieved by performing the model reaction under CCE (constant current electrolysis) conditions in an undivided electrochemical cell, using a platinum plate as the anode and a platinum wire as the cathode, with 4 equiv of ADN as both the reagent and the supporting electrolyte (Table 1, entry 1). Entries 2 and 3 show that the best results were obtained with a platinum plate anode and a platinum wire cathode. The evaluation of the charge passed revealed that the optimal amount was 2 F per mole **1a** (Table 1, entry 4). Increasing the current density resulted in a drop in the yield to 32%, while reducing the current density had a negligible effect on the yield of **2a** (Table 1, entry 5). Employing 1 equivalent of ADN or potassium dinitramide (KDN) along with 1 equivalent of *n*-Bu_4_NBF_4_ as the supporting electrolyte proved ineffective: the yield of **2a** did not exceed 28% (Table 1, entries 6 and 7). Screening of the amount of ADN revealed that 2 mmol per 0.5 mmol of **1a** was optimal (Table 1, entry 8). MeCN proved to be the best solvent for the present electrochemical coupling, while performing the reaction in other polar protic and aprotic solvents resulted in only trace amounts of **2a** (Table 1, entry 9). Entry 10 shows that no significant change in the yield of **2a** was observed when conducting the electrosynthesis in an inert atmosphere. Finally, no reaction was observed in the absence of electrical current (Table 1, entry 11).

**Scheme 2:**
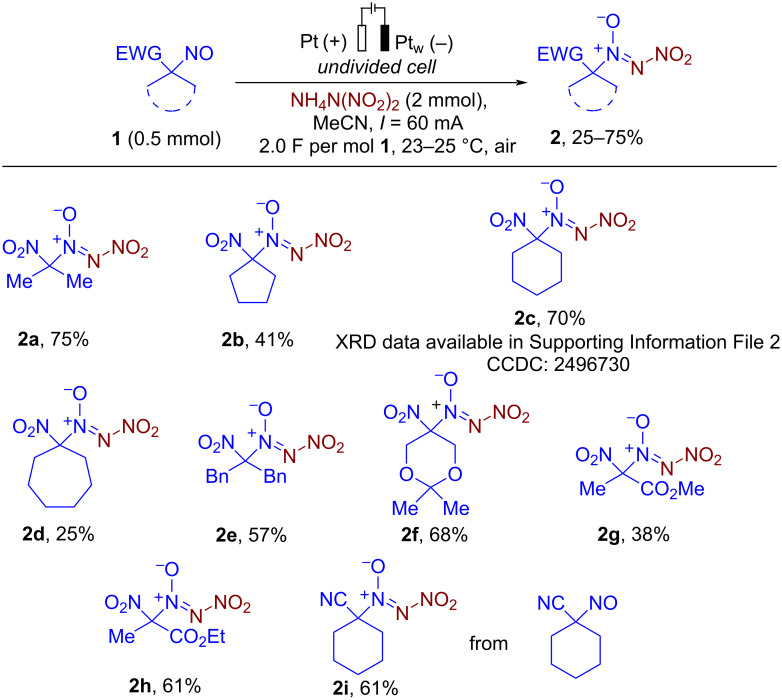
Scope of the discovered electrochemical nitro-*NNO*-azoxylation of nitrosoalkanes containing electron-withdrawing groups.

Under optimized reaction conditions (Table 1, entry 1), the scope of the electrochemical nitro-*NNO*-azoxylation protocol was tested (Scheme 2).

The reaction proceeds in moderate to good yields for cyclic aliphatic nitroso compounds **1b–d**, affording nitro-*NNO*-azoxy compounds **2b–d**. The structure of **2c** was confirmed by single-crystal X-ray analysis. The benzyl moiety was also tolerated, and the corresponding product **2e** was obtained in 57% yield. 2,2-Dimethyl-5-nitro-5-nitroso-1,3-dioxane (**1f**) was successfully employed under the reaction conditions, leading to the formation of product **2f** in 68% yield without cleavage of the acetonide protecting group. Note that compound **2f** is not accessible using the known approach (see Scheme 1a), because the dioxane ring is opened during the nitration step. Surprisingly, nitro-*NNO*-azoxylation products **2g** and **2h** were obtained from methyl and ethyl 2-nitro-2-nitrosopropanoates (**1g** and **1h**), which contain an additional electron-withdrawing ester group in geminal position. Finally, 1-nitrosocyclohexane-1-carbonitrile (**1i**) was also tolerated under the reaction conditions, affording product **2i** in 61% yield.

**Scheme 3:**
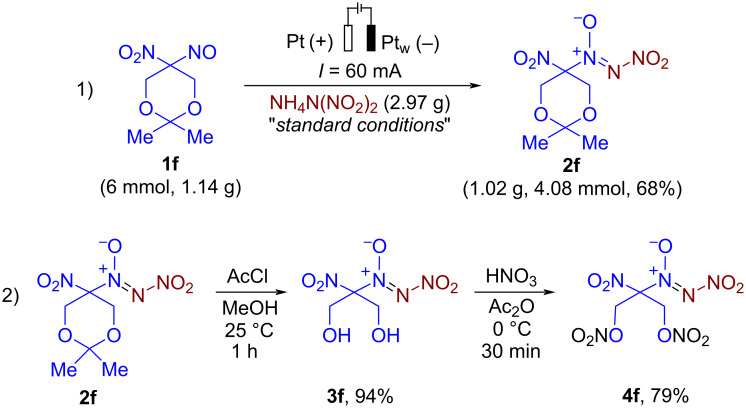
Synthetic utility and derivatization of synthesized coupling product **2f**.

The robustness of the developed protocol was further demonstrated by performing the model reaction on a 6 mmol scale of **1f** (Scheme 3, reaction 1). The corresponding product **2f** was obtained without any erosion of the yield (1.02 g, 4.08 mmol, 68%). The acidic cleavage of the acetonide group in **2f** with AcCl in MeOH afforded diol **3f** in a 94% yield. Subsequent nitration of the obtained diol **3f** with a HNO_3_/Ac_2_O mixture afforded dinitrate **4f** in 79% (74% over two stages, Scheme 3, reaction 2). Due to the presence of two nitrate groups, compound **4f** may be of interest as a potential NO donor [92] and a precursor of new nitro-*NNO*-azoxy compounds via nucleophilic substitution of –ONO_2_ groups.

**Figure 1:**
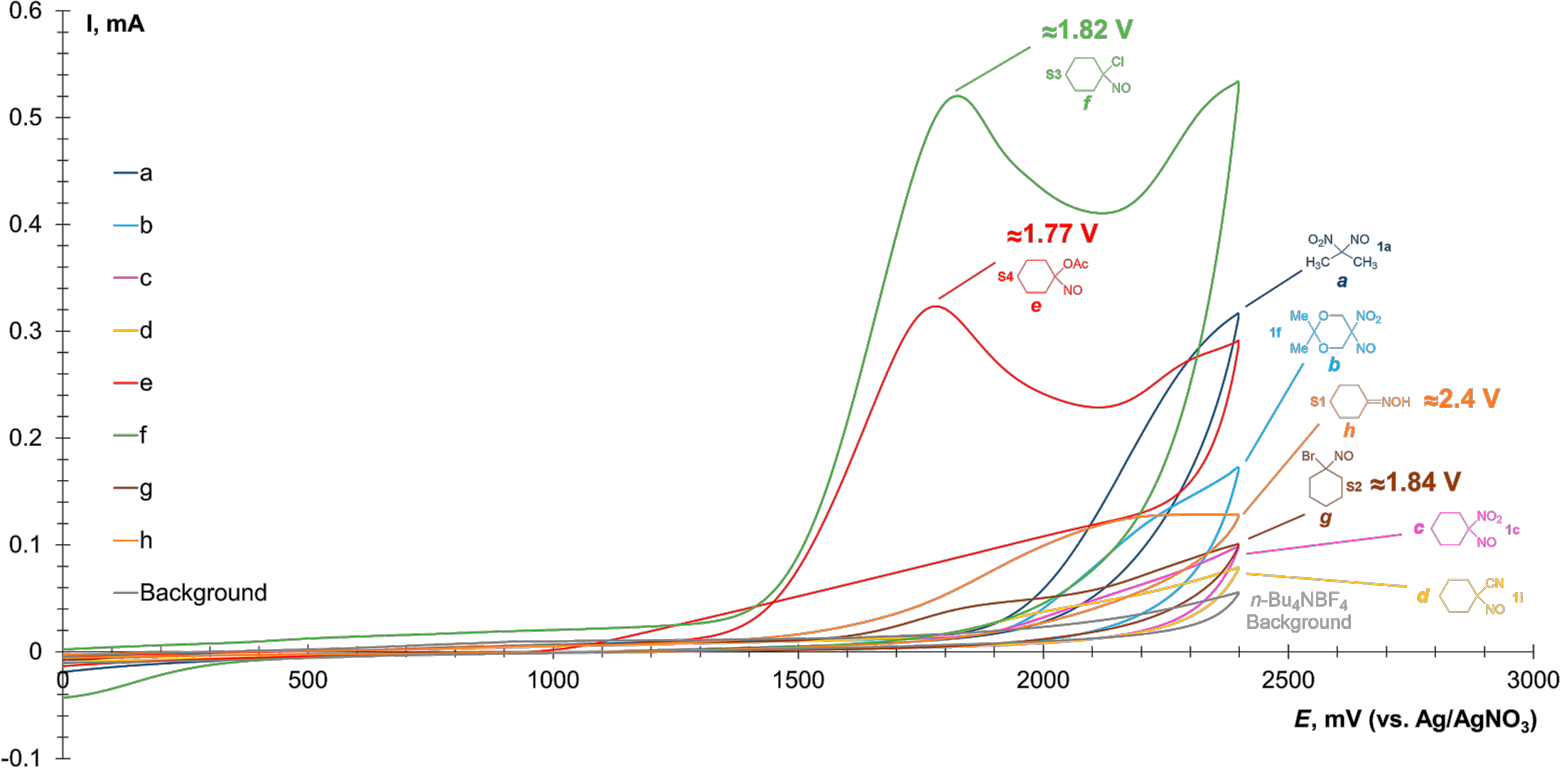
CV-curves of 0.01 M solutions of a) **1a** (blue), b) **1f** (azure), c) **1c** (pink), d) **1i** (yellow), e) **S4** (red), f) **S3** (green), g) **S2** (brown), and h) **S1** (orange) in 0.1 M *n*-Bu_4_NBF_4_ solution in MeCN on a working glassy-carbon electrode (*d* = 3 mm) under a scan rate of 0.1 V/s at 298 K.

To clarify the reaction mechanism, cyclic voltammetry (CV) studies were conducted. CV curves of cyclohexanone oxime (**S1**), 1-bromo-1-nitrosocyclohexane (**S2**), 1-chloro-1-nitrosocyclohexane (**S3**), 1-nitrosocyclohexyl acetate (**S4**), 1-nitro-1-nitrosocyclohexane (**1c**), 1-nitrosocyclohexane-1-carbonitrile (**1i**), 2-nitro-2-nitrosopropane (**1a**), and 2,2-dimethyl-5-nitro-5-nitroso-1,3-dioxane (**1f**) were recorded on a working glassy-carbon electrode in MeCN solution (Figure 1). Tetrabutylammonium tetrafluoroborate was chosen as the supporting electrolyte.

As shown by the voltammograms of 0.01 M solutions of 1-nitro-1-nitroso compounds **1a**, **1f**, **1c**, and **1i** in MeCN, these substances do not undergo noticeable oxidation up to +2.4 V. On the other hand, 1-chloro-1-nitrosocyclohexane (**S3**, green curve) and 1-nitrosocyclohexyl acetate (**S4**, red curve) are oxidized at +1.82 V and +1.77 V, respectively. 1-Bromo-1-nitrosocyclohexane (**S2**, brown curve) exhibits a broad oxidation peak around +1.84 V, whereas cyclohexanone oxime (**S1**, orange curve) shows no significant oxidation up to +2.4 V. However, its reaction with ADN in the optimized reaction conditions resulted in a complex mixture of oxime oxidation products with full conversion of **S1**. The obtained data indicate that the presence of an electron-withdrawing group (NO_2_, CN) at the carbon atom geminal to the NO group prevents latter from facile oxidation, which, in turn, facilitate the desired nitro-*NNO*-azoxylation.

**Figure 2:**
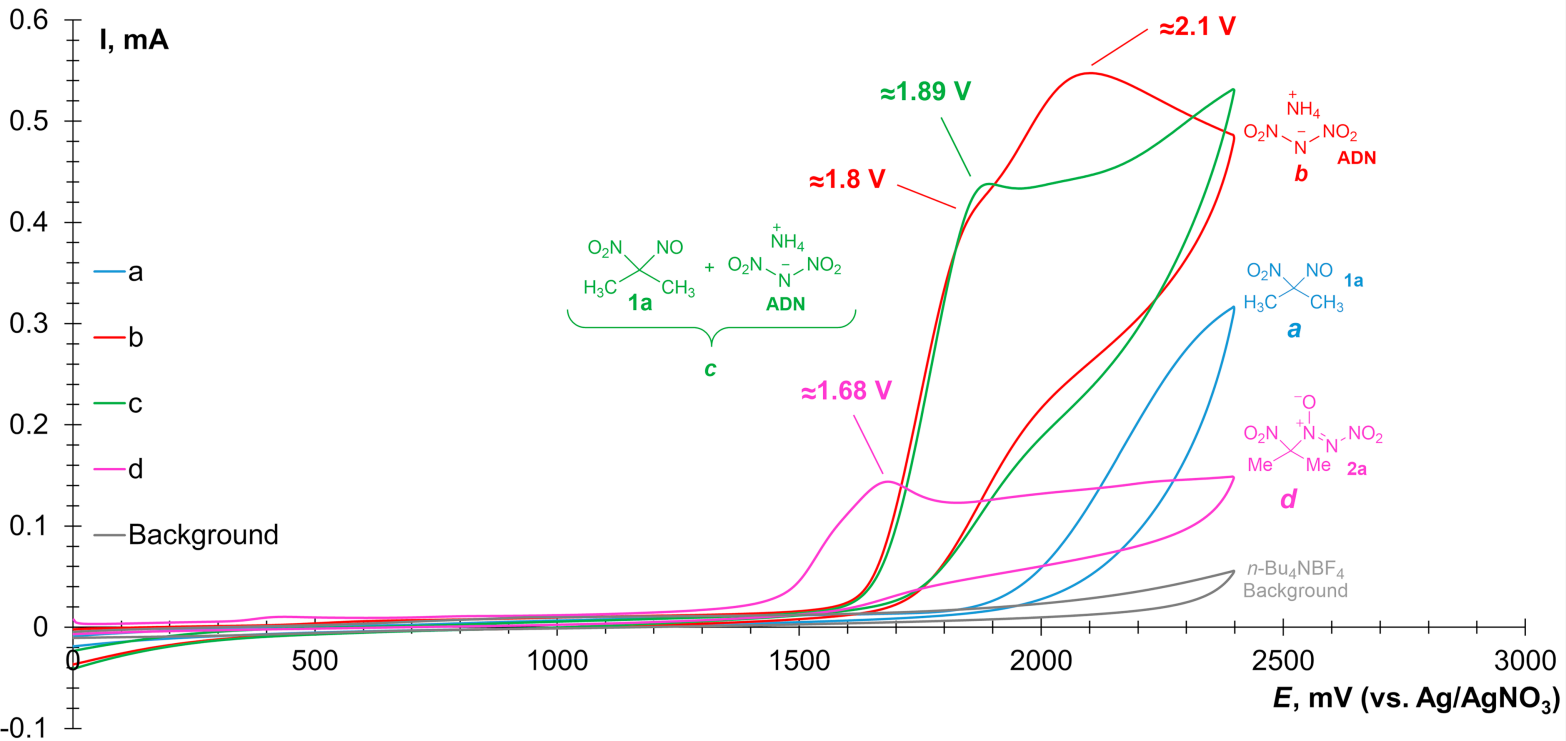
CV-curves of 0.01 M solutions of a) **1a** (blue), b) ADN (red), c) the mixture of **1a** and ADN (green), d) **2a** (pink) in 0.1 M *n*-Bu_4_NBF_4_ solution in MeCN on a working glassy-carbon electrode (*d* = 3 mm) under a scan rate of 0.1 V/s at 298 K.

As previously demonstrated in our study [87], ADN exhibits two irreversible oxidation peaks (red curve) at +1.8 V and +2.1 V, which are higher than or comparable to those of **S2**–**S4** (Figure 2).

Upon the addition of ADN to a solution of **1a** (Figure 2, green curve), a single oxidation peak was observed at +1.89 V. Finally, the reaction product nitro-*NNO*-azoxy propane **2a** exhibits an oxidation peak at +1.68 V (Figure 2, pink curve). Despite the lower oxidation potential of **2a**, we speculate that an excess of ADN suppresses competitive oxidation of the reaction product.

**Scheme 4:**
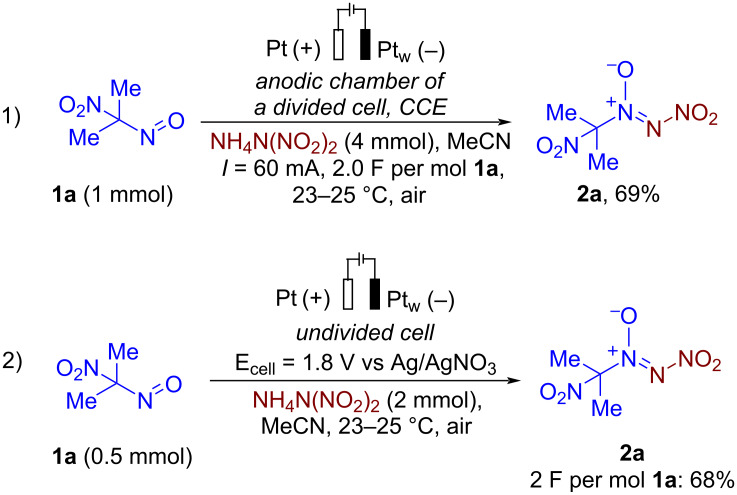
Control experiments.

To gain insight into the reaction mechanism, control experiments were conducted (Scheme 4).

Discovered electrochemical coupling performed in a divided *H*-cell with **1a** and ADN in anodic chamber (Scheme 4, reaction 1) afforded the corresponding nitro-*NNO*-azoxylation product **2a** in a 69% yield. This result suggests that the process proceeds preferentially at anode surface. The studied reaction was also carried out under controlled potential conditions (Scheme 4, reaction 2) at E ≈ 1.8 V for 2.0 F per mol **1a**. In this case, the formation of **2a** was observed in a 68% yield.

**Figure 3:**
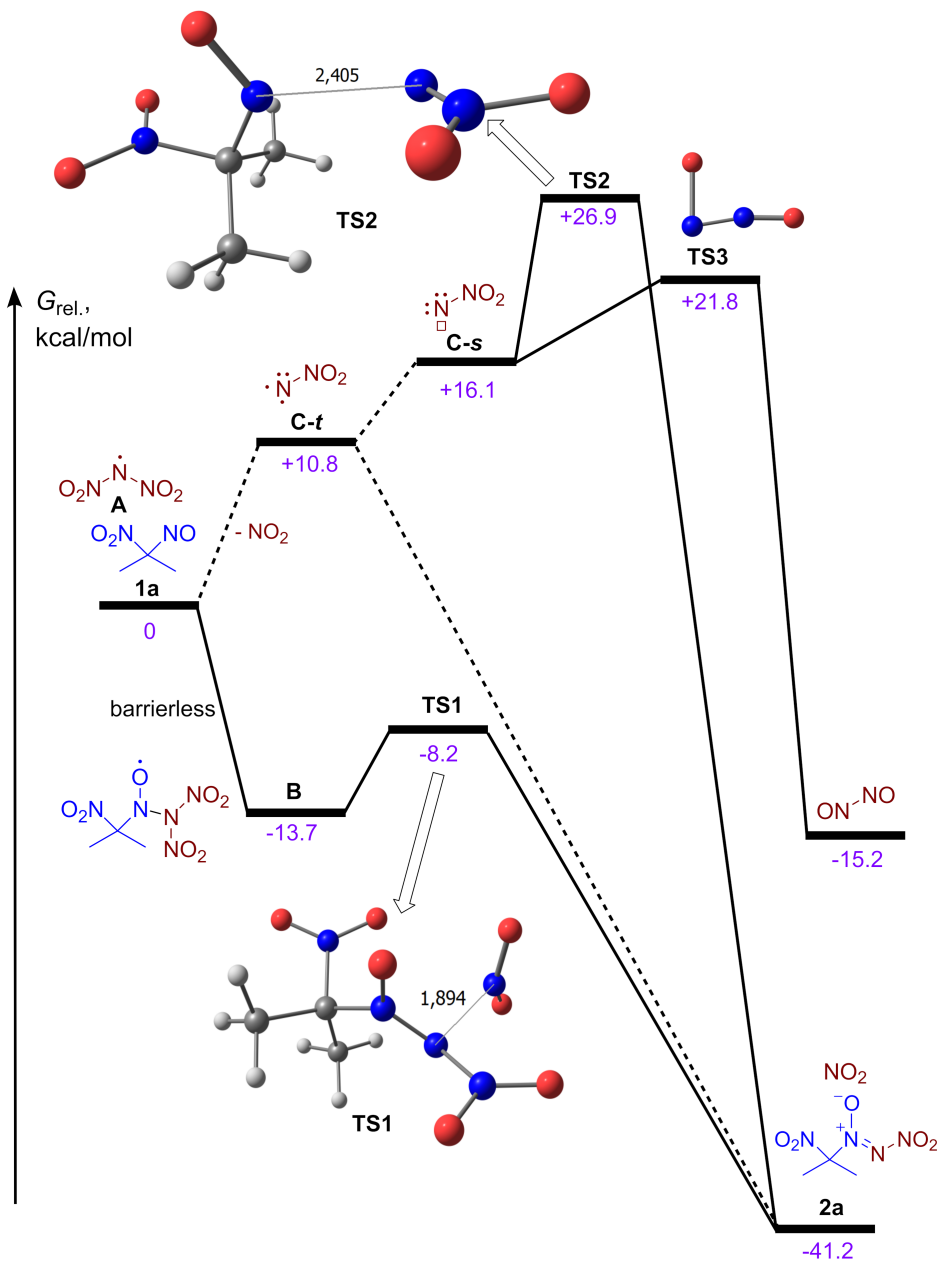
Free energy diagram of possible interaction pathways between **1a** and dinitramide-derived radical **A** according to ωB97X-3c [94]/CPCM(MeCN) level of theory. Free energies Δ*G* and activation free energies Δ*G*^≠^ are given in kcal/mol. Dashed lines correspond to spin-state-changing transformations that may have additional energy barriers (not estimated).

**Scheme 5:**
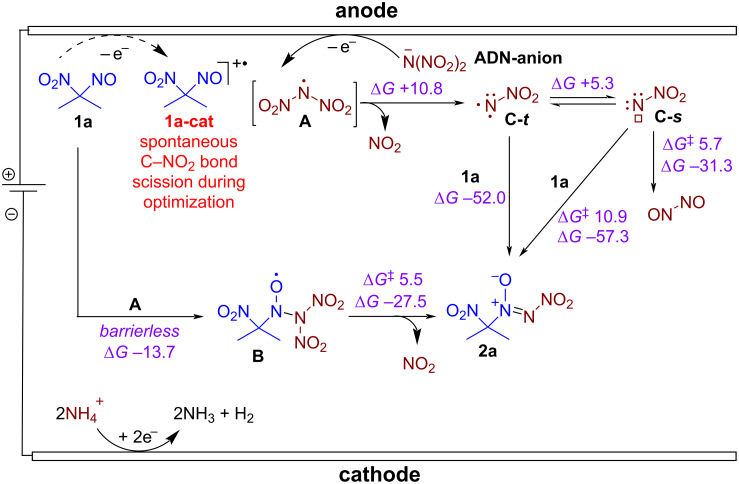
Proposed mechanism for electrochemical nitro-*NNO*-azoxylation of 1-nitro-1-nitroso compounds **1**. Free energies Δ*G* and activation free energies Δ*G*^≠^ are given in kcal/mol.

To gain insight into the reaction mechanism of the discovered nitro-*NNO*-azoxylation process quantum chemical calculations were performed [93] on ωB97X-3c [94] /CPCM(MeCN) level of theory (Figure 3). Based on cyclic voltammetry data, control experiments, and our previous study, the mechanism of the electrochemical synthesis of nitro-*NNO*-azoxy compounds **2** was proposed (Figure 3, Scheme 5).

The reaction starts from direct anodic oxidation of dinitramide anion with the formation of *N*-centered radical **A**. Monitoring of the reaction potential during electrolysis under standard conditions using a reference electrode revealed that the measured potential ranged from 2.2 V (at the start of the reaction) to 2.4 V (at the end of the reaction), which is sufficient to oxidize ADN. According to the performed calculations, competitive oxidation of **1a** does not occur, which is confirmed by the obtained CV-data (see Figure 1 and Figure 2). According to DFT calculations, the reaction of *N*-radical **A** with 2-nitro-2-nitrosopropane (**1a**) is expected to be barrierless with the formation of *N*-oxyl radical **B**. The radical **B** undergoes very low-barrier (Δ*G*^≠^ 5.5 kcal/mol) fragmentation with the release of NO_2_ molecule and formation of the final product **2a**. An alternative pathway for the formation of **2a** is a direct NO_2_ extrusion from the *N*-centered radical **A** with the formation of the nitro-nitrene **C** [87]. This nitrene can exist in a singlet state (**C-*s***) or triplet state (**C-*t***), the latter being 5.3 kcal/mol more stable. The formation of more stable **C-*t*** from **A** is about 10.8 kcal/mol uphill. The barrier of **C-*t*** addition to **A** was not estimated, as this step requires the change in spin-state. However, unexpectedly we found that addition of higher energy singlet nitrene **C-*s*** to **A** is not barrierless (Δ*G*^≠^ 10.9 kcal/mol). Moreover, according to our calculations **C-*s*** can easily (Δ*G*^≠^ 5.7 kcal/mol) undergo unimolecular rearrangement to the dimer of nitric oxide (N_2_O_2_). These results allow us to think that the reaction pathway via nitrene **C-*s*** is less plausible compared to the interaction of radical **A** with **1a**.

### Aliphatic nitro-*NNO*-azoxy compounds as potential NO donors and their in vitro fungicidal activity

At the second part of our study, the synthesized nitro-*NNO*-azoxy compounds were tested for fungicidal activity against phytopathogenic fungi and for NO-release activity. Earlier we have demonstrated [87] that nitro-*NNO*-azoxy arenes were discovered as a novel class of fungicides, with in vitro activity against a broad range of phytopathogenic fungi that cause a global threat to crop production and public health [95–99]. Herein, the synthesized nitro-*NNO*-azoxy alkanes were screened for antifungal activity against phytopathogenic fungi representing several taxonomic classes: *Venturia inaequalis* (*V.i.*, ascomycete causing the apple scab), *Rhizoctonia solani* (*R.s.*, basidiomycete causing potato black scurf), *Fusarium oxysporum* (*F.o.*, ascomycete affecting the vascular system of plants and inducing wilting), *Fusarium moniliforme* (*F.m.,* ascomycete known also as *Fusarium verticillioides* in modern literature, important pathogen of maize), *Bipolaris sorokiniana* (*B.s.*, ascomycete causing root rot and spot blotch), and *Sclerotinia sclerotiorum* (*S.s.*, ascomycete affecting sunflower, potato, and other cultures). The degree of mycelium growth inhibition on potato-sucrose agar amended with the studied compounds (30 mg/L) was used as the criterion for evaluating fungicidal activity. The commercially available fungicide triadimefon was used as a reference compound.

As can be seen from Table 2, among the tested compounds **2a–I**, the highest fungicidal activity was demonstrated by **2i**, which contains a nitrile substituent. It was the most active against five of six fungi, except *S.s*. The model compound 2-nitro-2-nitro-*NNO*-azoxy propane **2a** showed superior activity compared to that of triadimefon against *R.s*. and *F.m*. The other synthesized compounds (**2b–h** and **3f**) do not show significant fungicidal activity, except for dinitrate **4f**, which was the most active against *R.s.* and *B.s*. The results from Table 2 indicate that the synthesized compounds **2a–i** represent a potentially new class of fungicides with unforeseen mode of action, which makes them a promising starting point for the development of novel crop protection agents.

**Table 2:** In vitro fungicidal activity of the synthesized nitro-*NNO*-azoxy compounds **2***^a^*.

Entry	Compound	Mycelium growth inhibition (%)					

		*V. i.*	*R. s.*	*F. o.*	*F. m.*	*B. s.*	*S. s.*

1	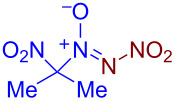 **2a**	6	**89**	33	**94**	11	21
2	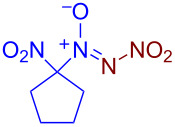 **2b**	7	24	4	11	15	8
3	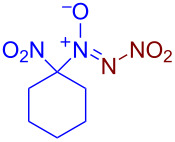 **2c**	4	11	11	**77**	11	14
4	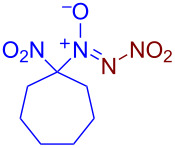 **2d**	0	29	8	7	22	8
5	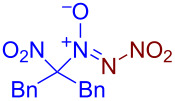 **2e**	13	**57**	16	32	21	17
6	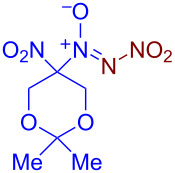 **2f**	6	2	0	5	3	10
7	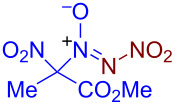 **2g**	0	22	5	6	15	15
8	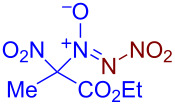 **2h**	0	34	5	11	14	11
9	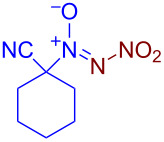 **2i**	**100**	**100**	**91**	**100**	**64**	46
10	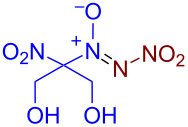 **3f**	0	13	6	0	0	4
11	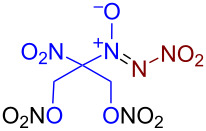 **4f**	25	**52**	40	81	**48**	22
12	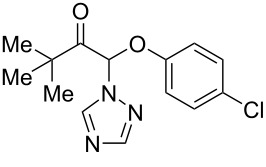 (±)-triadimefon (reference compound)	41	43	77	87	44	61

^a^The data on fungicidal activity exceeding the standard (triadimefon) are printed in bold. Concentration of compounds in nutrient medium 30 mg·L^−1^.

**Figure 4:**
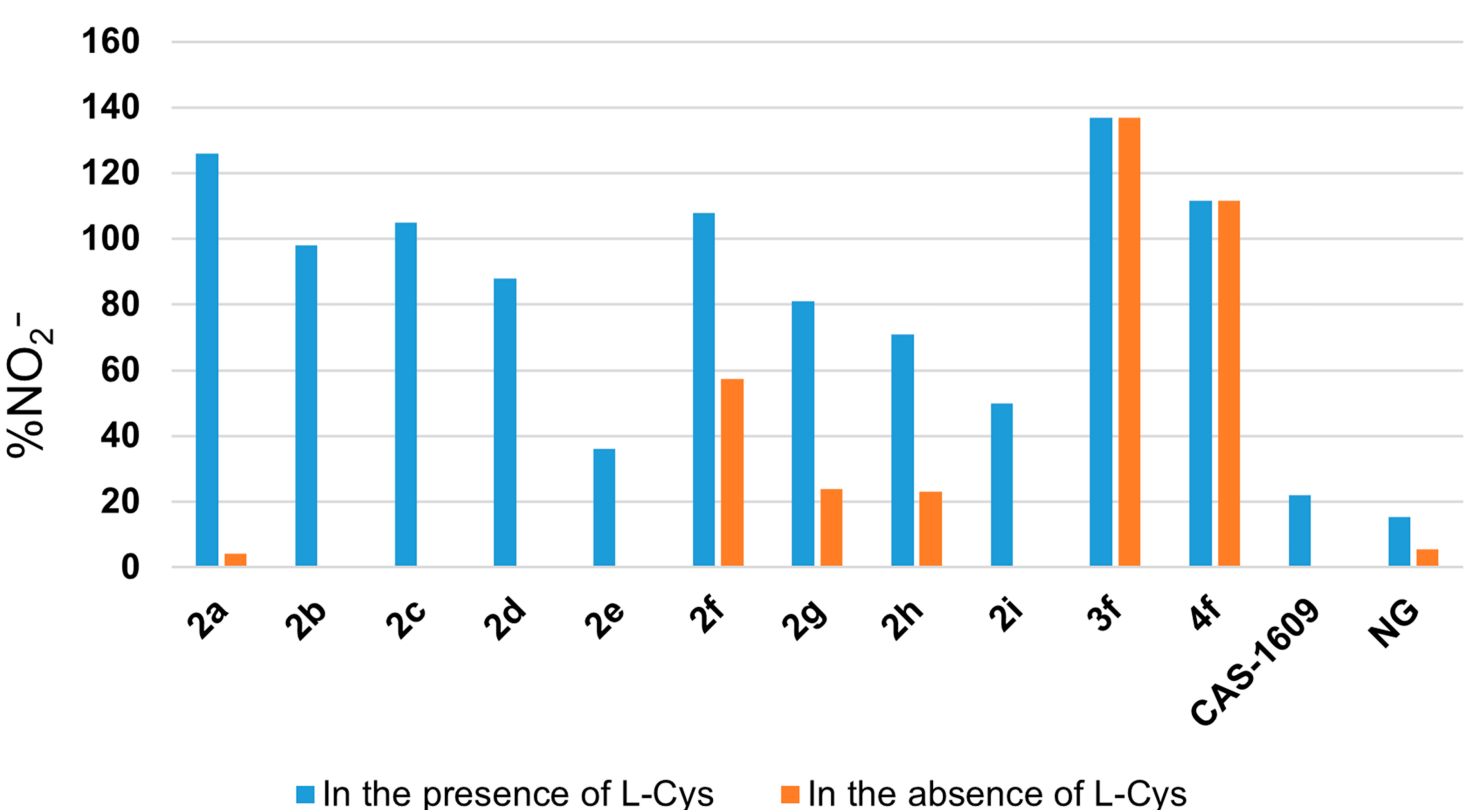
Assessment of the NO release from compounds **2a–i**, **3f**, and **4f**.

Finally, we investigated an ability to release NO for the synthesized aliphatic nitro-*NNO*-azoxy compounds (Figure 4).

Well-known NO-donor compounds 3-carbamoyl-4-(hydroxymethyl)furoxan (CAS-1609) [100–101] and nitroglycerin (NG) [102] were used as reference agents. The NO_2_^−^ assumed to be formed as a result of NO oxidation was quantified by Griess assay, which serves as a generally accepted tool to estimate the NO release. The amounts of NO_2_^−^ generated from nitro-*NNO*-azoxy compounds at pH 7.4 and 37 °C (physiological conditions) for 1 h were estimated spectrophotometrically via Griess reaction. According to the Griess test results, all synthesized nitro-*NNO*-azoxy compounds released high fluxes of NO (36–137%) exceeding those of CAS-1609 (22.1%) and NG (15.3%). Interestingly, azoxy derivatives **2a–e**,**i** showed NO-donor properties only in the presence of ʟ-cysteine proposing the thiol-dependent mechanism of NO release. At the same time, compounds **2f–h** demonstrated moderate NO-donor properties in the absence of ʟ-cysteine, which can be associated with a more complex mechanism of NO release for these substances. Importantly, azoxy species **3f** and **4f** showed no difference in NO-donor properties irrespective of the presence of ʟ-cysteine. It should be noted that despite the fact that the Griess test is widely used for the estimation of NO-donor ability for various N,O-containing compound classes, this method is indirect and deeper studies of the NO-release by nitro-*NNO*-azoxy compounds are to be performed in the future. Overall, the synthesized nitro-*NNO*-azoxy compounds are capable of NO release in a wide range of concentrations which is useful for various biomedical insights [16,103–105].

## Conclusion

In summary, a green, safe, atom- and step-efficient electrochemical protocol for the synthesis of nitro-*NNO*-azoxy compounds from nitrosoalkanes with electron-withdrawing groups using ammonium dinitramide (ADN) was developed. One of the key features of the sustainable design of discovered process is the usage of ADN as both an electrolyte and a reagent. The proposed approach is operationally simple and scalable and can be performed in an undivided cell under high current densities without a loss of selectivity. The use of ammonium dinitramide as a source of the [=N–NO_2_] group and an electrolyte eliminates the need for hazardous nitronium salts and allows the synthesis of nitro-*NNO*-azoxy compounds in a single step. The synthesized products demonstrated marked in vitro NO-donor ability and fungicidal activity against a broad range of phytopathogenic fungi.

## Experimental

Although we have encountered no difficulties during preparation and handling of compounds described and used in this paper, they are explosive energetic materials which are sensitive to impact and friction. Mechanical actions of these energetic materials, involving scratching or scraping, must be avoided. Experimental procedures involving ammonium dinitramide impose a potential risk of detonation due to its photosensitivity and hygroscopic nature. Any manipulations must be carried out by using appropriate standard safety precautions.

^1^H, ^13^C, ^14^N NMR spectra were recorded with Bruker DRX-500 (500.1, 125.8, 36.1 MHz, respectively) and Bruker AV600 (600.1, 150.9, 43.4 MHz, respectively) spectrometers. Chemical shifts are reported in delta (δ) units, parts per million (ppm) downfield from internal TMS (^1^H, ^13^C) or external CH_3_NO_2_ (^14^N negative values of δ_N_ correspond to upfield shifts). The IR spectra were recorded with a Bruker ALPHA-T spectrometer in the range of 400–4000 cm^−1^ (resolution 2 cm^−1^) as pellets with KBr or as a thin layer. High-resolution ESI mass spectra (HRMS) were recorded with a Bruker micrOTOF II instrument. Silica gel 60 Merck (15–40 μm) was used for preparative column and thin-layer chromatography. Silica gel “Silpearl UV 254” was used for preparative column and thin-layer chromatography. Analytical thin-layer chromatography (TLC) was carried out on Merck silica gel 60 F254 and “Silufol” TLC silica gel UV-254 aluminum sheets. All reagents were purchased from Acros and Sigma-Aldrich. Solvents were purified before use, according to standard procedures. All other reagents were used without further purification. Ammonium dinitramide (ADN) [106], 2-nitro-2-nitrosopropane (**1a**) [107], 1-nitro-1-nitrosocyclopentane (**1b**) [108], 1-nitro-1-nitrosocyclohexane (**1c**) [109], 2-nitro-2-nitroso-1,3-diphenylpropane (**1e**) [110], 2,2-dimethyl-5-nitro-5-nitroso-1,3-dioxane (**1f**) [111], methyl 2-nitro-2-nitrosopropanoate (**1g**) [112], ethyl 2-nitro-2-nitrosopropanoate (**1h**) [112], 1-nitrosocyclohexane-1-carbonitrile (**1i**) [113], were prepared according to the reported procedures.

**General procedure for the optimization of the reaction conditions for the synthesis of 2-nitro-2-(nitro-*NNO*-azoxy)propane (2a) from 2-nitro-2-nitrosopropane (1a) (experimental details for**Table 1**):** In a manner analogous to one described in [87], an undivided 10 mL electrochemical cell was equipped with a platinum plate, stainless steel, nickel, graphite, carbon felt, or glassy carbon plate anode (40 × 10 mm) and a platinum plate, platinum wire (*d* = 1 mm, *l* = 113 mm, *n*_coils_ = 9), stainless steel, nickel, graphite or glassy carbon plate cathode (40 × 10 mm), and connected to a DC regulated power supply. Electrodes were completely immersed in the solution given *S* = 4 cm^2^ of working surface. A solution of 2-nitro-2-nitrosopropane (**1a**, 0.5 mmol, 59 mg), and ammonium dinitramide (ADN) (1–5 equiv, 1–5 mmol, 62–310 mg) in 10 mL of MeCN, acetone, DMF, MeOH or HFIP was electrolyzed using constant current conditions (*I* = 15–240 mA) at 23–25 °C under magnetic stirring. After passing 1–3 F∙mol^−1^ of electricity (reaction time 7–161 min), electrodes were washed with CH_2_Cl_2_ (3 × 20 mL). The combined organic phase was washed with H_2_O (20 mL) and brine (20 mL), dried over Na_2_SO_4_, and the solvent was removed in vacuo. The yields of **2a** were determined with the use of ^1^H NMR spectroscopy using 1,1,2,2-tetrachloroethane as an internal standard. NMR and IR spectra of the obtained product **2a** are similar to those reported previously [88].

**Typical procedure for electrochemical synthesis of nitro-*NNO*-azoxy alkanes 2a–i (experimental details for**Scheme 2**):** In a manner analogous to one described in [87], an undivided 10 mL electrochemical cell was equipped with a platinum plate anode (40 × 10 mm) and a platinum wire cathode, and connected to a DC regulated power supply. Electrodes were completely immersed in the solution given *S* = 4.0 cm^2^ of working anode surface. A mixture of 1-nitro-1-nitrosoalkane **1a–i** (0.5 mmol, 59–135 mg), and ADN (4.0 equiv, 2.0 mmol, 248 mg) in MeCN (10 mL) was electrolyzed using constant current conditions (*I* = 60 mA) at 23–25 °C under magnetic stirring. After passing 2.0 F∙mol^−1^ of electricity (reaction time 27 min), electrodes were washed with CH_2_Cl_2_ (3 × 20 mL). The combined organic phase was washed with H_2_O (20 mL) and brine (20 mL), dried over Na_2_SO_4_, and solvent removed in vacuo. Products **2a–i** were isolated by column chromatography on silica gel.

**Procedure for gram scale electrochemical synthesis of 2f (experimental details for**Scheme 3**, reaction 1):** In a manner analogous to one described in [87], an undivided 50 mL electrochemical cell was equipped with a platinum plate anode (30 × 15 mm, S = 4.5 cm^2^) and a platinum wire cathode, and connected to a DC regulated power supply. A solution of 2,2-dimethyl-5-nitro-5-nitroso-1,3-dioxane (**1f**, 6.0 mmol, 1.14 g) and ADN (4.0 equiv, 24.0 mmol, 2.97 g) in MeCN (50 mL) was electrolyzed using constant current conditions (*I* = 60 mA) at 23–25 °C under magnetic stirring. After passing 2.0 F∙mol^–1^ of electricity (reaction time 321 min), electrodes were washed with CH_2_Cl_2_ (3 × 50 mL). The combined organic phase was washed with H_2_O (100 mL) and brine (100 mL), dried over Na_2_SO_4_, and the solvent was removed in vacuo. Product **2f** (1.02 g, 4.8 mmol, 68%) was isolated by column chromatography on silica gel (*R*_f_ = 0.15, petroleum ether/ethyl acetate, 40:1).

**Procedure for deprotection of 2f (experimental details for**Scheme 3**, reaction 2):** Acetyl chloride (4.5 mL, 63.3 mmol) was added dropwise to a stirred solution of 2,2-dimethyl-5-nitro-5-(nitro-*NNO*-azoxy)-1,3-dioxane (**2f**, 1.00 g, 4.0 mmol) in MeOH (10 mL) at 25 °C. After addition, the reaction mixture was stirred at this temperature for 24 h (the completion of reaction was monitored by TLC). Then the solvent was removed in vacuo at 40 °C. Product **3f** (0.79 g, 3.76 mmol, 94%) was isolated by column chromatography on silica gel (*R*_f_ = 0.40, petroleum ether/ethyl acetate, 3:1).

**Procedure for nitration of 3f (experimental details for**Scheme 3**, reaction 2):** 2-Nitro-2-(nitro-*NNO*-azoxy)-1,3-propanediol **3f** (0.63 g, 3.0 mmol) was added in portions to a mixture of acetic anhydride (3.4 mL, 36.0 mmol) and 100% nitric acid (0.6 mL, 13.2 mmol) at 0 °C, and the mixture was stirred at this temperature for 30 min. Then the reaction mixture was poured into ice-water (50 mL) and extracted with CH_2_Cl_2_ (3 × 20 mL). The combined organic phase was washed with water (30 mL), brine (30 mL), dried over Na_2_SO_4_ and solvent removed in vacuo. Product **4f** (0.71 g, 2.37 mmol, 79%) was isolated by column chromatography on silica gel (*R*_f_ = 0.55, petroleum ether/ethyl acetate, 5:1).

**Reaction in divided electrochemical cell (experimental details for**Scheme 4**, reaction 1):** In a manner analogous to one described in [87], a divided *H*-type electrochemical cell (volume of each compartment – 30 mL, divided with DuPont Nafion^®^ N-117 membrane) was equipped with a platinum anode (30 × 15 mm) and a platinum wire cathode, and connected to a DC regulated power supply. Electrodes were completely immersed in the solution given *S* = 4.5 cm^2^ of working surface. A solution of 2-nitro-2-nitrosopropane (**1a**, 1.0 mmol, 118 mg) and ADN (4.0 equiv, 4.0 mmol, 496 mg) in MeCN (20 mL) was placed in the anodic compartment of the cell, and a solution of ADN (4.0 mmol, 496 mg) in MeCN (20 mL) was placed in the cathodic compartment of the cell. Solutions were electrolyzed using constant current conditions (*I* = 60 mA) at 23–25 °C under magnetic stirring. After passing 2.0 F/mol of electricity (reaction time 54 min), electrodes were washed with CH_2_Cl_2_ (2 × 20 mL). The organic phases from anodic and cathodic compartments were separately evaporated under water-jet vacuum. The yield of **2a** was determined according to ^1^H NMR spectroscopy using 1,1,2,2-tetrachloroethane as an internal standard.

**Reaction under constant potential electrolysis (experimental details for**Scheme 4**, reaction 2):** In a manner analogous to one described in [87], an undivided 10 mL electrochemical cell was equipped with a platinum plate anode (30 × 15 mm), a platinum wire cathode, and reference Ag/AgNO_3_ electrode linked to the solution by a porous glass diaphragm, and connected to a computer-assisted potentiostat. Electrodes were completely immersed in the solution given *S* = 4.5 cm^2^ of working anode surface. A mixture of 2-nitro-2-nitrosopropane (**1a**, 0.5 mmol, 59 mg), and ADN (4.0 equiv., 2.0 mmol, 248 mg) in MeCN (10 mL) was electrolyzed using constant potential conditions (*E_cell_* = 1.8 V vs Ag/AgNO_3_) at 23–25 °C under magnetic stirring. After passing 2.0 F per mol **1a** of electricity, electrodes were washed with CH_2_Cl_2_ (3 × 20 mL). The combined organic phase was washed with H_2_O (20 mL) and brine (20 mL), dried over Na_2_SO_4_, and solvent removed in vacuo. The yield of **2a** was determined according to ^1^H NMR spectroscopy using 1,1,2,2-tetrachloroethane as an internal standard.

**Computational details**: DFT computations were performed for 1 atm. and 298.15 K in Orca 6.1.0 package [93]. Results were visualized in Chemcraft 1.8 program. For conformationally flexible structures, generation of conformational ensembles was performed by GOAT algorithm [114] implemented in Orca: for closed shell species it was made using GFN2-xTB method [115] and for open-shell species by native ORCA 6.1.0 spin-polarized variant of GFN2-xTB method (“Native-spGFN2-xTB” keyword). ALPB(MeCN) solvation model [116] was used in both cases. Bond length constraints were used for the generation of conformer ensembles of transition states in order to avoid optimization to starting reagent(s) or product(s). On the next step, most stable conformers were identified by re-optimization of generated conformers and vibrational analysis on ωB97X-3c [94]/CPCM(MeCN) level of theory (in the case of >15 conformers, 15 most stable according to GFN2-xTB were analyzed).

## Supporting Information

File 1 General information on materials and instruments, experimental procedures, and characterization data for all compounds, XRD of **2c**, computational details, and copies of ^1^H, ^13^C, ^14^N, ^1^H,^13^C HSQC, and ^1^H,^13^C HMBC NMR spectra.

File 2 Crystallographic information for compound **2c**.

File 3 CheckCIF report for the data of **2c**.

## Data Availability

All data that supports the findings of this study is available in the published article and/or the supporting information of this article.
