# Supplementary material for: Sustainable electrochemical synthesis of aliphatic nitro-NNO-azoxy compounds employing ammonium dinitramide and their in vitro evaluation as potential nitric oxide donors and fungicides
Source: Beilstein J Org Chem. 2025 Dec 29;21:2739–54. doi: 10.3762/bjoc.21.211 (PMC12766168; doi:10.3762/bjoc.21.211)

BEILSTEIN JOURNAL OF ORGANIC CHEMISTRY

# Supporting Information

for

# Sustainable electrochemical synthesis of aliphatic nitro-*NNO*-azoxy compounds employing ammonium dinitramide and their in vitro evaluation as potential nitric oxide donors and fungicides

Alexander S. Budnikov, Nikita E. Leonov, Michael S. Klenov, Andrey A. Kulikov, Igor B. Krylov, Timofey A. Kudryashev, Aleksandr M. Churakov, Alexander O. Terent'ev and Vladimir A. Tartakovsky

*Beilstein J. Org. Chem.* **2025,** *21,* 2739–2754. doi:10.3762/bjoc.21.211

# General information on materials and instruments, experimental procedures, and characterization data for all compounds, XRD of 2c, computational details, and copies of $^{1}$H, $^{13}$C, $^{14}$N, $^{1}$H,$^{13}$C HSQC, and $^{1}$H,$^{13}$C HMBC NMR spectra

License and Terms: This is a supporting information file under the terms of the Creative Commons Attribution License (https://creativecommons.org/licenses/by/4.0). Please note that the reuse, redistribution and reproduction in particular requires that the author(s) and source are credited and that individual graphics may be subject to special legal provisions.
The license is subject to the *Beilstein Journal of Organic Chemistry* terms and conditions: (https://www.beilstein-journals.org/bjoc/terms)

## Table of contents


1. General information .......... S4
2. Experimental data .......... S5
3. Characterization of products .......... S7
4. Pictures of the equipment used .......... S11
5. Fungicidal activity tests (experimental details for Table 2). .......... S11
6. NO-release activity tests (experimental details for Figure 4). .......... S13
7. X-ray single-crystal diffraction: Structure determination of compound **2c** .......... S14
8. Computational details .......... S16
9. References .......... S24
10. NMR Data .......... S26
    10.1.1 $^{1}H$ NMR spectrum of compound **2b** [600.13 MHz, $CDCl_3$] .......... S26
    10.1.2 $^{13}C$ NMR spectrum of compound **2b** [150.90 MHz, $CDCl_3$] .......... S27
    10.1.3 {$^{1}H$–$^{13}C$} HSQC spectrum of compound **2b** [600.13 MHz, $CDCl_3$] .......... S28
    10.1.4 {$^{1}H$–$^{13}C$} HMBC spectrum of compound **2b** [600.13 MHz, $CDCl_3$] .......... S29
    10.1.5 $^{14}N$ NMR spectrum of compound **2b** [43.37 MHz, $CDCl_3$] .......... S30
    10.2.1 $^{1}H$ NMR spectrum of compound **2c** [500.13 MHz, $CDCl_3$] .......... S31
    10.2.2 $^{13}C$ NMR spectrum of compound **2c** [125.76 MHz, $CDCl_3$] .......... S32
    10.2.3 {$^{1}H$–$^{13}C$} HSQC spectrum of compound **2c** [500.13 MHz, $CDCl_3$] .......... S33
    10.2.4 $^{14}N$ NMR spectrum of compound **2c** [36.14 MHz, $CDCl_3$] .......... S34
    10.3.1 $^{1}H$ NMR spectrum of compound **2d** [500.13 MHz, $CDCl_3$] .......... S35
    10.3.2 $^{13}C$ NMR spectrum of compound **2d** [125.76 MHz, $CDCl_3$] .......... S36
    10.3.3 {$^{1}H$–$^{13}C$} HSQC spectrum of compound **2d** [500.13 MHz, $CDCl_3$] .......... S37
    10.3.4 {$^{1}H$–$^{13}C$} HMBC spectrum of compound **2d** [500.13 MHz, $CDCl_3$] .......... S38
    10.3.5 $^{14}N$ NMR spectrum of compound **2d** [43.37 MHz, $CDCl_3$] .......... S39
    10.4.1 $^{1}H$ NMR spectrum of compound **2e** [500.13 MHz, [$D_6$]acetone] .......... S40
    10.4.2 $^{13}C$ NMR spectrum of compound **2e** [125.76 MHz, [$D_6$]acetone] .......... S41
    10.4.3 {$^{1}H$–$^{13}C$} HSQC spectrum of compound **2e** [500.13 MHz, [$D_6$]acetone] .......... S42
    10.4.4 {$^{1}H$–$^{13}C$} HMBC spectrum of compound **2e** [500.13 MHz, [$D_6$]acetone] .......... S43
    10.4.5 $^{14}N$ NMR spectrum of compound **2e** [36.14 MHz, [$D_6$]acetone] .......... S44
    10.5.1 $^{1}H$ NMR spectrum of compound **2f** [500.13 MHz, [$D_6$]acetone] .......... S45
    10.5.2 $^{13}C$ NMR spectrum of compound **2f** [125.76 MHz, [$D_6$]acetone] .......... S46
    10.5.3 {$^{1}H$–$^{13}C$} HSQC spectrum of compound **2f** [500.13 MHz, [$D_6$]acetone] .......... S47
    10.5.4 {$^{1}H$–$^{13}C$} HMBC spectrum of compound **2f** [500.13 MHz, [$D_6$]acetone] .......... S48
    10.5.5 $^{14}N$ NMR spectrum of compound **2f** [36.14 MHz, [$D_6$]acetone] .......... S49

10.6.1 $^{1}H$ NMR spectrum of compound **2g** [600.13 MHz, $CDCl_3$] ............................S50
10.6.2 $^{13}C$ NMR spectrum of compound **2g** [150.90 MHz, $CDCl_3$] ...........................S51
10.6.3 {$^{1}H$–$^{13}C$} HSQC spectrum of compound **2g** [600.13 MHz, $CDCl_3$].................S52
10.6.4 {$^{1}H$–$^{13}C$} HMBC spectrum of compound **2g** [600.13 MHz, $CDCl_3$] ................S53
10.6.5 $^{14}N$ NMR spectrum of compound **2g** [43.37 MHz, $CDCl_3$] .............................S54
10.7.1 $^{1}H$ NMR spectrum of compound **2h** [600.13 MHz, $CDCl_3$] ............................S55
10.7.2 $^{13}C$ NMR spectrum of compound **2h** [150.90 MHz, $CDCl_3$] ...........................S56
10.7.3 {$^{1}H$–$^{13}C$} HSQC spectrum of compound **2h** [600.13 MHz, $CDCl_3$].................S57
10.7.4 {$^{1}H$–$^{13}C$} HMBC spectrum of compound **2h** [600.13 MHz, $CDCl_3$] ................S58
10.7.5 $^{14}N$ NMR spectrum of compound **2h** [43.14 MHz, $CDCl_3$] .............................S59
10.8.1 $^{1}H$ NMR spectrum of compound **2i** [500.13 MHz, $CDCl_3$].............................S60
10.8.2 $^{13}C$ NMR spectrum of compound **2i** [125.76 MHz, $CDCl_3$] ............................S61
10.8.3 {$^{1}H$–$^{13}C$} HSQC spectrum of compound **2i** [500.13 MHz, $CDCl_3$]..................S62
10.8.4 {$^{1}H$–$^{13}C$} HMBC spectrum of compound **2i** [500.13 MHz, $CDCl_3$]..................S63
10.8.5 $^{14}N$ NMR spectrum of compound **2i** [36.14 MHz, $CDCl_3$] .............................S64
10.9.1 $^{1}H$ NMR spectrum of compound **3f** [500.13 MHz, [$D_6$]acetone].....................S65
10.9.2 $^{13}C$ NMR spectrum of compound **3f** [125.76 MHz, [$D_6$]acetone] ...................S66
10.9.3 {$^{1}H$–$^{13}C$} HSQC spectrum of compound **3f** [500.13 MHz, [$D_6$]acetone].........S67
10.9.4 {$^{1}H$–$^{13}C$} HMBC spectrum of compound **3f** [500.13 MHz, [$D_6$]acetone].........S68
10.9.5 $^{14}N$ NMR spectrum of compound **3f** [36.14 MHz, [$D_6$]acetone] .....................S69
10.10.1 $^{1}H$ NMR spectrum of compound **4f** [500.13 MHz, $CDCl_3$] ...........................S70
10.10.2 $^{13}C$ NMR spectrum of compound **4f** [125.76 MHz, $CDCl_3$] ..........................S71
10.10.3 {$^{1}H$–$^{13}C$} HSQC spectrum of compound **4f** [500.13 MHz, $CDCl_3$]................S72
10.10.4 {$^{1}H$–$^{13}C$} HMBC spectrum of compound **4**f [500.13 MHz, $CDCl_3$]................S73
10.10.5 $^{14}N$ NMR spectrum of compound **4f** [36.14 MHz, $CDCl_3$] ............................S74

## 1. General information

### Safety precautions [1–4]

**Although we have encountered no difficulties during preparation and handling of compounds described and used in this paper, they are explosive energetic materials which are sensitive to impact and friction. Mechanical actions of these energetic materials, involving scratching or scraping, must be avoided. Experimental procedures involving ammonium dinitramide impose a potential risk of detonation. Ammonium dinitramide is a sensitive explosive and should be handled with care, light and moisture exposure and should be avoided during storage due to its photosensitivity and hygroscopic nature. When working with it, it's important to protect your face with a face shield and your hands with Kevlar gloves. More detailed information on handling and properties of ADN can be found in specialized literature** [1–4].

$^{1}$H, $^{13}$C, $^{14}$N NMR spectra were recorded with Bruker DRX-500 (500.1, 125.8, 36.1 MHz, respectively) and Bruker AV600 (600.1, 150.9, 43.4 MHz, respectively) spectrometers. *Chemical shifts* are reported in delta ($\delta$) *units*, parts per million (ppm) downfield from internal TMS ($^{1}$H, $^{13}$C) or external $CH_3NO_2$ ($^{14}$N negative values of $\delta_N$ correspond to upfield shifts). The IR spectra were recorded with a Bruker ALPHA-T spectrometer in the range 400–4000 cm$^{-1}$ (resolution 2 cm$^{-1}$) as pellets with KBr or as a thin layer. High-resolution ESI mass spectra (HRMS) were recorded with a Bruker micrOTOF II instrument. Silica gel 60 Merck (15–40 μm) was used for preparative column and thin-layer chromatography. Silica gel “Silpearl UV 254” was used for preparative column and thin-layer chromatography. Analytical thin-layer chromatography (TLC) was carried out on Merck silica gel 60 F254 and “Silufol” TLC silica gel UV-254 aluminum sheets. All reagents were purchased from Acros and Sigma-Aldrich. *Solvents were purified* before use, according to standard procedures. All other reagents were used without further purification. Ammonium dinitramide,[5] 2-nitro-2-nitrosopropane (**1a**),[6] 1-nitro-1-nitrosocyclopentane (**1b**),[7] 1-nitro-1-nitrosocyclohexane (**1c**),[8] 2-nitro-2-nitroso-1,3-diphenylpropane (**1e**),[8] 2,2-dimethyl-5-nitro-5-nitroso-1,3-dioxane (**1f**),[9] ethyl 2-nitro-2-nitrosopropanoate (**1h**),[10] 1-nitrosocyclohexane-1-carbonitrile (**1i**),[11] were prepared according to the reported procedures.

## 2. Experimental data

### 2.1 Synthesis of the 1-nitro-1-nitrosocycloheptane (1d)

1-Nitrocycloheptane (0.50 g, 3.50 mmol) was added to the stirred solution of NaOH (0.17 g, 4.25 mmol) in $H_2O$ (6 mL) at 25 °C. Then the mixture was heated up to 60 °C and stirred at this temperature until the complete dissolution of the 1-nitrocycloheptane. After that the reaction mixture was cooled to 25 °C and $NaNO_2$ (0.36 g, 5.25 mmol) was added. After addition, the mixture was cooled to –10 °C and conc. HCl (1.5 mL, 10.50 mmol) was added dropwise with vigorous stirring. Then the resulting green solution was extracted with hexane (3 × 50 mL). The combined organic extracts were washed with water (25 mL), brine (25 mL), dried with anhydrous $Na_2SO_4$ and concentrated under reduced pressure. Product **1d** (0.56 g, 93%) was obtained as a green oil and was used in further reactions without additional purification.

### 2.2 Synthesis of the methyl 2-nitro-2-nitrosopropanoate (1g)

A solution of $N_2O_4$ (0.74 mg, 8.00 mmol) in $Et_2O$ (5 mL) was added dropwise for 1–2 min to a stirred solution of methyl pyruvate oxime (0.94 mg, 8.00 mmol) in $Et_2O$ (25 mL) at –5 °C. Then the mixture was stirred at this temperature for 1 hour. The reaction mixture was rotary evaporated and the resulting nitroso compound was used in further reactions without additional purification.

### 2.3 Procedure for deprotection of 2f (experimental details for Scheme 3, reaction 2).

Acetyl chloride (4.5 mL, 63.3 mmol) was added dropwise to a stirred solution of 2,2-dimethyl-5-nitro-5-(nitro-*NNO*-azoxy)-1,3-dioxane (**2f**, 1.00 g, 4.0 mmol) in MeOH (10 mL) at 25 °C. After addition, the reaction mixture was stirred at this temperature for 24 h (the completion of reaction was monitored by TLC). Then the solvent was removed in vacuo at 40 °C. Product **3f** (0.79 g, 3.76 mmol, 94%) was isolated by column chromatography on silica gel ($R_f$ = 0.40, petroleum ether/ethyl acetate, 3:1).

### 2.4 Procedure for nitration of 3f (experimental details for Scheme 3, reaction 2).

2-Nitro-2-(nitro-*NNO*-azoxy)-1,3-propanediol **3f** (0.63 g, 3.0 mmol) was added in portions to a mixture of acetic anhydride (3.4 mL, 36.0 mmol) and 100% nitric acid (0.6 mL, 13.2 mmol) at 0 °C, and the mixture was stirred at this temperature for 30 min. Then the reaction mixture was poured into ice-water (50 mL) and extracted with $CH_2Cl_2$ (3 × 20 mL). The combined organic phase was washed with water (30 mL), brine (30 mL), dried over $Na_2SO_4$ and solvent removed in vacuo. Product **4f** (0.71 g, 2.37 mmol, 79%) was isolated by column chromatography on silica gel ($R_f$ = 0.55, petroleum ether/ethyl acetate, 5:1).

## 2.5 Cyclic voltammetry studies

Cyclic voltammetry (CV) studies were implemented in a manner analogous to one described in [12] on an PS-30 computer-assisted potentiostat-galvanostat manufactured by «SmartStat», the scan rate was 100 mV·s$^{-1}$. The experiments were performed in a 10 mL five-neck glass conic electrochemical cell with a water jacket. CV curves were recorded using a three-electrode scheme. In a typical case, 5 mL of a solution of analyte (0.01 M) and *n*-$Bu_4NBF_4$ (0.1 M) was utilized. The working electrode was a disc glassy-carbon electrode (*d* = 3 mm). A platinum plate served as an auxiliary electrode. An $Ag/AgNO_3$ (0.01 M) in 0.1 M *n*-$Bu_4NBF_4$/MeCN electrode was used as the reference electrode and was linked to the solution by a porous glass diaphragm. The solutions were kept under thermally controlled conditions at 21 ± 0.5 °C and deaerated by bubbling argon. Electrochemical experiments were performed under an argon atmosphere. The working electrode was polished before recording each CV curve.

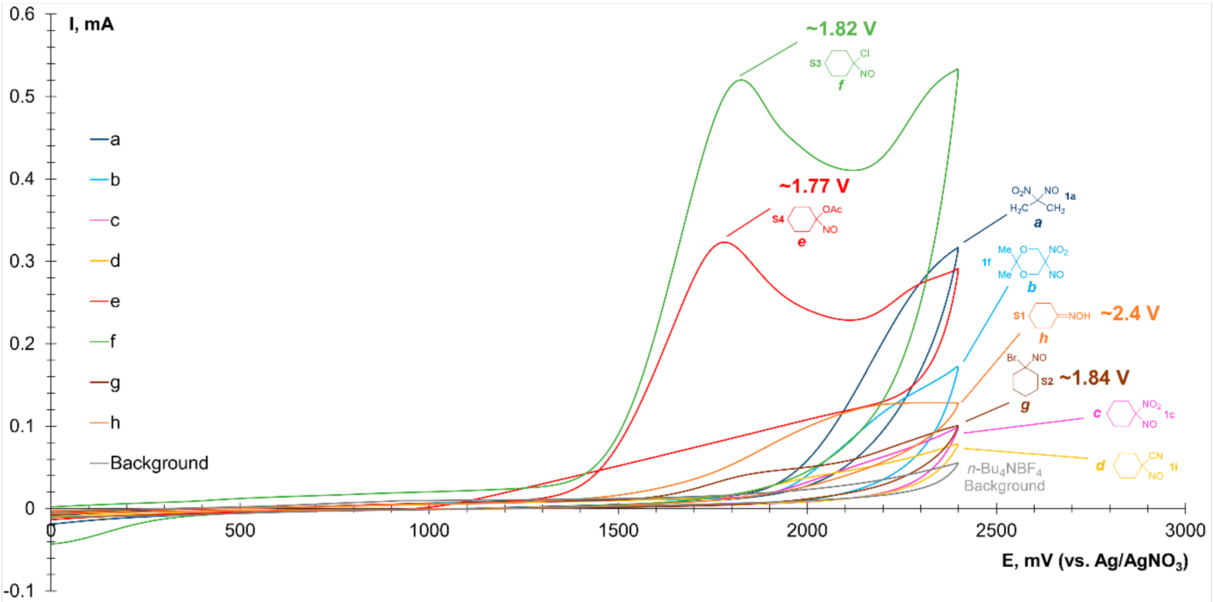


**Figure S1.** CV-curves of 0.01 M solutions of a) **1a** (blue), b) **1f** (azure), c) **1c** (pink), d) **1i** (yellow), e) **S4** (red), f) S3 (green), g) **S2** (brown), and h) **S1** (orange) in 0.1 M *n*-$Bu_4NBF_4$ solution in MeCN on a working glassy-carbon electrode (*d* = 3 mm) under a scan rate of 0.1 V·s$^{-1}$ at 298 K.

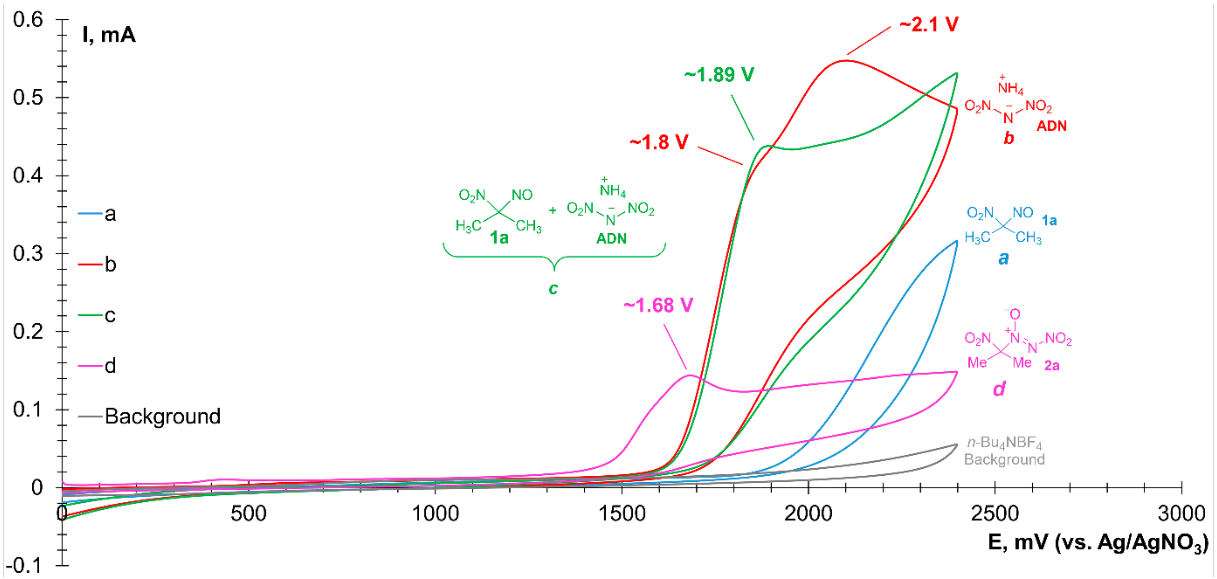


**Figure S2.** CV-curves of 0.01 M solutions of a) **1a** (blue), b) ADN (red), c) the mixture of **1a** and ADN (green), d) **2a** (pink) in 0.1M *n*-$Bu_4NBF_4$ solution in MeCN on a working glassy-carbon electrode (*d* = 3 mm) under a scan rate of 0.1 V·$s^{-1}$ at 298 K.

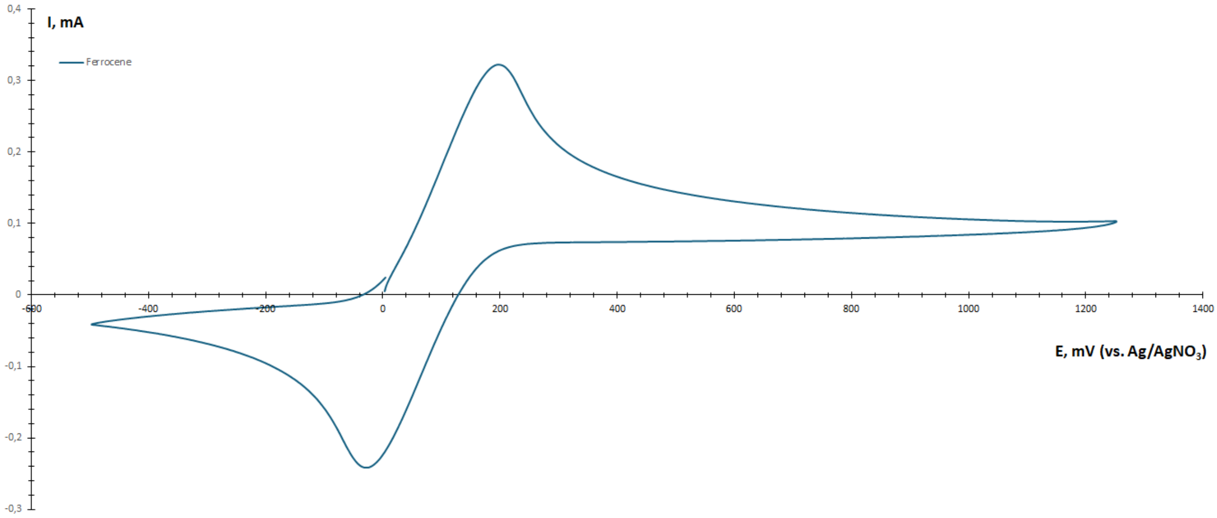


**Figure S3.** CV-curve of 0.01 M solution of ferrocene in 0.1 M *n*-$Bu_4NBF_4$ solution in MeCN on a working glassy-carbon electrode (*d* = 3 mm) under a scan rate of 0.1 V·$s^{-1}$ at 298 K.

## 3. Characterization of products

**1-Nitro-1-(nitro-*NNO*-azoxy)cyclopentane (2b)**: Yellowish oil. 41% yield. $R_f$ (petroleum ether) = 0.11. $^1$H NMR (600.13 MHz, $CDCl_3$)$\delta$: 2.03–2.09 (m, 4H, H(3, 4)), 2.84–2.93 (m, 4H, H(2, 5)) ppm. $^{13}$C NMR (150.90 MHz, $CDCl_3$)$\delta$: 24.8 (s, C(3, 4)), 37.8 (s, C(2, 5)), 122.0 (br. s, C(1)) ppm. The $^1$H–$^{13}$C HSQC and HMBC experiments were used to assign the signals. $^{14}$N NMR (43.37 MHz, $CDCl_3$)$\delta$: –6 (C–$NO_2$, $\Delta\nu_{1/2}$ = 45 Hz), –39 (N(O)=N–<u>N</u>$O_2$, $\Delta\nu_{1/2}$ = 15 Hz), –43 (<u>N</u>(O)=N–$NO_2$, $\Delta\nu_{1/2}$ = 50 Hz) ppm.

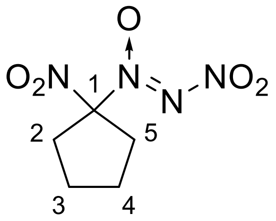

IR (KBr): $\nu$ = 2965 (w), 2889 (w), 1621 (s), 1573 (s), 1508 (s), 1434 (m), 1353 (m), 1299 (s), 1276 (s), 1199 (w), 942 (w), 844 (s) cm$^{-1}$. Elemental analysis calcd (%) for $C_5H_8N_4O_5$: C 29.42, H 3.95, N 27.45; found: C 29.46, H 3.98, N 27.40.

**1-Nitro-1-(nitro-*NNO*-azoxy)cyclohexane (2c)**: Pale yellow crystals, m.p. 37–38 °C.

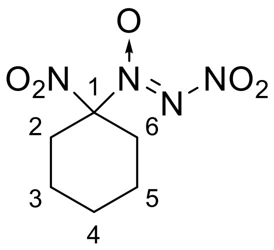


70% yield. $R_f$ (petroleum ether) = 0.15. $^1$H NMR (500.13 MHz, $CDCl_3$)$\delta$: 1.57–1.61 (m, 1H, H(4)), 1.69–1.75 (m, 3H, H(3, 4, 5)), 1.83–1.84 (m, 2H, H(3, 5)), 2.59–2.72 (m, 4H, H(2, 6)) ppm. $^{13}$C NMR (125.76 MHz, $CDCl_3$)$\delta$: 21.7 (s, C(3, 5)), 22.8 (s, C(4)), 32.2 (s, C(2, 6)), 114.9 (br. s, C(1)) ppm. The $^1$H–$^{13}$C HSQC experiment was used to assign the signals. $^{14}$N NMR (36.14 MHz, $CDCl_3$)$\delta$: –5 (C–$NO_2$, $\Delta\nu_{1/2}$ = 60 Hz), –38 (N(O)=N–<u>N</u>$O_2$, $\Delta\nu_{1/2}$ = 15 Hz), –43 (<u>N</u>(O)=N–$NO_2$, $\Delta\nu_{1/2}$ = 60 Hz) ppm. IR (KBr): $\nu$ = 2955 (w), 2938 (w), 2872 (w), 1623 (s), 1565 (s), 1511 (m), 1453 (w), 1431 (w), 1371 (w), 1297 (m), 1256 (m), 1147 (m), 847 (m) cm$^{-1}$. Elemental analysis calcd (%) for $C_6H_{10}N_4O_5$: C 33.03, H 4.62, N 25.68; found: C 33.09, H 4.64, N 25.57.

**1-Nitro-1-(nitro-*NNO*-azoxy)cycloheptane (2d)**: Yellowish oil. 25% yield. $R_f$ (petroleum

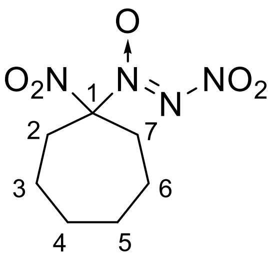


ether) = 0.32. $^1$H NMR (500.13 MHz, $CDCl_3$)$\delta$: 2.51 (s, 3H, Me), 7.36–7.39 (m, 2H, H(4), H(6)), 7.52 (t, 1H, H(5), $^3J_{HH}$ = 7.5 Hz), 7.69 (d, 1H, H(3), $^3J_{HH}$ = 8.0 Hz) ppm. $^{13}$C NMR (125.76 MHz, $CDCl_3$)$\delta$: 22.8 (s, C(3, 6)), 28.9 (s, C(4, 5)), 36.3 (s, C(2, 7)), 119.7 (br. s, C(1)) ppm. The $^1$H–$^{13}$C HSQC and HMBC experiments were used to assign the signals. $^{14}$N NMR (43.37 MHz, $CDCl_3$)$\delta$: –3 (C–$NO_2$, $\Delta\nu_{1/2}$ = 85 Hz), –38 (N(O)=N–<u>N</u>$O_2$, $\Delta\nu_{1/2}$ = 15 Hz), –39 (<u>N</u>(O)=N–$NO_2$, $\Delta\nu_{1/2}$ = 110 Hz) ppm. IR (KBr): $\nu$ = 2940 (m), 2867 (w), 1628 (s), 1574 (s), 1509 (s), 1461 (m), 1336 (m), 1295 (s), 1261 (m), 846 (m) cm$^{-1}$. Elemental analysis calcd (%) for $C_7H_{12}N_4O_5$: C 36.21, H 5.21, N 24.13; found: C 36.24, H 5.25, N 24.05.

**2-Nitro-2-(nitro-*NNO*-azoxy)-1,3-diphenylpropane (2e)**: beige crystals, m.p. 92–93 °C

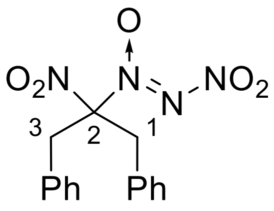


(dec.). 57% yield. $R_f$ (petroleum ether) = 0.21. $^1$H NMR (500.13 MHz, [$D_6$]acetone)$\delta$: 3.86 (s, 4H, H(1, 3)), 7.30–7.43 (m, 10H, Ph) ppm. $^{13}$C NMR (125.76 MHz, [$D_6$]acetone)$\delta$: 40.8 (s, C(1, 3)), 124.6 (br. s, C(2)), 129.5–131.7 (Ph) ppm. The $^1$H–$^{13}$C HSQC and HMBC experiments were used to assign the signals. $^{14}$N NMR (36.14 MHz, [$D_6$]acetone)$\delta$: –8 (C–$NO_2$, $\Delta\nu_{1/2}$ = 50 Hz), –38 (N(O)=N–<u>N</u>$O_2$, $\Delta\nu_{1/2}$ = 35 Hz), –43 (<u>N</u>(O)=N–$NO_2$, $\Delta\nu_{1/2}$ = 135 Hz) ppm. IR (KBr): $\nu$ = 1619 (m), 1576 (s), 1558 (s), 1511 (m), 850 (w),

700 (m) cm$^{-1}$. Elemental analysis calcd (%) for $C_{15}H_{14}N_4O_5$: C 54.55, H 4.27, N 16.96; found: C 54.58, H 4.30, N 16.81.

**2,2-Dimethyl-5-nitro-5-(nitro-*NNO*-azoxy)-1,3-dioxane (2f)**: Light yellow crystals, m.p. 50–51 °C. 68% yield. $R_f$ (petroleum ether/ethyl acetate, 40:1) = 0.15. $^1$H NMR (500.13 MHz, [D$_6$]acetone)*δ*: 1.51, 1.56 (s, 6H, Me), 4.94 (s, 4H, H(4, 6)) ppm. $^{13}$C NMR (125.76 MHz, [D$_6$]acetone)*δ*: 22.5, 24.1 (s, Me), 62.4 (s, C(4, 6)), 101.6 (s, C(2)), 107.1 (br. s, C(5)) ppm. The $^1$H–$^{13}$C HSQC and HMBC experiments were used to assign the signals. $^{14}$N NMR (36.14 MHz, [D$_6$]acetone)*δ*: 5 (N(O)=N–NO$_2$, $\Delta\nu_{1/2}$ = 1000 Hz) –15 (C–NO$_2$, $\Delta\nu_{1/2}$ = 50 Hz), –39 (N(O)=N–NO$_2$, $\Delta\nu_{1/2}$ = 15 Hz), –50 (N(O)=N–NO$_2$, $\Delta\nu_{1/2}$ = 50 Hz) ppm. IR (KBr): ν = 1638 (s), 1581 (s), 1515 (m), 1384 (w), 1275 (m), 1104 (m), 1056 (w), 843 (m), 813 (s) cm$^{-1}$. Elemental analysis calcd (%) for $C_6H_{10}N_4O_7$: C 28.81, H 4.03, N 22.40; found: C 28.82, H 4.05, N 22.27.

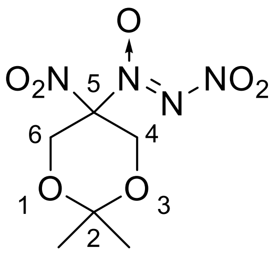


**Methyl 2-nitro-2-(nitro-*NNO*-azoxy)propanoate (2g)**: Pale yellow oil. 38% yield. $R_f$ (petroleum ether/ethyl acetate, 10:1) = 0.25. $^1$H NMR (600.13 MHz, CDCl$_3$)*δ*: 2.48 (s, 3H, Me), 4.04 (s, 3H, OMe) ppm. $^{13}$C NMR (150.90 MHz, CDCl$_3$)*δ*: 21.5 (s, Me), 56.6 (s, OMe), 111.5 (br. s, C(2)), 159.6 (s, C(1)) ppm. The $^1$H–$^{13}$C HSQC and HMBC experiments were used to assign the signals. $^{14}$N NMR (43.37 MHz, CDCl$_3$)*δ*: 4 (N(O)=N–NO$_2$, $\Delta\nu_{1/2}$ = 900 Hz), –20 (C–NO$_2$, $\Delta\nu_{1/2}$ = 35 Hz), –43 (N(O)=N–NO$_2$, $\Delta\nu_{1/2}$ = 30 Hz), –52 (N(O)=N–NO$_2$, $\Delta\nu_{1/2}$ = 40 Hz) ppm. IR (KBr): ν = 2960 (m), 2923 (s), 2853 (m), 1773 (s), 1631 (s), 1589 (s), 1518 (m), 1443 (m), 1387 (m), 1342 (m), 1292 (m), 1158 (m), 1123 (m), 842 (m) cm$^{-1}$. Elemental analysis calcd (%) for $C_4H_6N_4O_7$: C 21.63, H 2.72, N 25.22; found: C 21.67, H 2.76, N 25.05.

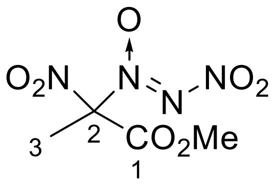


**Ethyl 2-nitro-2-(nitro-*NNO*-azoxy)propanoate (2h)**: Pale yellow oil. 61% yield. $R_f$ (petroleum ether/ethyl acetate, 10:1) = 0.25. $^1$H NMR (600.13 MHz, CDCl$_3$)*δ*: 1.39 (t, 3H, CH$_2$CH$_3$, $^3J_{HH}$ = 7.2 Hz), 2.47 (s, 3H, Me), 4.49 (q, 2H, CH$_2$CH$_3$, $^3J_{HH}$ = 7.2 Hz) ppm. $^{13}$C NMR (150.90 MHz, CDCl$_3$)*δ*: 13.6 (s, CH$_2$CH$_3$), 20.8 (s, Me), 66.2 (s, CH$_2$CH$_3$), 111.0 (br. s, C(2)), 158.4 (s, C(1)) ppm. The $^1$H–$^{13}$C HSQC and HMBC experiments were used to assign the signals. $^{14}$N NMR (43.37 MHz, CDCl$_3$)*δ*: –19 (C–NO$_2$, $\Delta\nu_{1/2}$ = 45 Hz), –42 (N(O)=N–NO$_2$, $\Delta\nu_{1/2}$ = 30 Hz), –51 (N(O)=N–NO$_2$, $\Delta\nu_{1/2}$ = 50 Hz) ppm. IR (KBr): ν = 2957 (m), 2926 (m), 2855 (m), 1770 (s), 1634 (s), 1591 (s), 1520 (m), 1445 (m), 1387 (m), 1341

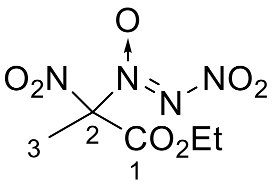

(m), 1296 (s), 1271 (s), 1177 (m), 1157 (m), 1120 (m), 1007 (m), 849 (m) cm$^{-1}$. Elemental analysis calcd (%) for $C_5H_8N_4O_7$: C 25.43, H 3.41, N 23.73; found: C 25.47, H 3.43, N 23.57.

**1-(Nitro-*NNO*-azoxy)cyclohexane-1-carbonitrile (2i)**: Yellowish oil. 61% yield. $R_f$ (petroleum ether) = 0.10. $^1$H NMR (500.13 MHz, $CDCl_3$)$\delta$: 1.31–1.38 (m, 1H, H(4)), 1.65–1.86 (m, 3H, H(3, 4, 5)), 1.97–2.06 (m, 2H, H(3, 5)), 2.17–2.50 (m, 4H, H(2, 6)) ppm. $^{13}$C NMR (125.76 MHz, $CDCl_3$)$\delta$: 22.2 (s, C(3, 5)), 23.1 (s, C(4)), 34.3 (s, C(2, 6)), 79.2 (br. s, C(1)), 113.5 (s, CN) ppm. The $^1$H–$^{13}$C HSQC and HMBC experiments were used to assign the signals. $^{14}$N NMR (36.14 MHz, $CDCl_3$)$\delta$: –37 (N(O)=N–$\underline{N}O_2$ & $\underline{N}$(O)=N–$NO_2$, $\Delta\nu_{1/2}$ = 25 Hz), –117 (CN, $\Delta\nu_{1/2}$ = 950 Hz) ppm. IR (KBr): $\nu$ = 2950 (m), 2869 (m), 1618 (s), 1564 (m), 1507 (s), 1455 (m), 1298 (s), 1268 (s), 1170 (w), 847 (m) cm$^{-1}$. Elemental analysis calcd (%) for $C_7H_{10}N_4O_3$: C 42.42, H 5.09, N 28.27; found: C 42.47, H 5.13, N 28.16.

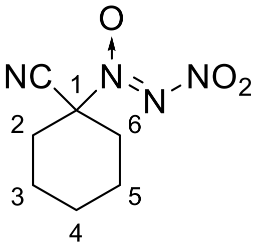


**2-Nitro-2-(nitro-*NNO*-azoxy)-1,3-propanediol (3f)**: Pale yellow solid. $R_f$ (petroleum ether/ethyl acetate, 3:1) = 0.40. $^1$H NMR (500.13 MHz, [D$_6$]acetone)$\delta$: 4.59 (dd, 4H, H(1, 3), $^2J_{HH}$ = 24.5 Hz, $^3J_{HH}$ = 5.4 Hz), 5.70 (br. s, 2H, OH) ppm. $^{13}$C NMR (125.76 MHz, [D$_6$]acetone)$\delta$: 59.4 (s, C(1, 3), 113.6 (br. s, C(2)) ppm. The $^1$H–$^{13}$C HSQC and HMBC experiments were used to assign the signals. $^{14}$N NMR (36.14 MHz, [D$_6$]acetone)$\delta$: –12 (C–$NO_2$, $\Delta\nu_{1/2}$ = 65 Hz), –38 (N(O)=N–$\underline{N}O_2$, $\Delta\nu_{1/2}$ = 20 Hz), –47 ($\underline{N}$(O)=N–$NO_2$, $\Delta\nu_{1/2}$ = 60 Hz) ppm. IR (KBr): $\nu$ = 3563 (m), 3384 (s), 2958 (w), 2902 (w), 1625 (s), 1577 (s), 1513 (s), 1456 (m), 1339 (m), 1298 (s), 1267 (s), 1071 (s), 1041 (m), 973 (m), 845 (s) cm$^{-1}$. Elemental analysis calcd (%) for $C_3H_6N_4O_7$: C 17.15, H 2.88, N 26.67; found: C 17.17, H 2.89, N 26.53.

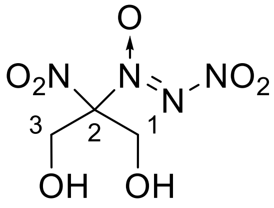


**2-Nitro-2-(nitro-*NNO*-azoxy)-propane-1,3-diyl dinitrate (4f):** Yellow oil. $R_f$ (petroleum ether/ethyl acetate, 5:1) = 0.55. $^1$H NMR (500.13 MHz, $CDCl_3$)$\delta$ = 5.47 (dd, 4H, H(1, 3), $^2J_{HH}$ = 13.0 Hz, $^3J_{HH}$ = 7.0 Hz) ppm. $^{13}$C NMR (125.76 MHz, $CDCl_3$)$\delta$ = 65.1 (s, C(1, 3)), 105.8 (br. s, C(2)) ppm. The $^1$H–$^{13}$C HSQC and HMBC experiments were used to assign the signals. $^{14}$N NMR (36.14 MHz, $CDCl_3$)$\delta$ = –23 (C–$NO_2$, $\Delta\nu_{1/2}$ = 45 Hz), –46 (N(O)=N–$\underline{N}O_2$, $\Delta\nu_{1/2}$ = 30 Hz), –56 ($\underline{N}$(O)=N–$NO_2$ & $ONO_2$, $\Delta\nu_{1/2}$ = 110 Hz) ppm. IR (KBr): $\nu$ = 3027 (w), 2978 (w), 2946 (w), 1674 (s), 1637 (s), 1593 (s), 1521 (s), 1332 (m), 1286

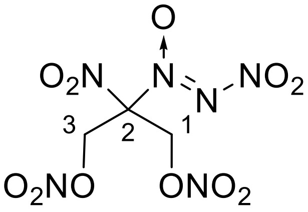

(s), 1029 (s), 831 (s) cm$^{-1}$. Elemental analysis calcd (%) for $C_3H_4N_6O_{11}$: C 12.01, H 1.34, N 28.01; found: C 12.08, H 1.37, N 27.89.

## 4. Pictures of the equipment used

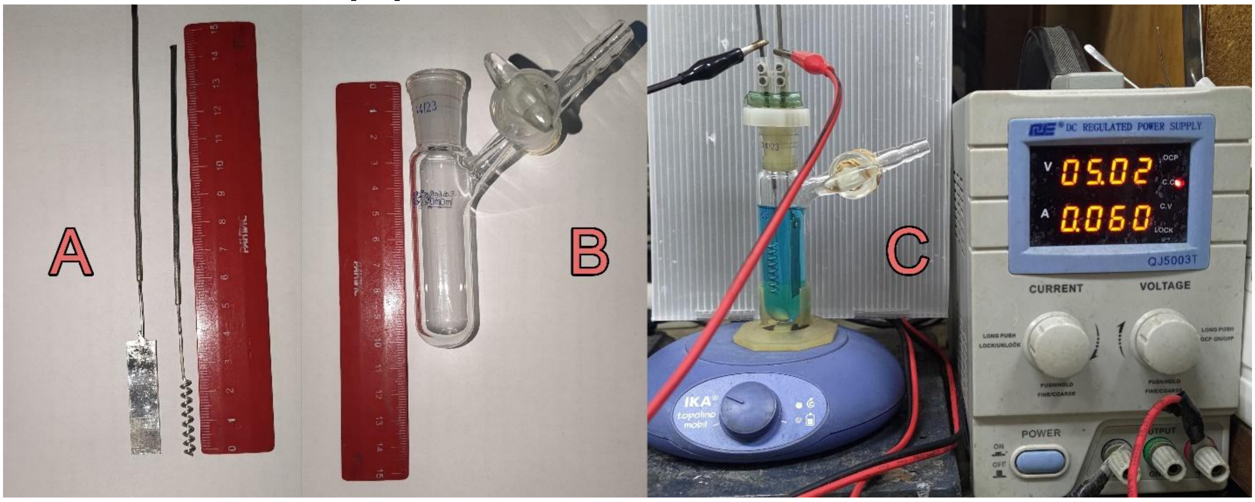


**Figure S4.** (A) Electrodes used in the reaction – platinum plate (left) and platinum wire (right). (B) Reaction vessel. (C) Assembled electrochemical setup.

## 5. Fungicidal activity tests (experimental details for Table 2).

Fungicidal activity assay was performed in a manner analogous to one described in [12]. The strains used in this work were obtained from the collection of the All-Russian Research Institute for Phytopathology (B. Vyazemy, Moscow reg., Russia).

Fungicidal activity was measured against six phytopathogenic fungi from different taxonomic classes (*V.i.* — Venturia inaequalis MRA-16-2, *R.s.* — Rhizoctonia solani 100063, *F.o.* — Fusarium oxysporum FO-8, *F.m.* — Fusarium moniliforme 100146, *B.s.* — Bipolaris sorokiniana MRB(V)-1, *S.s.* — Sclerotinia sclerotiorum 100033) using the standard poison food technique [13–19]. The tested substances were dissolved in acetone (1 mg·mL$^{-1}$) and incorporated into liquid sugar-potato agar at 50–55 °C to achieve a final concentration of 10 mg·L$^{-1}$. The agar-substance mixture was poured into sterile Petri dishes and allowed to cool to room temperature. Mycelial pieces from the peripheral growth zone of 3–5 day old fungal cultures were transferred to the test dishes using a needle. Colonies grown in medium with acetone alone served as controls. After 72 hours, the diameters of the fungal colonies were measured. Each experiment was repeated 3 times, except for tests with *V. inaequalis* which had 5 replicates. Mycelial growth suppression was calculated as $((D_c – D_S)/D_c) \times 100\%$, where $D_c$ is the average control colony diameter and $D_S$ is the average colony diameter in the presence of the tested substance.

**Table S1.** Fungicidal activity of the obtained compounds **2a–i**, **3f**, **4f** and Triadimefon.

| No. | Compound | Mycelium Growth Inhibition (%) | | | | | |
|---|---|---|---|---|---|---|---|
| | | *V. i.* | *R. s.* | *F. o.* | *F. m.* | *B. s.* | *S. s.* |
| 1 | 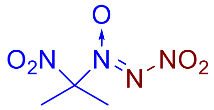 **a** | 6 | 89 | 33 | 94 | 11 | 21 |
| 2 | 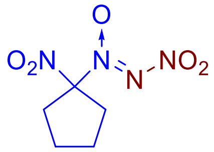 **2b** | 7 | 24 | 4 | 11 | 15 | 8 |
| 3 | 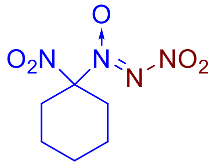 **2c** | 4 | 11 | 11 | 77 | 11 | 14 |
| 4 | 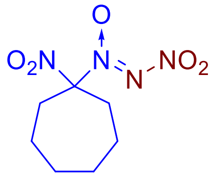 **2d** | | 29 | 8 | 7 | 22 | 8 |
| 5 | 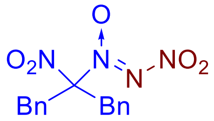 **2e** | 13 | 57 | 16 | 32 | 21 | 17 |
| 6 | 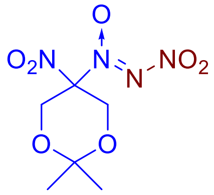 **2f** | 6 | 2 | 0 | 5 | 3 | 10 |
| 7 | 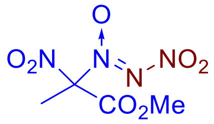 **2g** | | 22 | 5 | 6 | 15 | 15 |
| 8 | 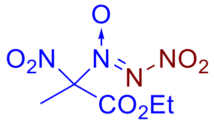 **2h** | 0 | 34 | 5 | 11 | 14 | 11 |
| 9 | 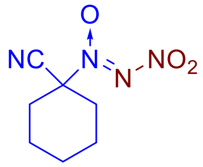 | 100 | 100 | 91 | 100 | 64 | 46 |

| | | | | | | | |
|---|---|---|---|---|---|---|---|
| | **2i** | | | | | | |
| 10 | 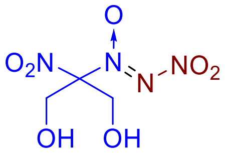 **3f** | 0 | 13 | 6 | 0 | -5 | 4 |
| 11 | 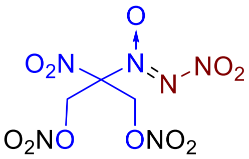 **4f** | 25 | 52 | 40 | 81 | 48 | 22 |
| 12 | 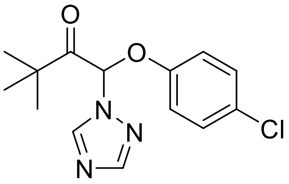 (±)-triadimefon (reference compound) | **41** | **43** | **77** | **87** | **44** | **61** |

[a]The data on fungicidal activity exceeding the standard (triadimefon) are highlighted in green. Concentration of compounds in nutrient medium 30 mg·L$^{-1}$.

## 6. NO-release activity tests (experimental details for Figure 4).

In a manner analogous to one described in [20], the test molecule (0.1 mmol) was dissolved in DMSO (50 mL). 20 µL aliquot of the resulted solution was diluted with phosphate buffer solution (180 µL, pH 7.4). The final concentration of the tested compound was 2·10$^{-4}$ M. The mixture was incubated at 37 °C for 1 h. 50 µL aliquot of the Griess reagent (prepared by mixing sulfanilamide (4 g), *N*-naphthylethylenediamine dihydrochloride (0.2 g) and 85% $H_3PO_4$ (10 mL) in distilled and deionized water (final volume 100 mL)) was added and incubated for 10 min at 37 °C. UV absorbance at 540 nm was measured using a Multiskan GO Microplate Photometer and calibrated using a standard curve prepared from standard solutions of $NaNO_2$ to give the nitrite concentration. All measurements were made in triplicate.

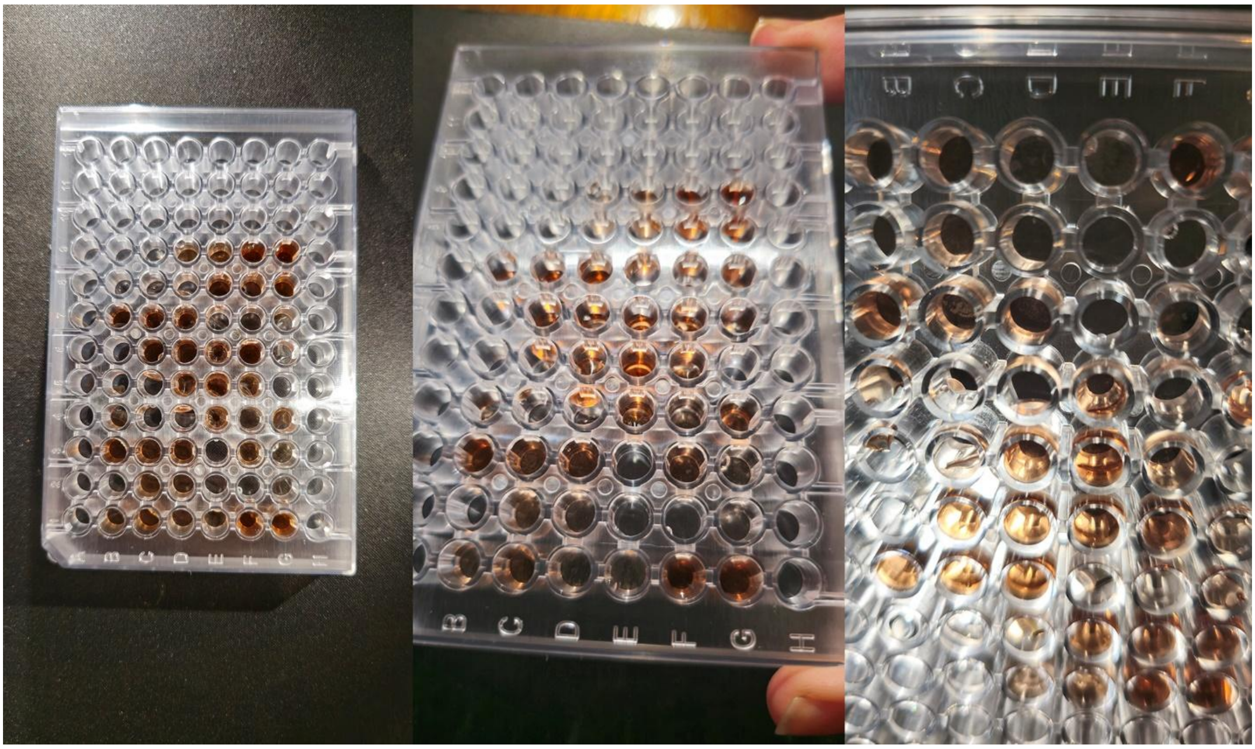

**Figure S5.** Disposition of standards and samples in a 96-microwell plate using the Griess assay method.

### 7. X-ray single-crystal diffraction: Structure determination of compound 2c

X-ray diffraction data were collected at 100K on a four-circle Rigaku Synergy S diffractometer equipped with a HyPix6000HE area-detector (kappa geometry, shutterless ω-scan technique), using monochromatized Cu $K_{\alpha}$-radiation. The intensity data were integrated and corrected for absorption and decay by the CrysAlisPro program [21]. The structure was solved by direct methods using SHELXT [22] and refined on $F^2$ using SHELXL-2018 [23] in the OLEX2 program [24]. All non-hydrogen atoms were refined with individual anisotropic displacement parameters. All hydrogen atoms were placed in ideal calculated positions and refined as riding atoms with relative isotropic displacement parameters. A rotating group model was applied for methyl groups.

**Table S2.** Crystal data and structure refinement for **2c**.

| | | |
|---|---|---|
| Empirical formula | $C_6H_{10}N_4O_5$ | |
| Formula weight | 218.18 | |
| Temperature | 100(2) K | |
| Wavelength | 1.54184 Å | |
| Crystal system | Triclinic | |
| Space group | P-1 | |

| | | |
|---|---|---|
| Unit cell dimensions | a = 8.52830(10) Å | α = 74.0540(10)° |
| | b = 10.54370(10) Å | β = 89.7980(10)° |
| | c = 10.9886(2) Å | γ = 86.7860(10)° |
| Volume | 948.51(2) $Å^3$ | |
| Z | 4 | |
| Density (calculated) | 1.528 $Mg/m^3$ | |
| Absorption coefficient | 1.163 $mm^{-1}$ | |
| F(000) | 456 | |
| Crystal size | 0.47 x 0.3 x 0.23 $mm^3$ | |
| Theta range for data collection | 4.185 to 79.655° | |
| Index ranges | -10<=h<=10, -13<=k<=13, -14<=l<=13 | |
| Reflections collected | 25159 | |
| Independent reflections | 4086 [R(int) = 0.0324] | |
| Completeness to theta = 67.684° | 99.9 % | |
| Absorption correction | Gaussian | |
| Max. and min. transmission | 1.000 and 0.232 | |
| Refinement method | Full-matrix least-squares on $F^2$ | |
| Data / restraints / parameters | 4086 / 29 / 320 | |
| Goodness-of-fit on $F^2$ | 1.054 | |
| Final R indices [I>2sigma(I)] | R1 = 0.0387, wR2 = 0.0944 | |
| R indices (all data) | R1 = 0.0402, wR2 = 0.0953 | |
| Extinction coefficient | 0.0085(6) | |
| Largest diff. peak and hole | 0.328 and -0.302 e.$Å^{-3}$ | |

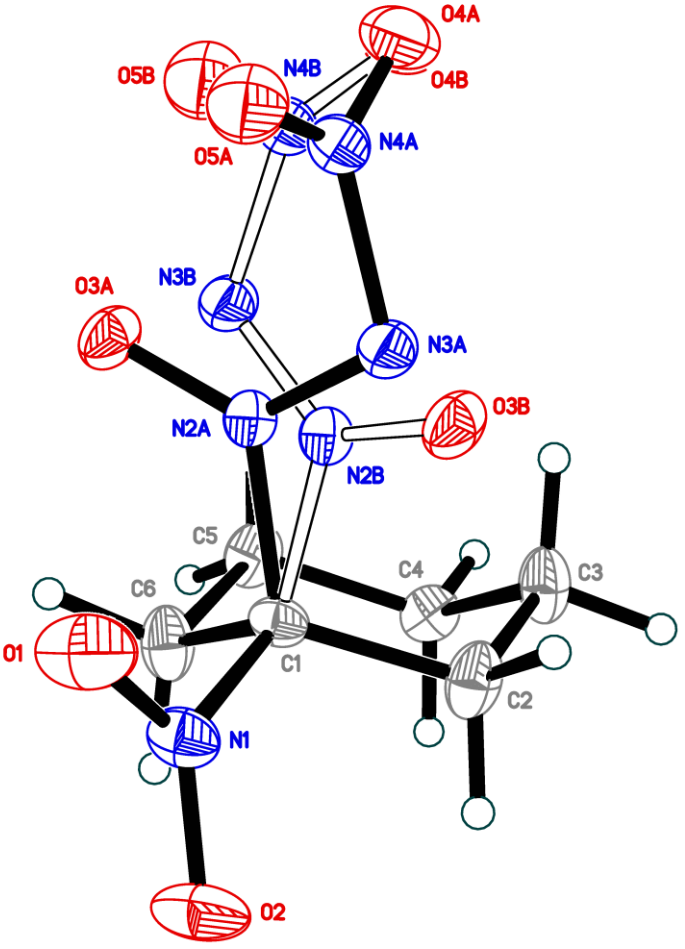


**Figure S6.** Crystal structure of compound **2c**, showing the atomic numbering and 50% probability displacement ellipsoids. N(O)=N–$NO_2$ group is conformationally disordered in the crystal, which results in doubled number of corresponding atoms in the figure (N2A-N2B, O3A-O3B, N3A-N3B, N4A-N4B, O4A-O4B, O5A-O5B).

## 8. Computational details

Computations were performed for 1 atm. and 298.15 K in Orca 6.1.0 package [25]. Results of DFT calculations were visualized by Chemcraft 1.8 program. For conformationally flexible structures, generation of conformational ensembles was performed by GOAT algorithm [26] implemented in Orca: for closed shell species it was made using GFN2-xTB method [27] and for open-shell species by native ORCA 6.1.0 spin-polarized variant of GFN2-xTB method (“Native-spGFN2-xTB” keyword). ALPB(MeCN) solvation model [28] was used in both cases. Bond length constraints were used for the generation of conformer ensembles of transition states in order to avoid optimization to starting reagent(s) or product(s). On the next step, most stable conformers were identified by re-optimization of generated conformers and vibrational analysis on

ωB97X-3c [29]/CPCM(MeCN) level of theory (in the case of >15 conformers, 15 most stable according to GFN2-xTB were analyzed). Results are shown for the most stable conformations of each structure. Optimized XYZ geometries and energies (ωB97X-3c/CPCM(MeCN)) used for Figure 3 and Scheme 5 in the manuscript are listed below.

**1a**

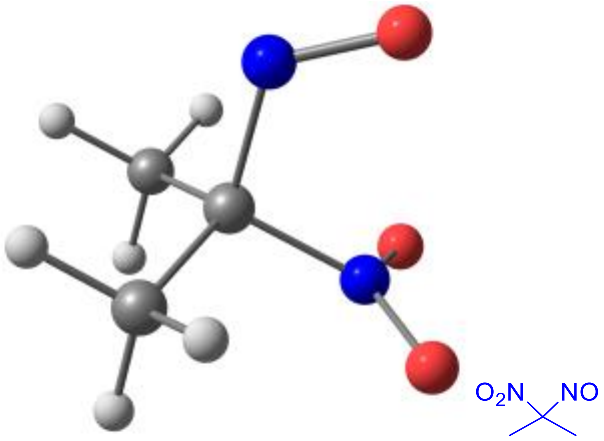


Multiplicity = 1
Charge = 0
Imaginary vib. modes: no

| Electronic energy | Total Enthalpy (H) | Final Gibbs free energy (G) |
|---|---|---|
| -88.61973829 | -88.50485553 | -88.54661568 |

| | | | |
|---|---|---|---|
| C | -1.19129322145273 | -0.61009005442990 | 0.05120785288206 |
| C | -2.67621117879387 | -0.41167350674600 | 0.23322463219885 |
| H | -2.99600240002531 | -1.00984906074895 | 1.08182767778105 |
| H | -2.91840128944929 | 0.63047414865051 | 0.41649832440944 |
| H | -3.19694382300988 | -0.75513829112938 | -0.65702758367283 |
| C | -0.78378577840415 | -2.05537178111373 | -0.09971023673902 |
| H | 0.29550143713530 | -2.16139412526478 | -0.14638899424826 |
| H | -1.16271762919917 | -2.60542314436506 | 0.75716444717876 |
| H | -1.22878929752848 | -2.46197292759888 | -1.00438769917832 |
| N | -0.68170131630673 | 0.21362780490640 | -1.11997088898252 |
| O | 0.33104938535972 | -0.15608118185465 | -1.67322037979945 |
| O | -1.27781360222323 | 1.23165196086189 | -1.39675387356176 |
| N | -0.53253661465540 | -0.10164603988860 | 1.28733084575277 |
| O | 0.22984373855321 | 0.80220955872115 | 1.14732717597923 |

**A**

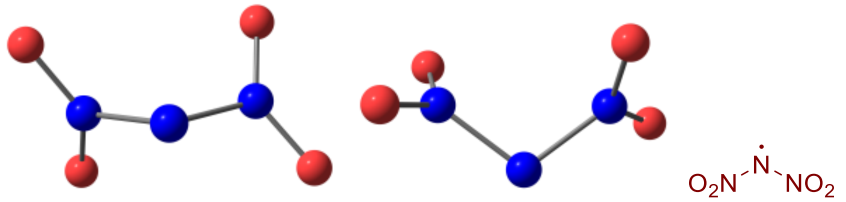


Multiplicity = 2

Charge = 0

Imaginary vib. modes: no

| Electronic energy | Total Enthalpy (H) | Final Gibbs free energy (G) |
|---|---|---|
| -93.75012150 | -93.71504556 | -93.75389593 |

| | | | |
|---|---|---|---|
| N | -0.00606819537664 | -0.26933454264553 | 0.86751553270836 |
| N | -0.05973449530991 | 1.08761263286963 | 0.39901821039298 |
| O | 0.83714493464841 | 1.76880687008774 | 0.80153816774391 |
| O | -1.00729587686324 | 1.38457894151598 | -0.27210027548133 |
| N | 0.06001119285543 | -1.10964532593888 | -0.29572305557585 |
| O | -0.89954362438526 | -1.80749465970382 | -0.44336058734055 |
| O | 1.07548606443121 | -1.05452391618513 | -0.92927299244752 |

**B**

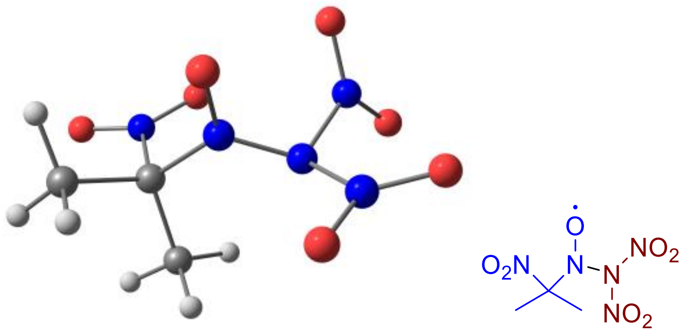


Multiplicity = 2

Charge = 0

Imaginary vib. modes: no

| Electronic energy | Total Enthalpy (H) | Final Gibbs free energy (G) |
|---|---|---|
| -182.42036273 | -182.26591099 | -182.32237140 |

| | | | |
|---|---|---|---|
| N | 1.73137185724121 | 0.11995693550806 | -1.50606975650747 |
| O | 1.11051687766105 | -0.53134101106116 | -2.29184541768039 |
| O | 2.84209854598572 | 0.55540583375071 | -1.58828267779426 |
| N | 1.91913558692369 | 1.01483920218157 | 0.73478788019692 |
| O | 2.28181274974560 | 0.26100904503028 | 1.58361492201903 |
| O | 2.15320321530173 | 2.17586943229748 | 0.59254214134411 |
| N | 0.98448322379849 | 0.47453845909569 | -0.30608322610918 |
| N | 0.18688976640805 | -0.54193797344671 | 0.12837527562215 |

| | | | |
|---|---|---|---|
| O | 0.68950620412506 | -1.66803940087715 | 0.37185893722781 |
| C | -1.25379012366840 | -0.28968108517291 | 0.26607057208346 |
| C | -2.01155951366693 | -1.57549894734686 | 0.06285282294858 |
| H | -1.74063902319611 | -2.31974609866633 | 0.80421661972023 |
| H | -3.07488654489057 | -1.37195490044274 | 0.12107622786569 |
| H | -1.77475792021574 | -1.94999605600303 | -0.92960310054552 |
| C | -1.71956658412796 | 0.83074438589098 | -0.63485380907107 |
| H | -1.54963059965552 | 0.54064166909016 | -1.66841500984107 |
| H | -2.78578627225440 | 0.97022903770222 | -0.48000007905342 |
| H | -1.19923844965693 | 1.76047846347073 | -0.42760632072732 |
| N | -1.45368551266440 | 0.21370828685848 | 1.70713977542094 |
| O | -2.52061307732024 | 0.00428299930798 | 2.22943113646332 |
| O | -0.54497440587343 | 0.84299872283251 | 2.20483008641739 |

**TS1**

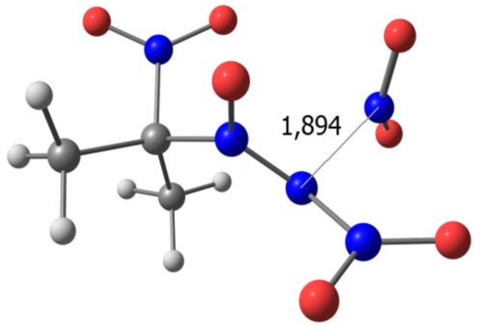


Multiplicity = 2

Charge = 0

Imaginary vib. modes: 1

| Electronic energy | Total Enthalpy (H) | Final Gibbs free energy (G) |
|---|---|---|
| -182.40988257 | -182.25731544 | -182.31363083 |

| | | | |
|---|---|---|---|
| N | 1.87683017793205 | -0.24716517422320 | -1.43716134479860 |
| O | 1.52486568759124 | -1.18153479085251 | -2.10194755168247 |
| O | 2.93744983158976 | 0.31612804050464 | -1.45634438766719 |
| N | 1.89129536591517 | 1.34962271237227 | 0.62649000301437 |
| O | 2.40682015300437 | 0.69184995891453 | 1.45983000130675 |
| O | 1.79643844672114 | 2.50708324125062 | 0.42960585481925 |
| N | 0.85915708196828 | 0.35412178349185 | -0.61094874364923 |
| N | 0.15511237731351 | -0.55174279531731 | 0.03496977007237 |
| O | 0.65890616854076 | -1.58782509125407 | 0.51316341643578 |
| C | -1.28641983882163 | -0.27618629064635 | 0.25661342770449 |

| | | | |
|---|---|---|---|
| C | -2.07266232885766 | -1.51356174183267 | -0.08781031491367 |
| H | -1.77623431984193 | -2.35600029664745 | 0.52835165364453 |
| H | -3.12968981974825 | -1.31287978285047 | 0.04875921002679 |
| H | -1.88840459157623 | -1.74524700343201 | -1.13398757907969 |
| C | -1.75549277498993 | 0.95919741291490 | -0.47164974487326 |
| H | -1.65074844884680 | 0.79779520094039 | -1.54055008635463 |
| H | -2.80660806971626 | 1.10518540507948 | -0.23743250462509 |
| H | -1.19321908788945 | 1.84091593534444 | -0.18041419383612 |
| N | -1.39432280107234 | 0.02957012524184 | 1.75459533303794 |
| O | -2.40940054553786 | -0.29582878663640 | 2.31871182580226 |
| O | -0.47378366367794 | 0.63300893763747 | 2.26119495561544 |

**2a**

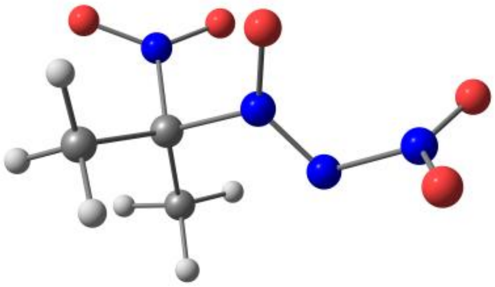

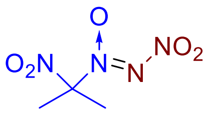


Multiplicity = 1
Charge = 0
Imaginary vib. modes: no

| Electronic energy | Total Enthalpy (H) | Final Gibbs free energy (G) |
|---|---|---|
| -140.50615752 | -140.36702301 | -140.41632533 |

| | | | |
|---|---|---|---|
| C | -1.38503001641060 | -0.49231259563713 | -0.38816591715910 |
| C | -2.66033556153732 | -0.51994049397800 | -1.18278963023953 |
| H | -2.59506132134316 | -1.30912711623867 | -1.92428229012610 |
| H | -3.47599792319491 | -0.74508838514962 | -0.49948720918475 |
| H | -2.84379091728719 | 0.43167675304065 | -1.67077266580716 |
| C | -0.98858318756207 | -1.81815112890264 | 0.20380602547491 |
| H | -1.80675913658589 | -2.20184152180558 | 0.80503013218299 |
| H | -0.80793336944865 | -2.50360605483201 | -0.62068167300729 |
| H | -0.09211333775872 | -1.73269615059170 | 0.80966012320549 |
| N | -0.20940979193871 | -0.00685567347895 | -1.23166746539515 |
| O | 0.75000201165434 | 0.42471923136080 | -0.63347184499737 |
| O | -0.28946899623036 | -0.14462818484735 | -2.42708144424399 |

| | | | |
|---|---|---|---|
| N | -1.52037943859148 | 0.60133755031151 | 0.64962704763117 |
| O | -1.68791231502299 | 1.74576582058886 | 0.24890100354674 |
| N | -1.49317112095713 | 0.21460116638269 | 1.84465314340291 |
| N | -1.70550892262127 | 1.33538285112404 | 2.71540338849118 |
| O | -0.71315125803792 | 1.88103437957937 | 3.11394550687559 |
| O | -2.84694326712596 | 1.55573933307374 | 3.01613262934944 |

**C-*t***

| | | |
|---|---|---|
| 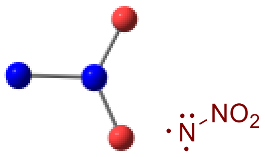 | | Multiplicity = 3<br>Charge = 0<br>Imaginary vib. modes: no |
| Electronic energy | Total Enthalpy (H) | Final Gibbs free energy (G) |
| -51.77422133 | -51.75658253 | -51.78685597 |

| | | | |
|---|---|---|---|
| N | 0.00025602663687 | -1.34273892479182 | 0.00000495850803 |
| N | -0.00000704933274 | 0.04241666183309 | -0.00001791989415 |
| O | 1.08739579005668 | 0.56883978970590 | 0.00001098095759 |
| O | -1.08761276736081 | 0.56842447325283 | 0.00001098042853 |

**C-*s***

| | | |
|---|---|---|
| 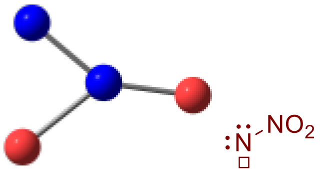 | | Multiplicity = 1<br>Charge = 0<br>Imaginary vib. modes: no |
| Electronic energy | Total Enthalpy (H) | Final Gibbs free energy (G) |
| -51.76678402 | -51.74873338 | -51.77847047 |

| | | | |
|---|---|---|---|
| N | 0.74176342423385 | -0.87149614279014 | 0.00000328647889 |
| N | -0.19138627028019 | -0.08403008259820 | -0.00000859325762 |
| O | 0.87853610928075 | 0.74966887124512 | 0.00001553414693 |
| O | -1.36076526323442 | 0.08627935414321 | 0.00003177263179 |

**TS2**

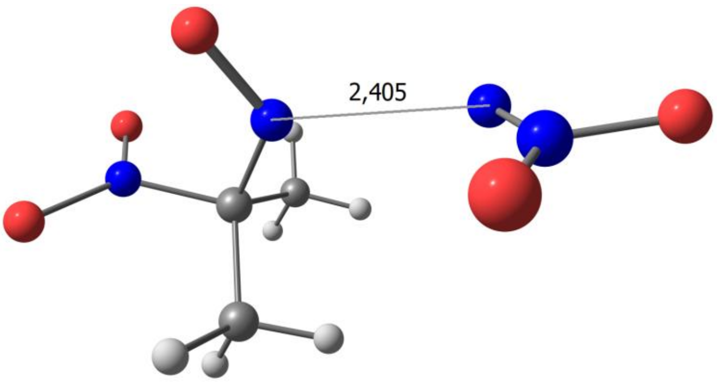


Multiplicity = 1
Charge = 0
Imaginary vib. modes: 1

| Electronic energy | Total Enthalpy (H) | Final Gibbs free energy (G) |
|---|---|---|
| -140.38895276 | -140.25493056 | -140.30778492 |

| | | | |
|---|---|---|---|
| C | 0.31962525585732 | -0.26437103175042 | -0.67537599081132 |
| C | -1.16608775292139 | -0.26675230050281 | -0.94078968922659 |
| H | -1.39639797235153 | -1.04883408461221 | -1.65989796656778 |
| H | -1.67734280439526 | -0.48345422985727 | -0.00668657254478 |
| H | -1.50330512957779 | 0.69135277596937 | -1.32311360392892 |
| C | 0.83303388789927 | -1.54378613387032 | -0.06143448437110 |
| H | 0.25116082399280 | -1.75072768291042 | 0.83212888241680 |
| H | 0.69300838078744 | -2.35640861494501 | -0.76975836080547 |
| H | 1.88340607304876 | -1.46663350402257 | 0.20072927343250 |
| N | 1.09470741681229 | 0.06906970790462 | -1.94349893884870 |
| O | 2.24413039250202 | -0.30596525834013 | -2.01285569842533 |
| O | 0.53847583811205 | 0.73608478838533 | -2.78720577752356 |
| N | 0.57866016955343 | 0.82513848292494 | 0.30312757654439 |
| O | 1.27739767460198 | 1.71912881598935 | -0.03771726370370 |
| N | -0.11034365350436 | 0.62171694849986 | 2.59834236261677 |
| N | -1.03911996827621 | 1.44442661724675 | 2.58347601859999 |
| O | -1.20322484882398 | 1.19846413697098 | 3.85218173574512 |
| O | -1.61778378331685 | 2.18155056691996 | 1.84834849740168 |

**TS3**

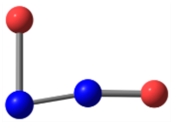

Multiplicity = 1
Charge = 0
Imaginary vib. modes: 1

| Electronic energy | Total Enthalpy (H) | Final Gibbs free energy (G) |
|---|---|---|
| -51.75571900 | -51.73942283 | -51.76933129 |

| | | | |
|---|---|---|---|
| N | 0.73671226008429 | -0.69304049133530 | -0.00000220919339 |
| N | -0.33904307603707 | -0.21263897852250 | 0.00001130703433 |
| O | 1.13333997627833 | 0.72136392948132 | 0.00000948015708 |
| O | -1.46286116032555 | 0.06473654037648 | 0.00002342200199 |

**$N_2O_2$**

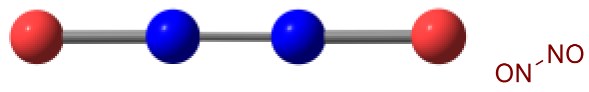

Multiplicity = 1
Charge = 0
Imaginary vib. modes: no

| Electronic energy | Total Enthalpy (H) | Final Gibbs free energy (G) |
|---|---|---|
| -51.81928374 | -51.80064364 | -51.82828974 |

| | | | |
|---|---|---|---|
| N | 2.14415841591455 | 0.52737577653439 | -0.00441271664201 |
| O | 3.22208688301196 | -0.03621331479552 | 0.00118991161053 |
| N | 1.16572504288145 | 1.04542215920154 | 0.00456917485830 |
| O | 0.08759279819202 | 1.60869949905958 | -0.00134566982682 |

**$NO_2$**

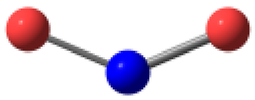

Multiplicity = 2
Charge = 0
Imaginary vib. modes: no

| Electronic energy | Total Enthalpy (H) | Final Gibbs free energy (G) |
|---|---|---|
| -41.93569372 | -41.92258985 | -41.94979874 |

| | | | |
|---|---|---|---|
| N | -0.00007590228973 | 0.32294151148970 | 0.00000000000000 |
| O | 1.09088287556731 | -0.14121461103750 | 0.00000000000000 |
| O | -1.09080697327758 | -0.14176190045220 | 0.00000000000000 |

## 9. References


(1) Klapötke, T. M.; Krumm, B.; Xaver Steemann, F.; Steinhauser, G. *Safety Science* **2010**, *48*, 28–34. doi:10.1016/j.ssci.2009.05.002

(2) Chen, F.; Xuan, C.; Lu, Q.; Xiao, L.; Yang, J.; Hu, Y.; Zhang, G.-P.; Wang, Y.; Zhao, F.; Hao, G.; Jiang, W. *Defence Technology* **2023**, *19*, 163–195. doi:10.1016/j.dt.2022.04.006

(3) Jeleva, N.; Gencheva, P. *ETR* **2025**, *4*, 133–137. doi:10.17770/etr2025vol4.8400

(4) Rahmani, M.; Soleimani, M.; Pouretedal, H. R.; Keshavarz, M. H. *Journal of Energetic Materials* **2025**, 1–22. doi:10.1080/07370652.2025.2529153

(5) Luk'yanov, O. A.; Gorelik, V. P.; Tartakovskii, V. A. *Russ. Chem. Bull.* **1994**, *43*, 89–92. doi:10.1007/BF00699142

(6) Li, X.; Deng, H.; Zhu, X.-Q.; Wang, X.; Liang, H.; Cheng, J.-P. *J. Org. Chem.* **2009**, *74*, 4472–4478. doi:10.1021/jo900732b

(7) Rehse, K.; Herpel, M. *Arch. Pharm. Pharm. Med. Chem.* **1998**, *331*, 79–84. doi:10.1002/(SICI)1521-4184(199802)331:2%3C79::AID-ARDP79%3E3.0.CO;2-9

(8) Nametkin, S. S. *Zhurnal Russkago Fiziko-Khimicheskago Obshchestva* **1910**, *42*, 585–586

(9) Charlton, W.; Earl, J. C.; Kenner, J.; Luciano, A. A. *J. Chem. Soc.* **1932**, 30. doi:10.1039/jr9320000030

(10) Luk'yanov, O. A.; Salamonov, Yu. B.; Bass, A. G.; Strelenko, Yu. A. *Russ. Chem. Bull.* **1991**, *40*, 93–98. doi:10.1007/BF00959638

(11) Ungnade, H. E.; Kissinger, L. W. *J. Org. Chem.* **1959**, *24*, 666–668. doi:10.1021/jo01087a026

(12) Budnikov, A. S.; Leonov, N. E.; Klenov, M. S.; Shevchenko, M. I.; Dvinyaninova, T. Y.; Krylov, I. B.; Churakov, A. M.; Fedyanin, I. V.; Tartakovsky, V. A.; Terent'ev, A. O. *Molecules* **2024**, *29*, 5563. doi:10.3390/molecules29235563

(13) Dhingra, O. D.; Sinclair, J. B. *Basic Plant Pathology Methods*, 4. pr.; CRC Press, Inc: Boca Raton; Flor, 1987

(14) Xu, H.; Fan, L. *Eur. J. Med. Chem.* **2011**, *46*, 364–369. doi:10.1016/j.ejmech.2010.10.022

(15) Singh, P. K. *J. Agric. Food Chem.* **2012**, *60*, 5813–5818. doi:10.1021/jf300730f

(16) Itoh, H.; Kajino, H.; Tsukiyama, T.; Tobitsuka, J.; Ohta, H.; Takahi, Y.; Tsuda, M.; Takeshiba, H. *Bioorg. Med. Chem.* **2002**, *10*, 4029–4034. doi:10.1016/S0968-0896(02)00302-4

(17) Popkov, S. V.; Kovalenko, L. V.; Bobylev, M. M.; Molchanov, O. Yu.; Krimer, M. Z.; Tashchi, V. P.; Putsykin, Y. G. *Pestic. Sci.* **1997**, *49*, 125–129. doi:10.1002/(SICI)1096-9063(199702)49:2%3C125::AID-PS506%3E3.0.CO;2-0

(18) Budnikov, A. S.; Lopat'eva, E. R.; Krylov, I. B.; Segida, O. O.; Lastovko, A. V.; Ilovaisky, A. I.; Nikishin, G. I.; Glinushkin, A. P.; Terent'ev, A. O. *J. Agric. Food Chem.* **2022**, *70*, 4572–4581. doi:10.1021/acs.jafc.1c07413

(19) Budnikov, A. S.; Krylov, I. B.; Shevchenko, M. I.; Segida, O. O.; Lastovko, A. V.; Alekseenko, A. L.; Ilovaisky, A. I.; Nikishin, G. I.; Terent'ev, A. O. *Molecules* **2023**, *28*, 7863. doi:10.3390/molecules28237863

(20) Shuvaev, A. D.; Zhilin, E. S.; Fershtat, L. L. *Synthesis* **2023**, *55*, 1863–1874. doi:10.1055/a-2011-7264

(21) CrysAlisPro. Version 1.171.41.106a. Rigaku Oxford Diffraction. 2021.

(22) Sheldrick, G. M. *Acta Crystallogr., Sect. A:Found. Adv.* **2015**, *71*, 3–8. doi:10.1107/S2053273314026370

(23) Sheldrick, G. M. *Acta Crystallogr., Sect. C:Struct. Chem.* **2015**, *71*, 3–8. doi:10.1107/S2053229614024218

(24) Dolomanov, O. V.; Bourhis, L. J.; Gildea, R. J.; Howard, J. A. K.; Puschmann, H. *J Appl Crystallogr* **2009**, *42*, 339–341. doi:10.1107/S0021889808042726

(25) Neese, F. *WIREs Comput. Mol. Sci.* **2025**, *15*, e70019. doi:10.1002/wcms.70019
(26) De Souza, B. *Angew Chem Int Ed* **2025**, *64*, e202500393. doi:10.1002/anie.202500393
(27) Bannwarth, C.; Ehlert, S.; Grimme, S. *J. Chem. Theory Comput.* **2019**, *15*, 1652–1671. doi:10.1021/acs.jctc.8b01176
(28) Ehlert, S.; Stahn, M.; Spicher, S.; Grimme, S. *J. Chem. Theory Comput.* **2021**, *17*, 4250–4261. doi:10.1021/acs.jctc.1c00471
(29) Müller, M.; Hansen, A.; Grimme, S. *J. Chem. Phys.* **2023**, *158*, 014103. doi:10.1063/5.0133026

## 10. NMR Data

### 10.1.1 $^1$H NMR spectrum of compound 2b [600.13 MHz, $CDCl_3$]

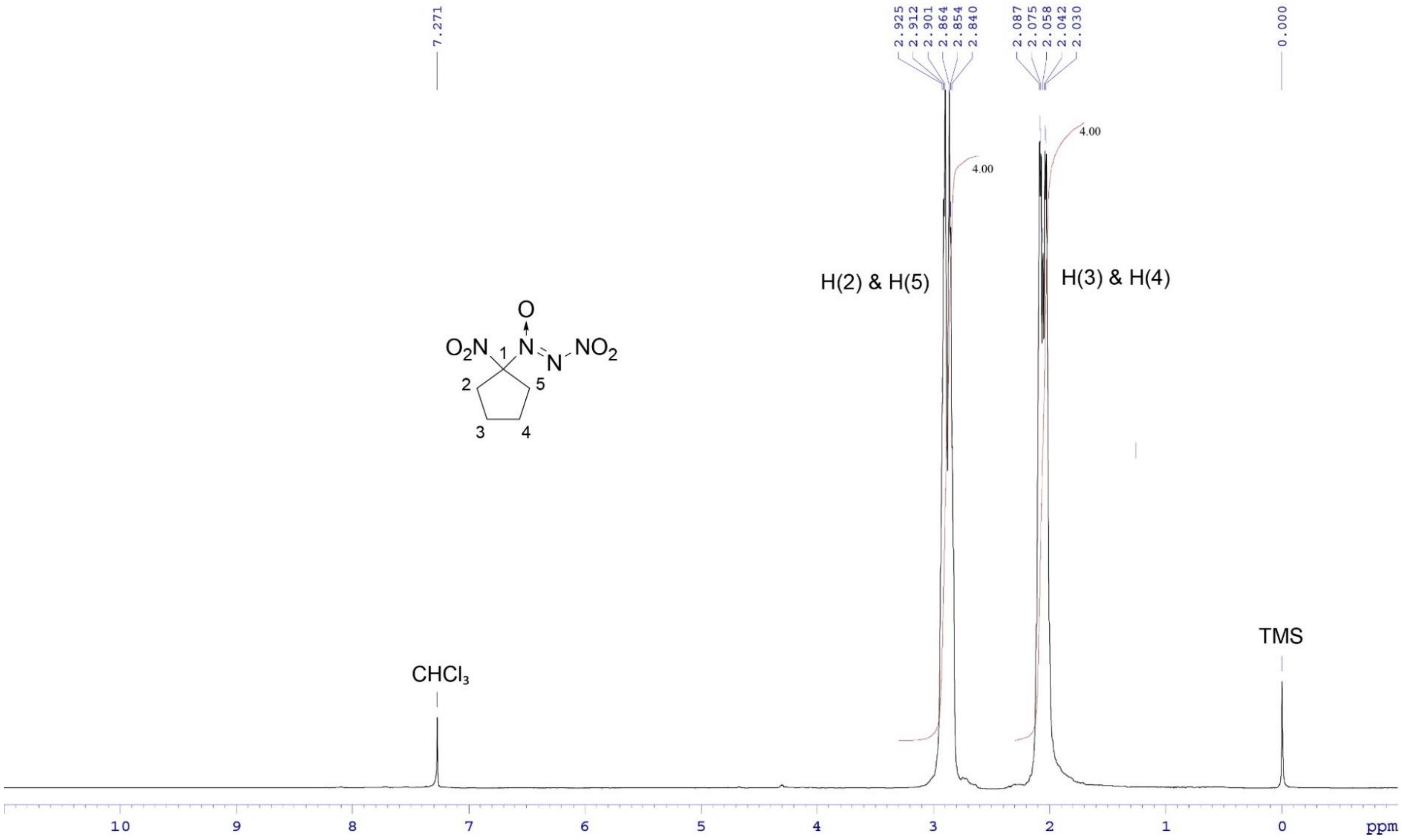

10.1.2 $^{13}$C NMR spectrum of compound 2b [150.90 MHz, $CDCl_3$]

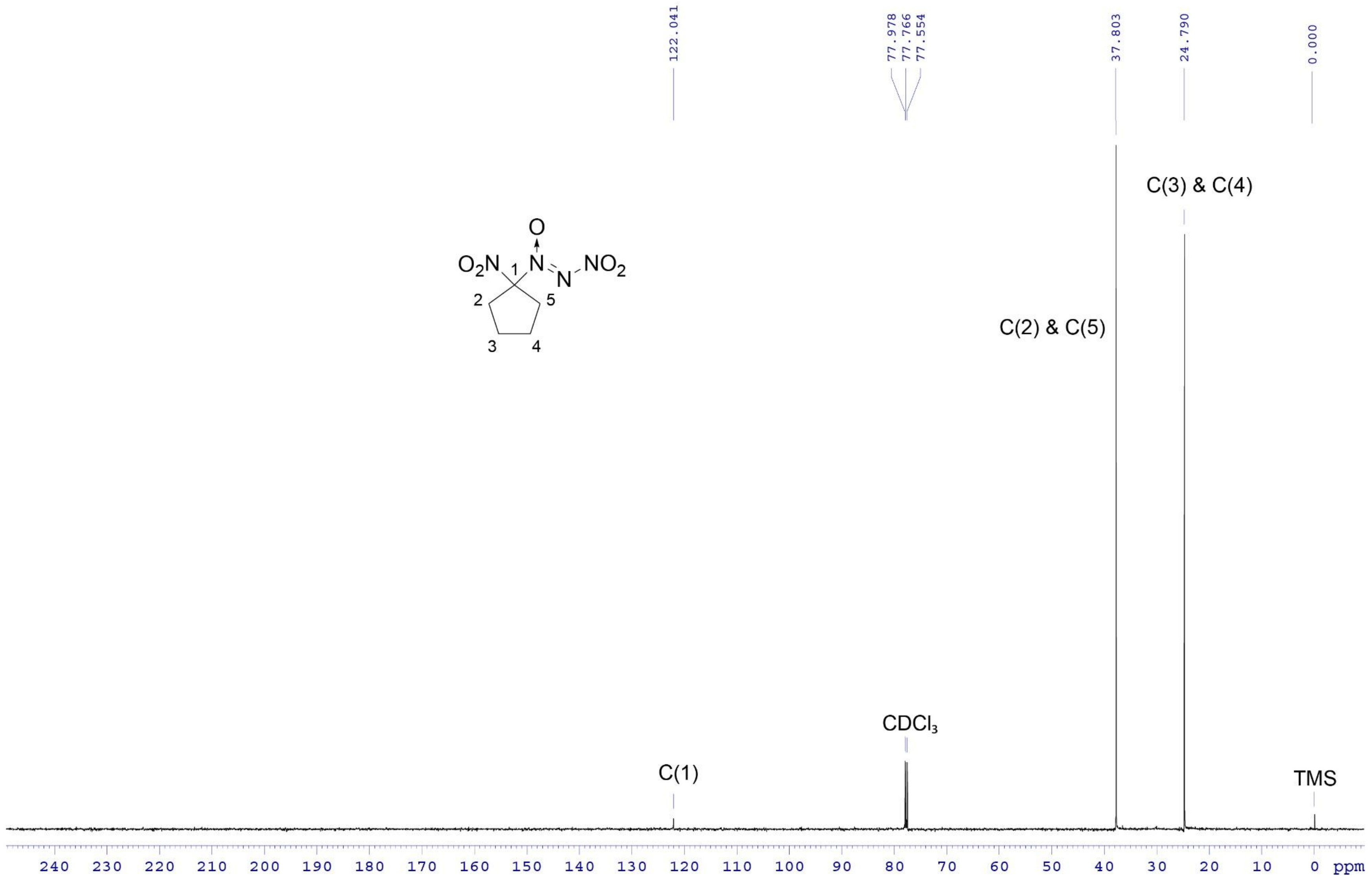

**10.1.3 {$^{1}H$–$^{13}C$} HSQC spectrum of compound 2b [600.13 MHz, $CDCl_3$]**

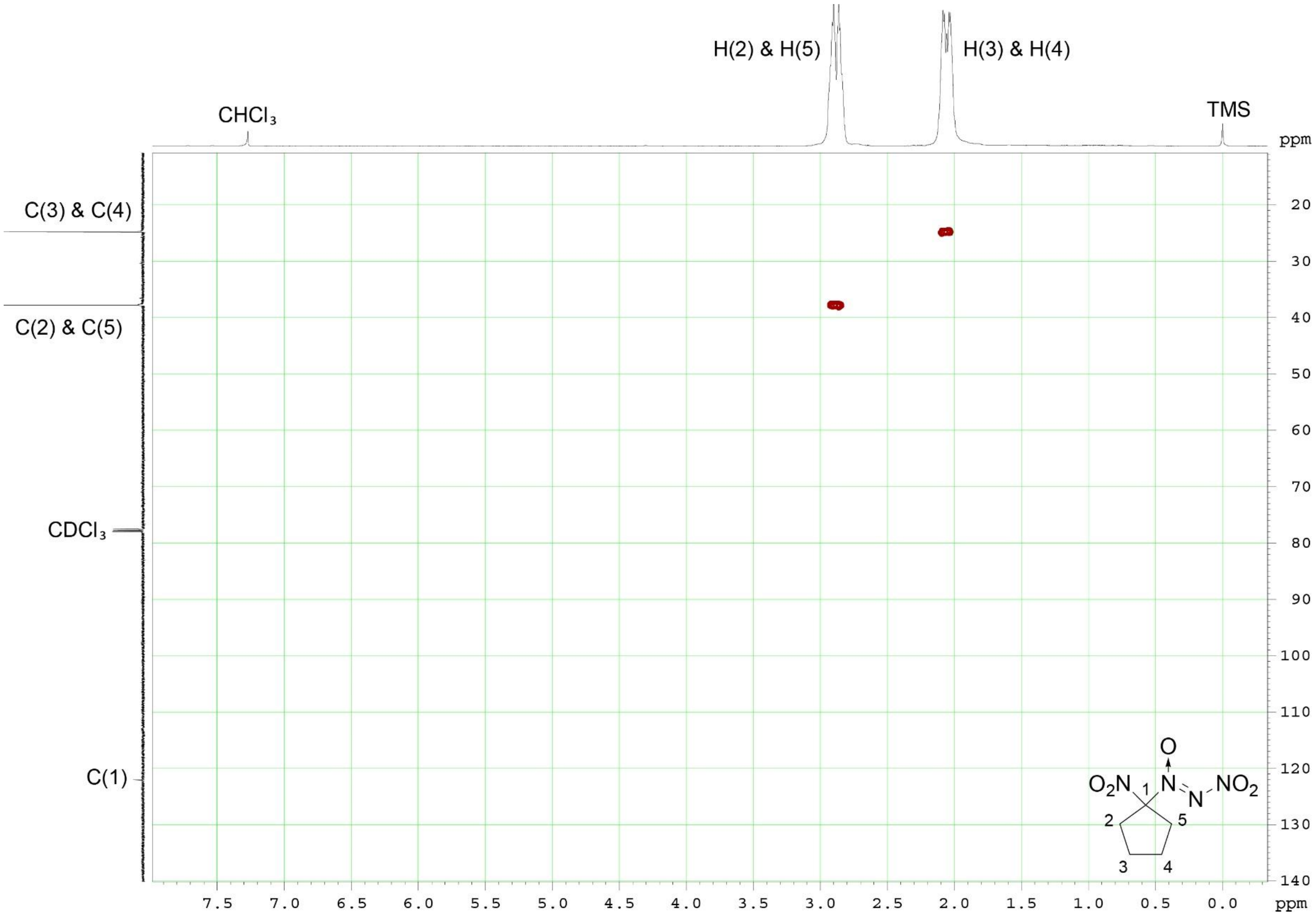

10.1.4 {$^1H$–$^{13}C$} HMBC spectrum of compound 2b [600.13 MHz, $CDCl_3$]

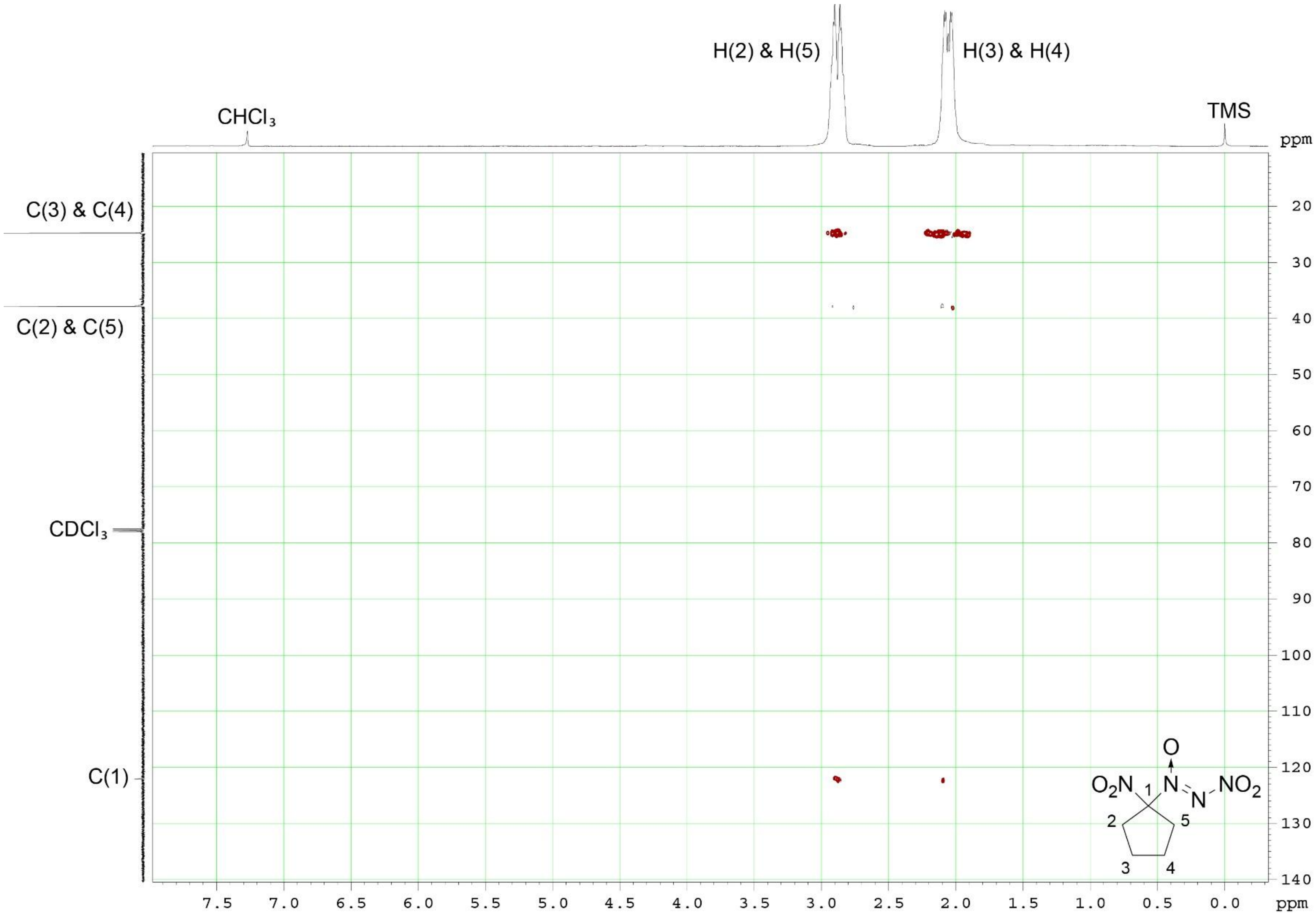

### 10.1.5 $^{14}N$ NMR spectrum of compound 2b [43.37 MHz, $CDCl_3$]

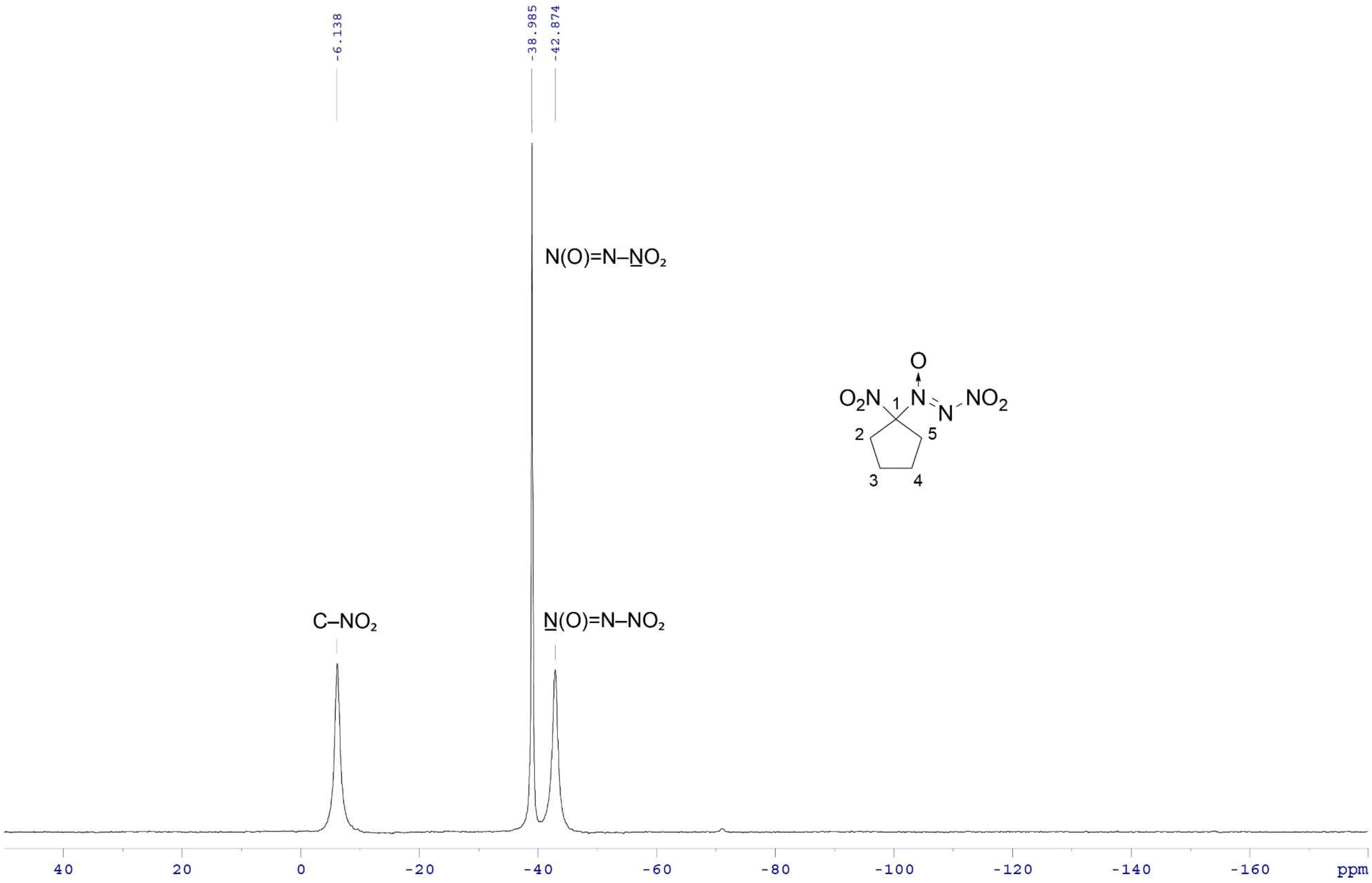

### 10.2.1 $^{1}$H NMR spectrum of compound 2c [500.13 MHz, $CDCl_3$]

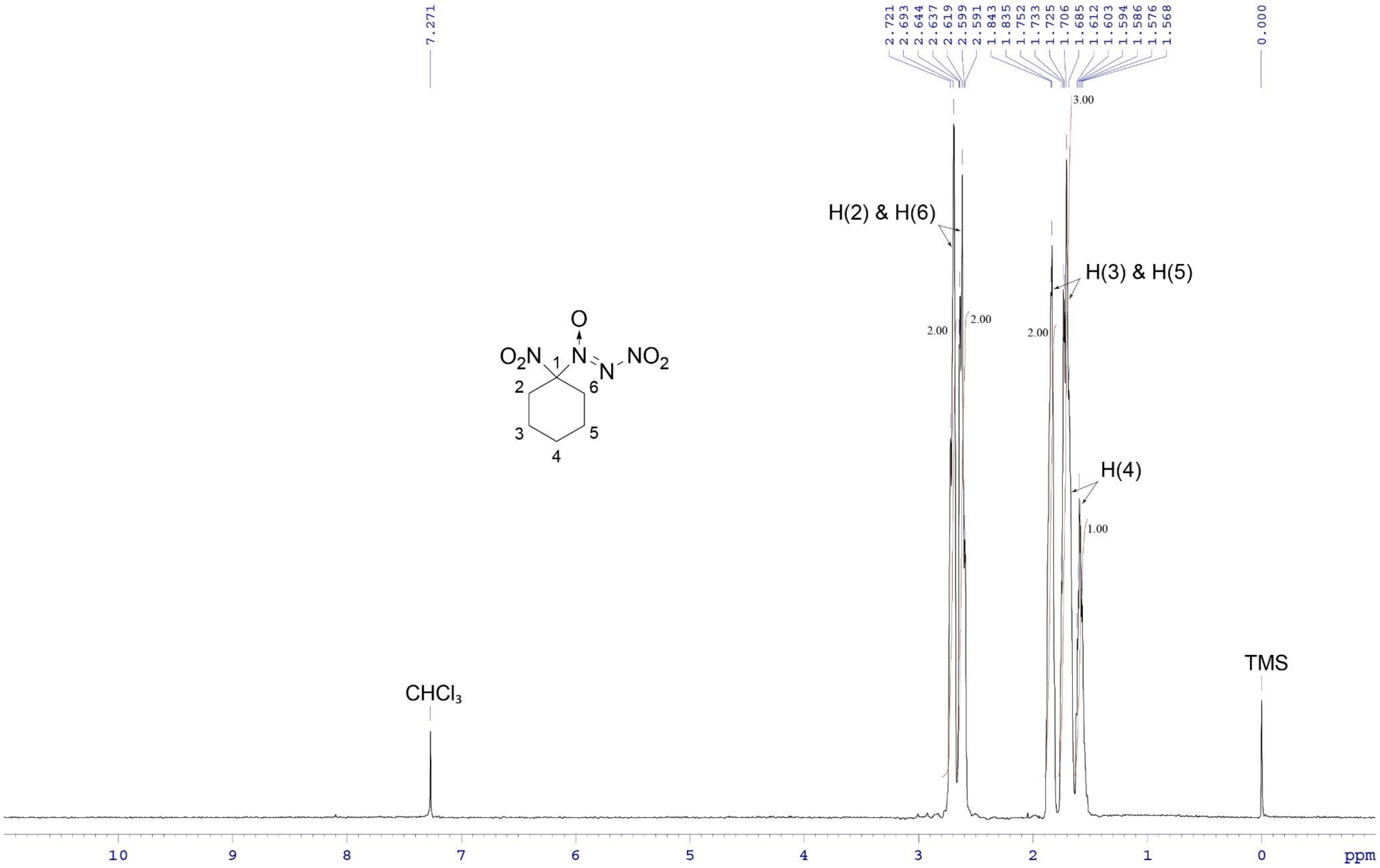

**10.2.2 $^{13}C$ NMR spectrum of compound 2c [125.76 MHz, $CDCl_3$]**

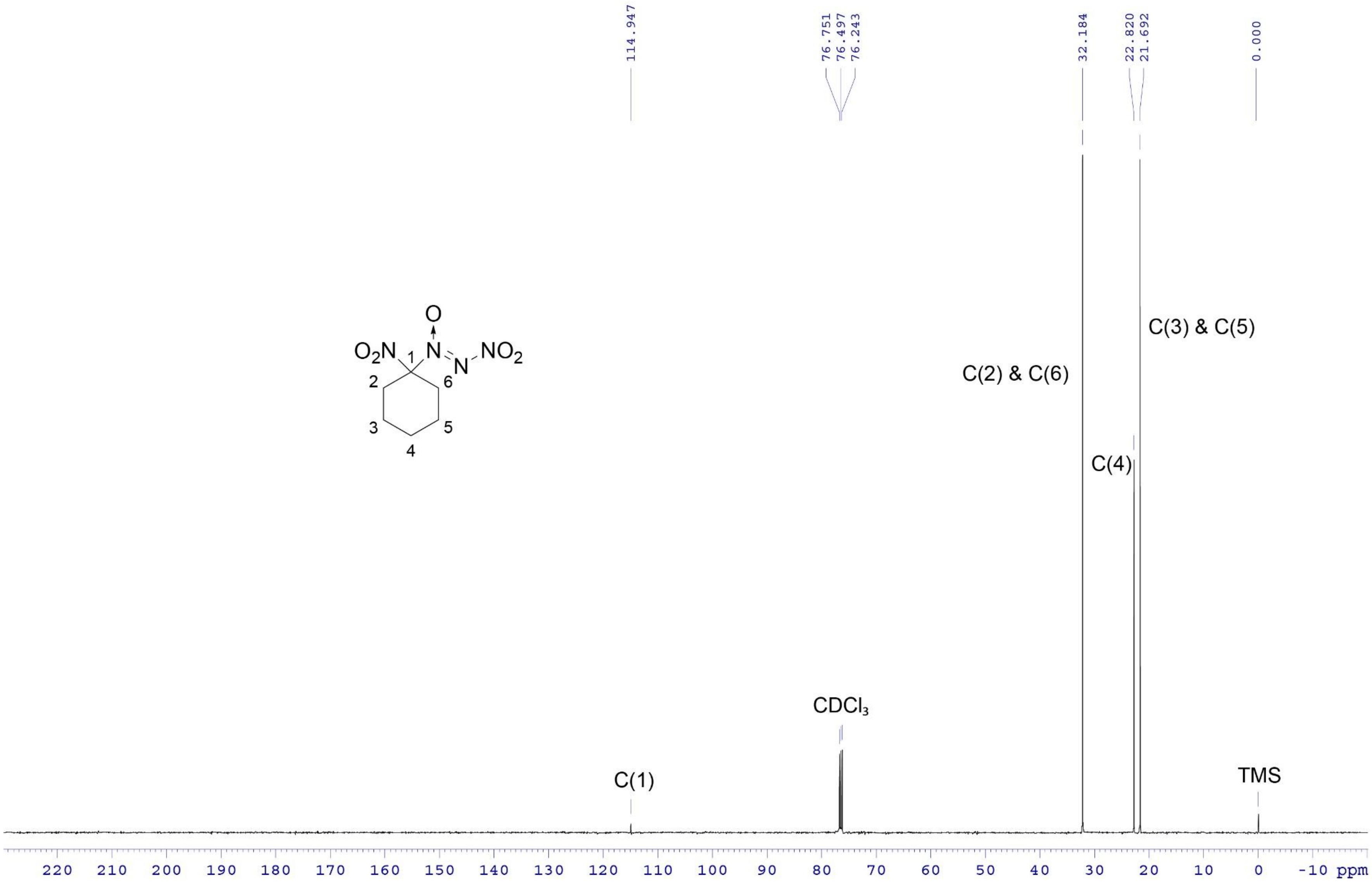

### 10.2.3 {$^1$H–$^{13}$C} HSQC spectrum of compound 2c [500.13 MHz, $CDCl_3$]

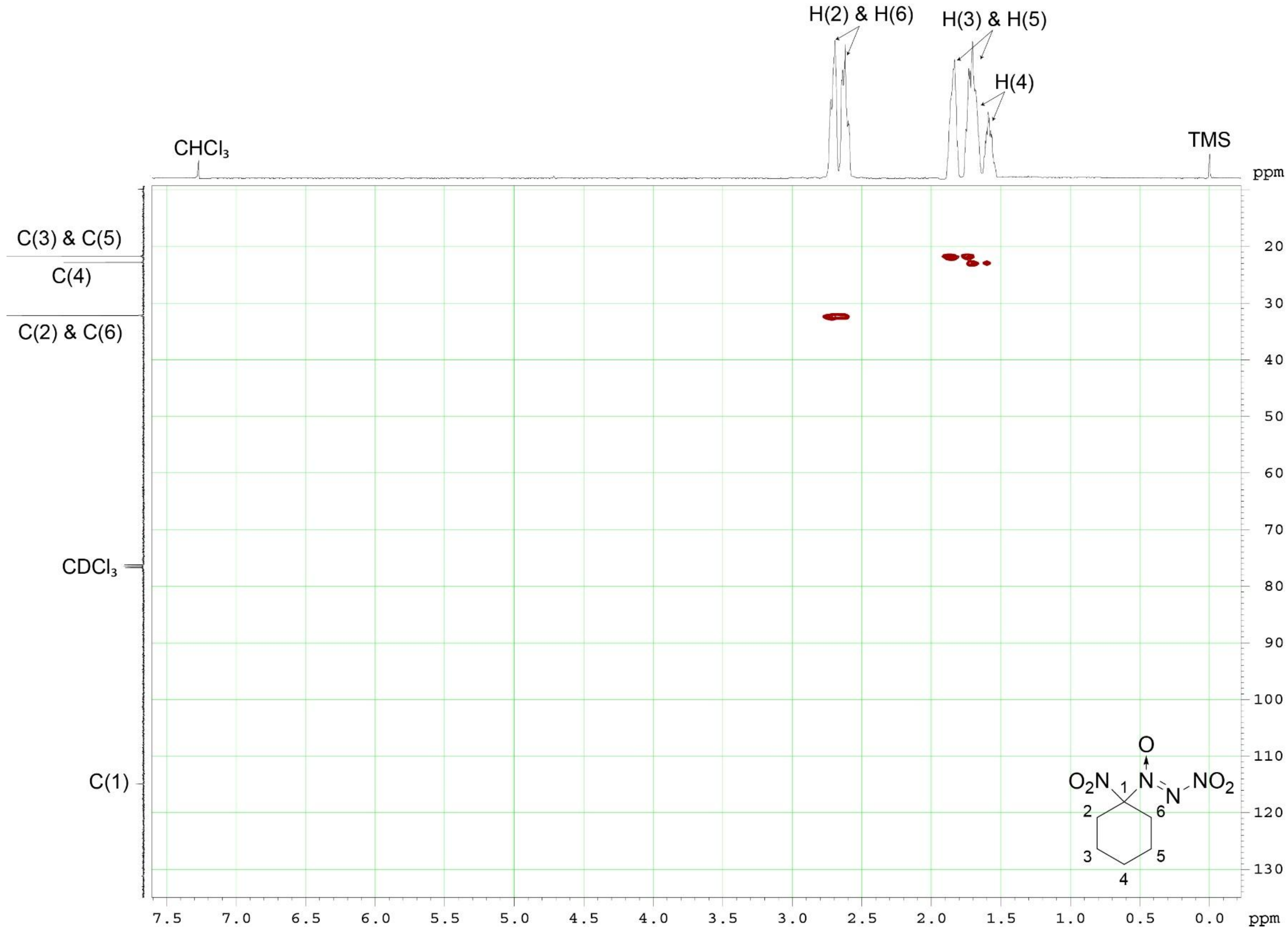

### 10.2.4 $^{14}N$ NMR spectrum of compound 2c [36.14 MHz, $CDCl_3$]

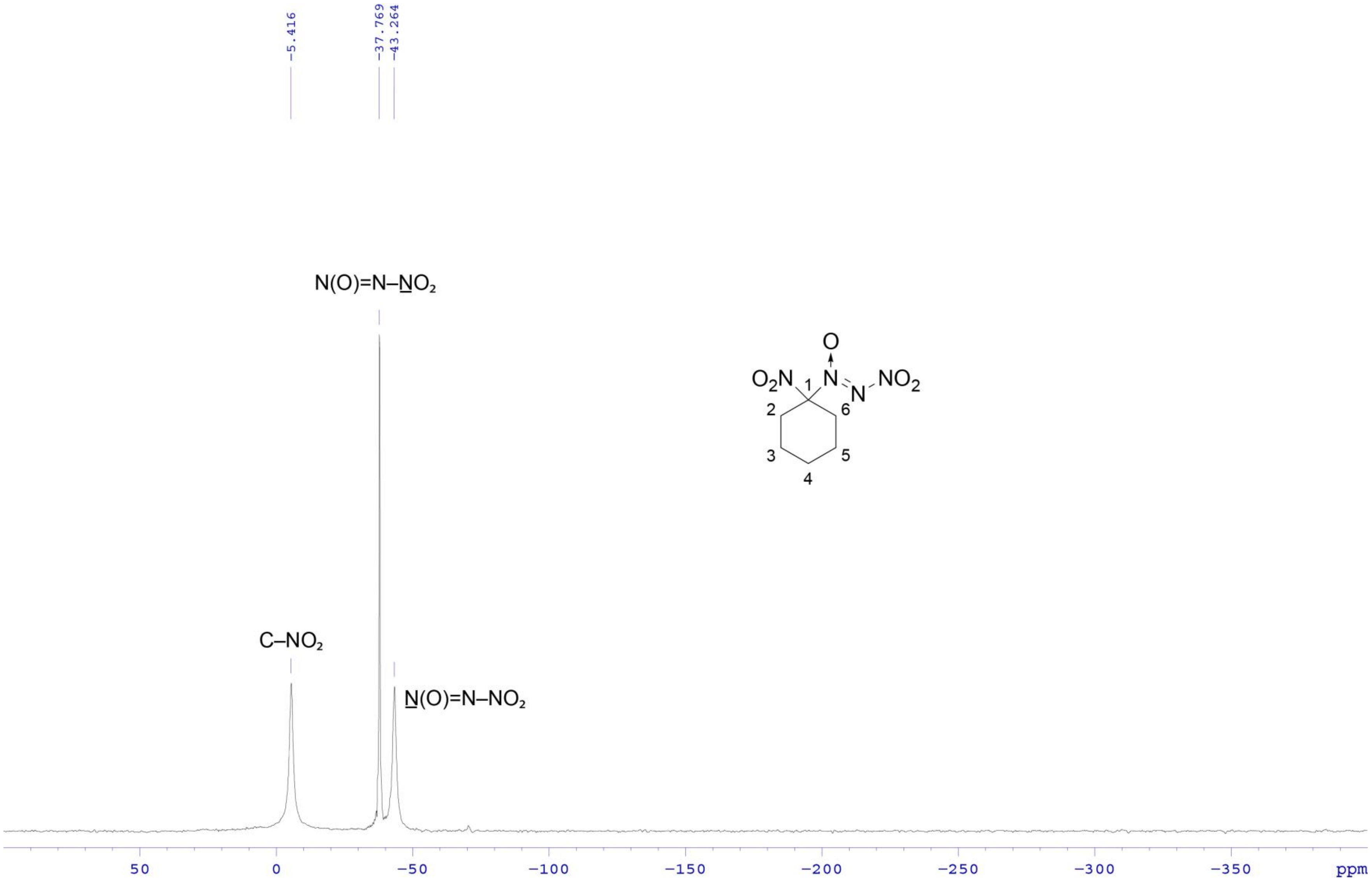

### 10.3.1 $^{1}$H NMR spectrum of compound 2d [500.13 MHz, $CDCl_3$]

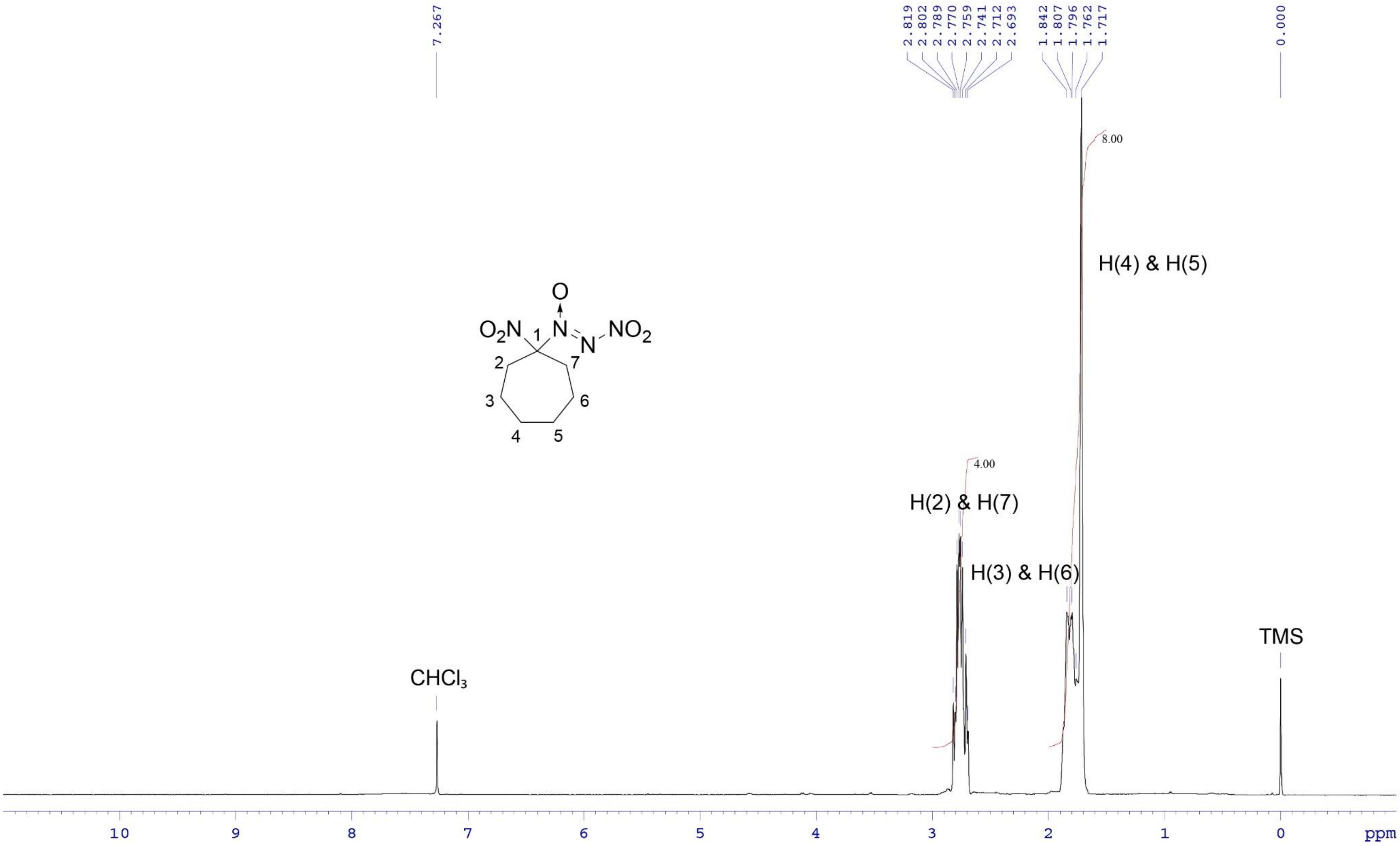

### 10.3.2 $^{13}C$ NMR spectrum of compound 2d [125.76 MHz, $CDCl_3$]

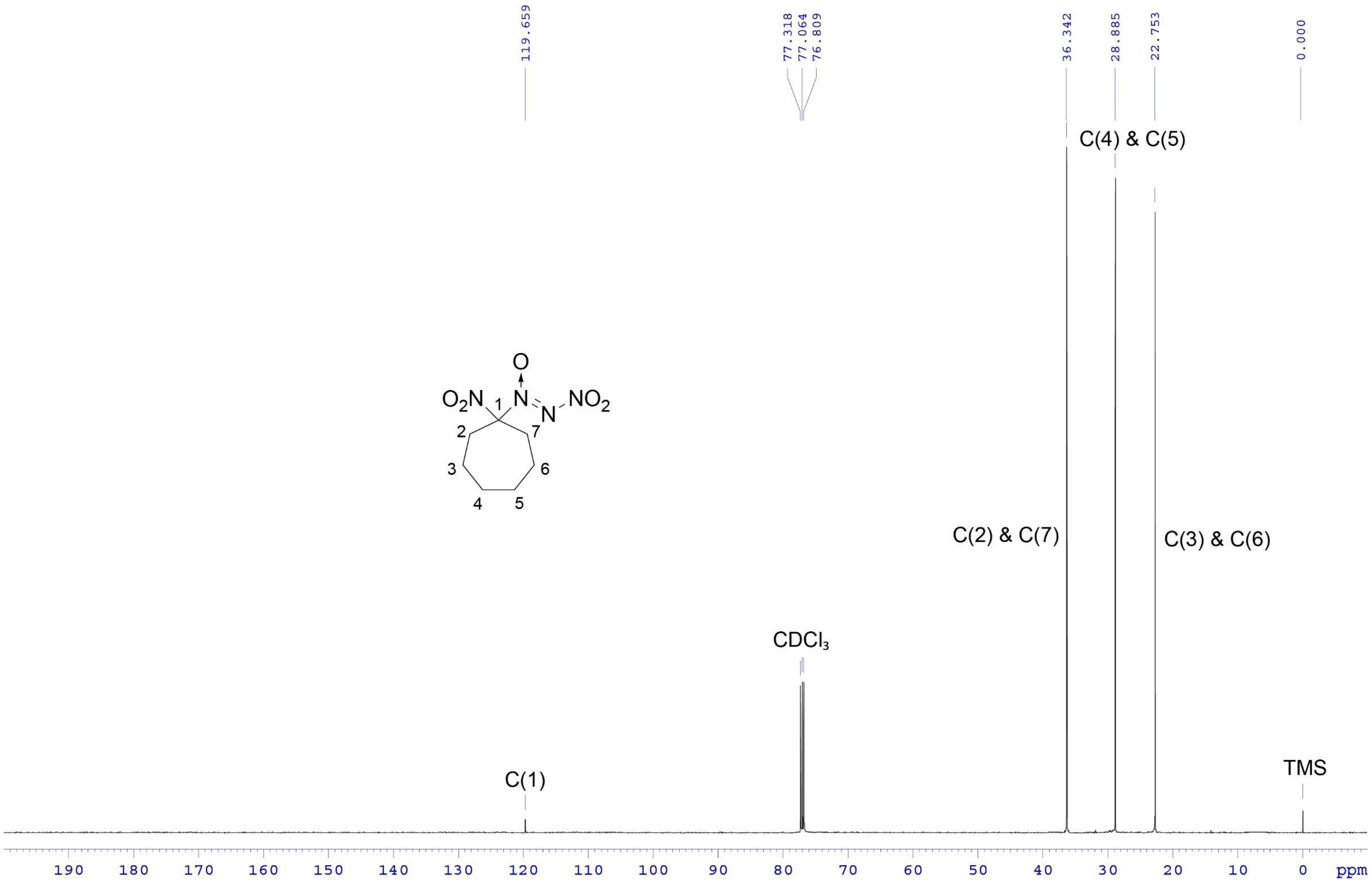

### 10.3.3 {$^1H$–$^{13}C$} HSQC spectrum of compound 2d [500.13 MHz, $CDCl_3$]

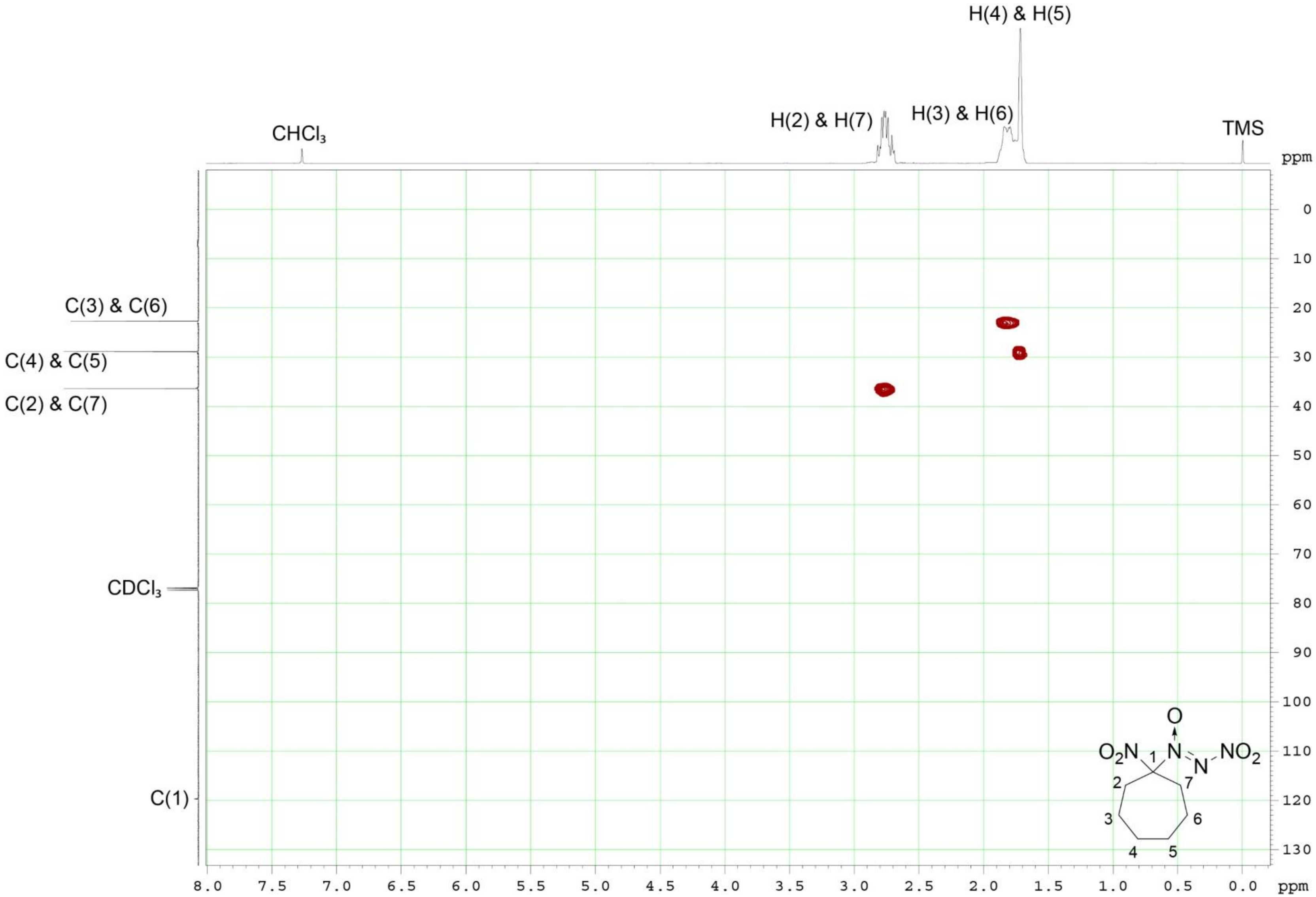

10.3.4 {$^1$H–$^{13}$C} HMBC spectrum of compound 2d [500.13 MHz, $CDCl_3$]

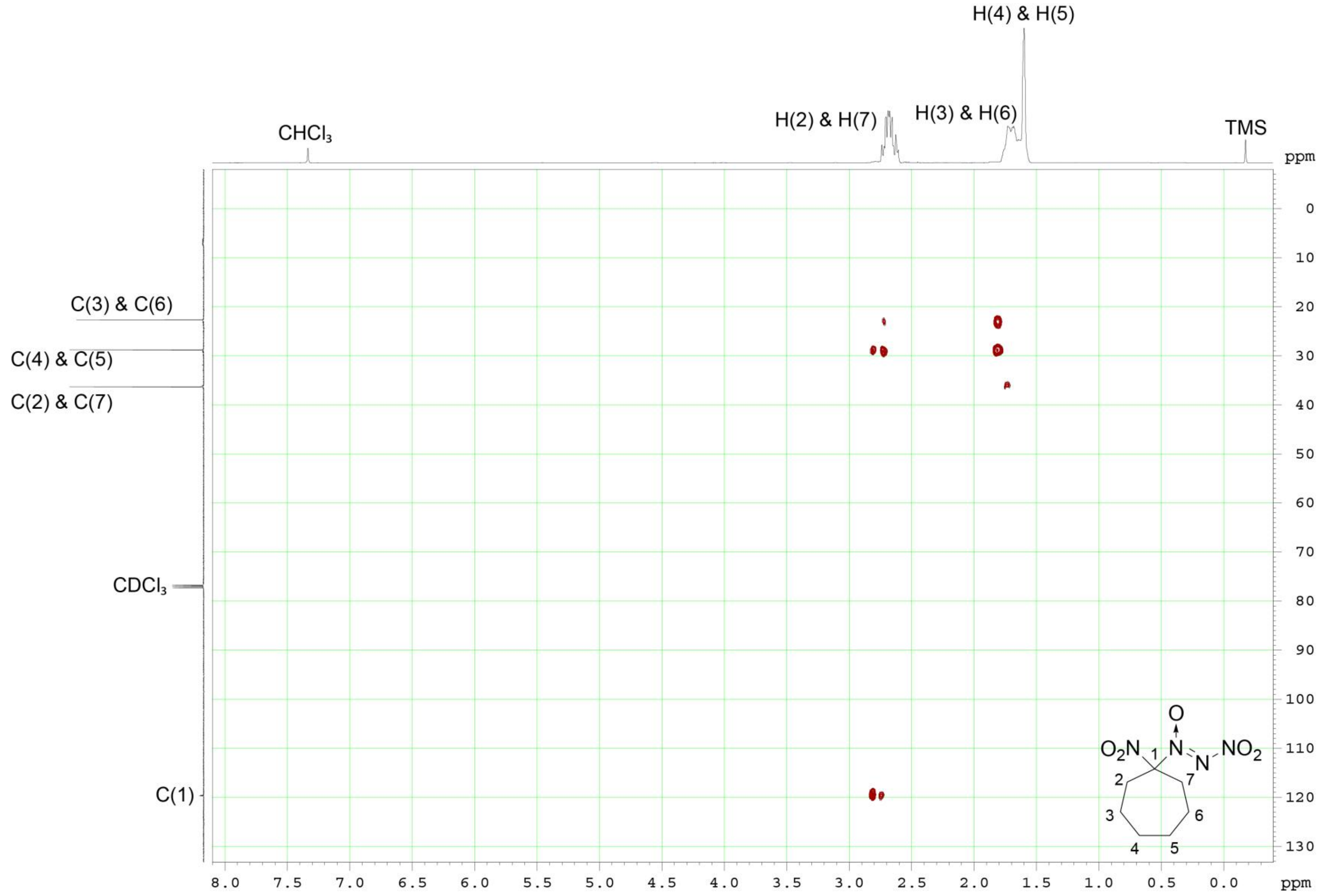

**10.3.5 $^{14}$N NMR spectrum of compound 2d [43.37 MHz, $CDCl_3$]**

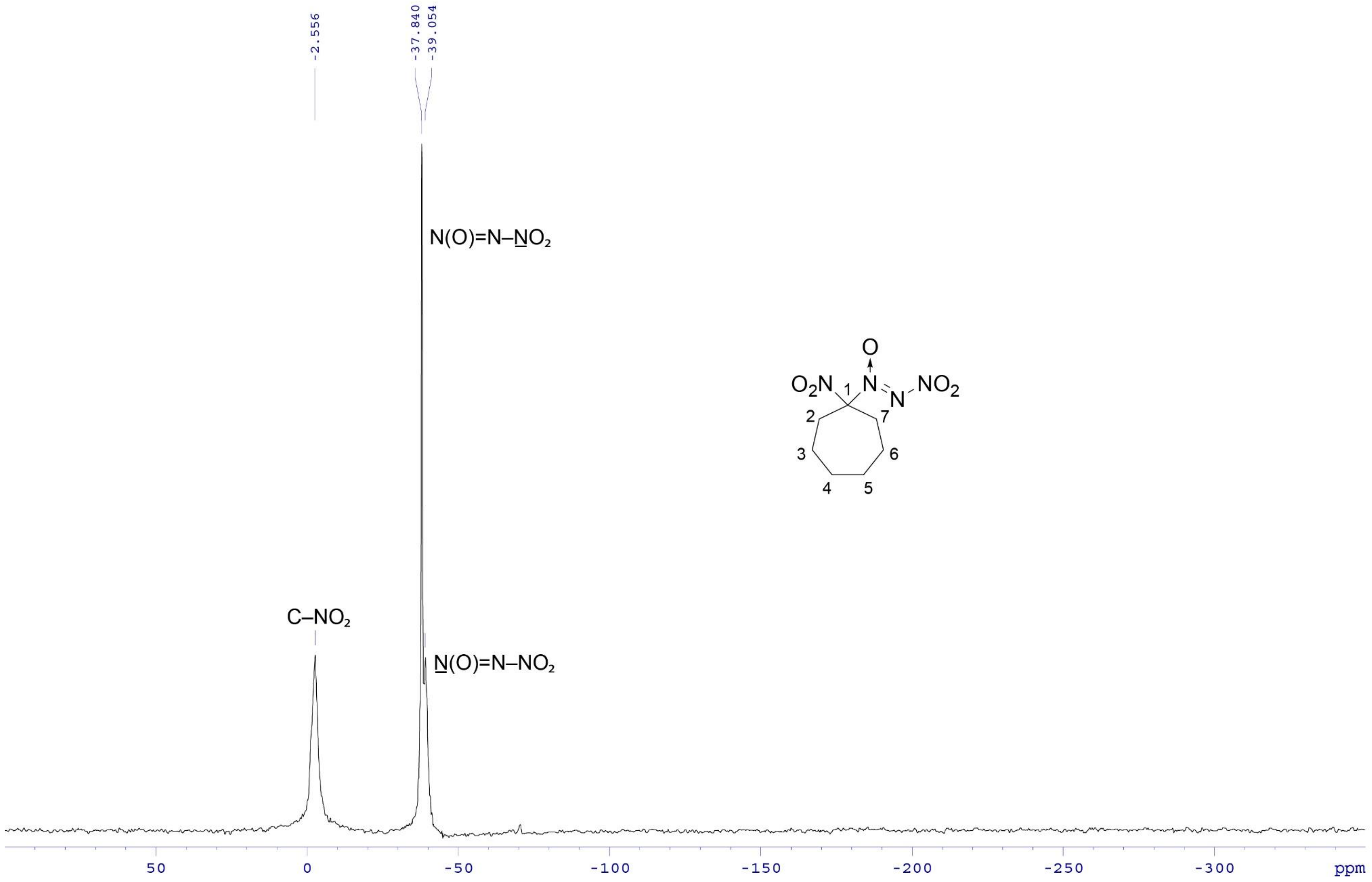

### 10.4.1 $^{1}$H NMR spectrum of compound 2e [500.13 MHz, [$D_6$]acetone]

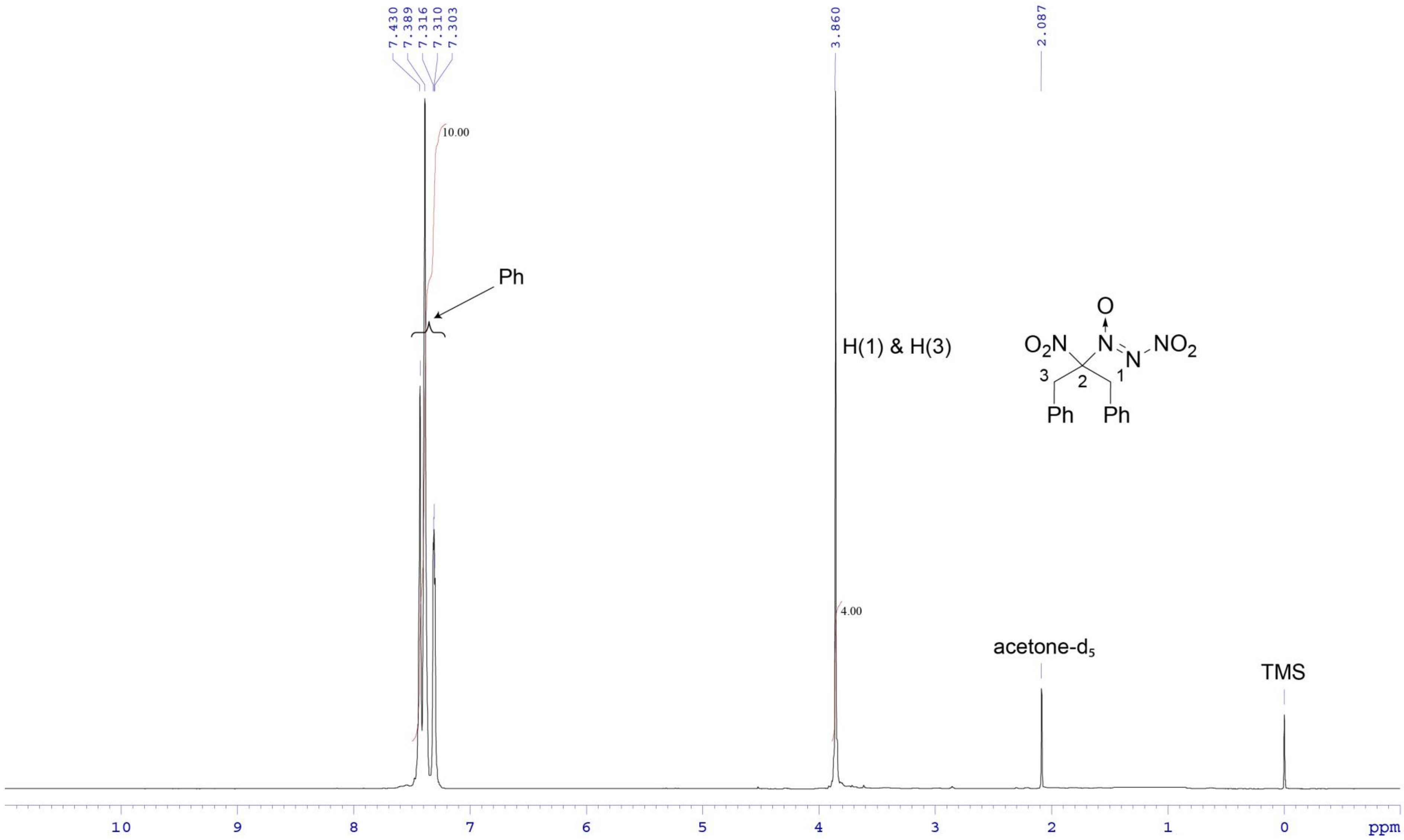

**10.4.2 $^{13}$C NMR spectrum of compound 2e [125.76 MHz, [D$_6$]acetone]**

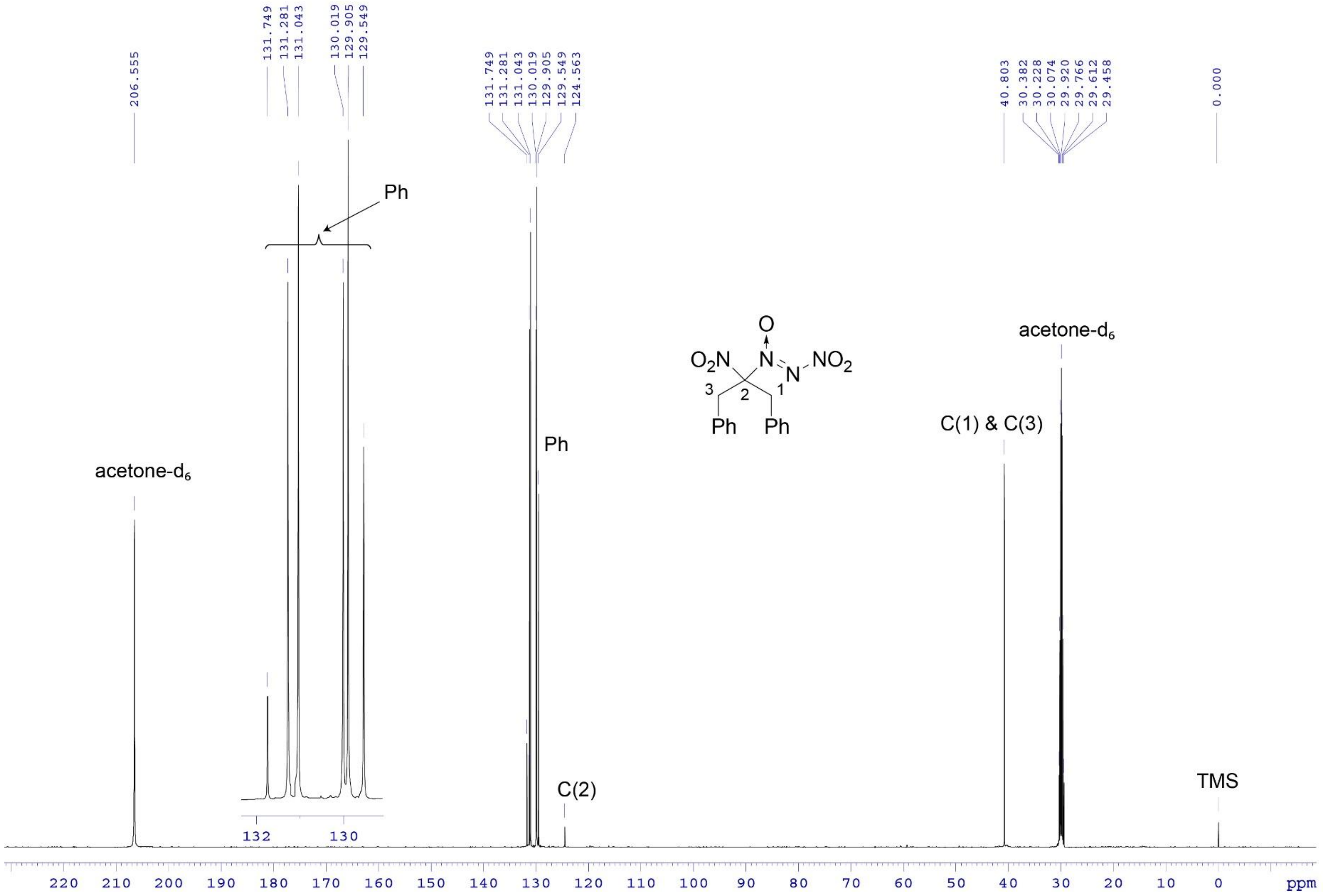

**10.4.3 {$^{1}$H–$^{13}$C} HSQC spectrum of compound 2e [500.13 MHz, [D$_6$]acetone]**

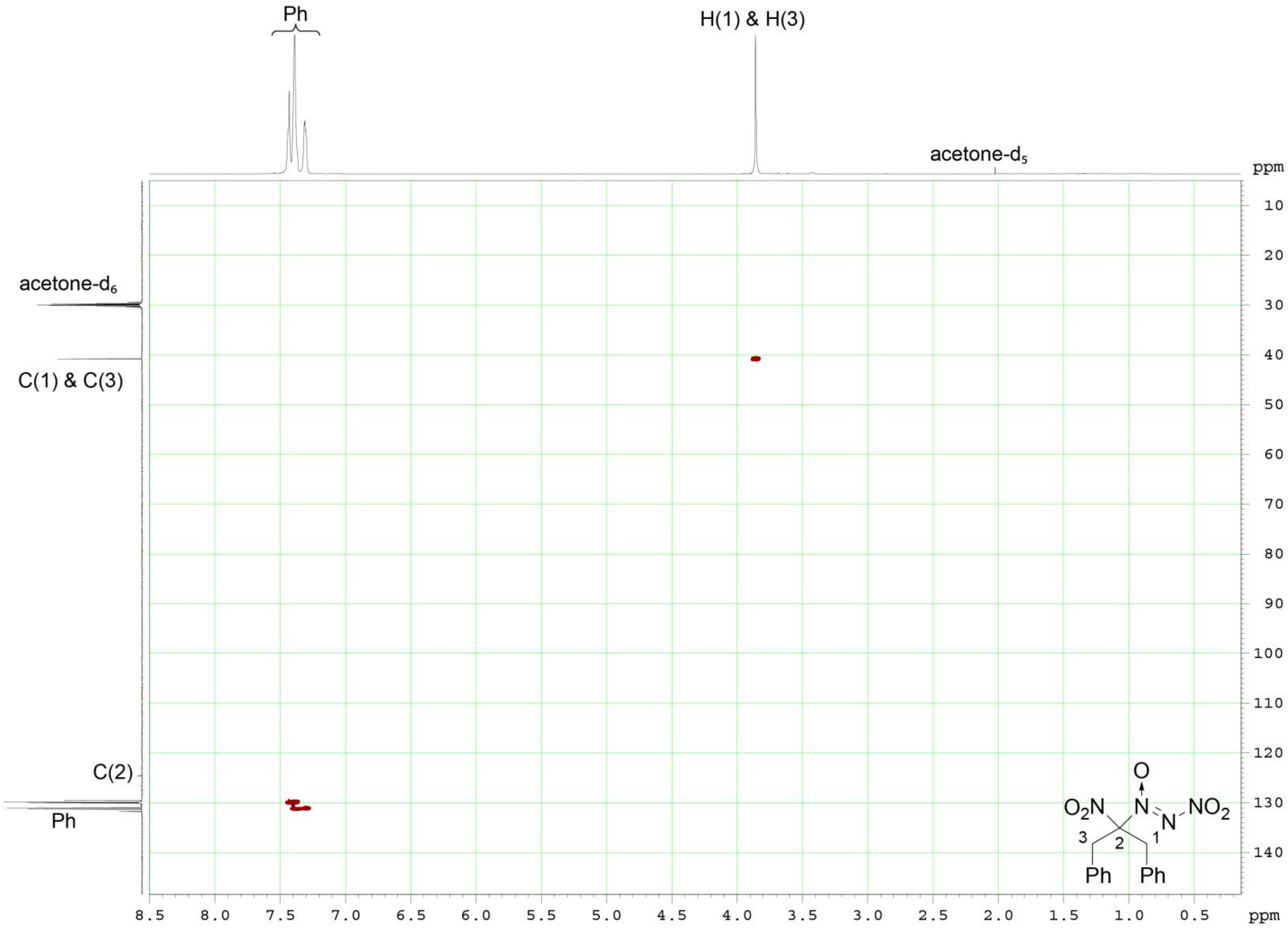

## 10.4.4 {$^1H$–$^{13}C$} HMBC spectrum of compound 2e [500.13 MHz, [$D_6$]acetone]

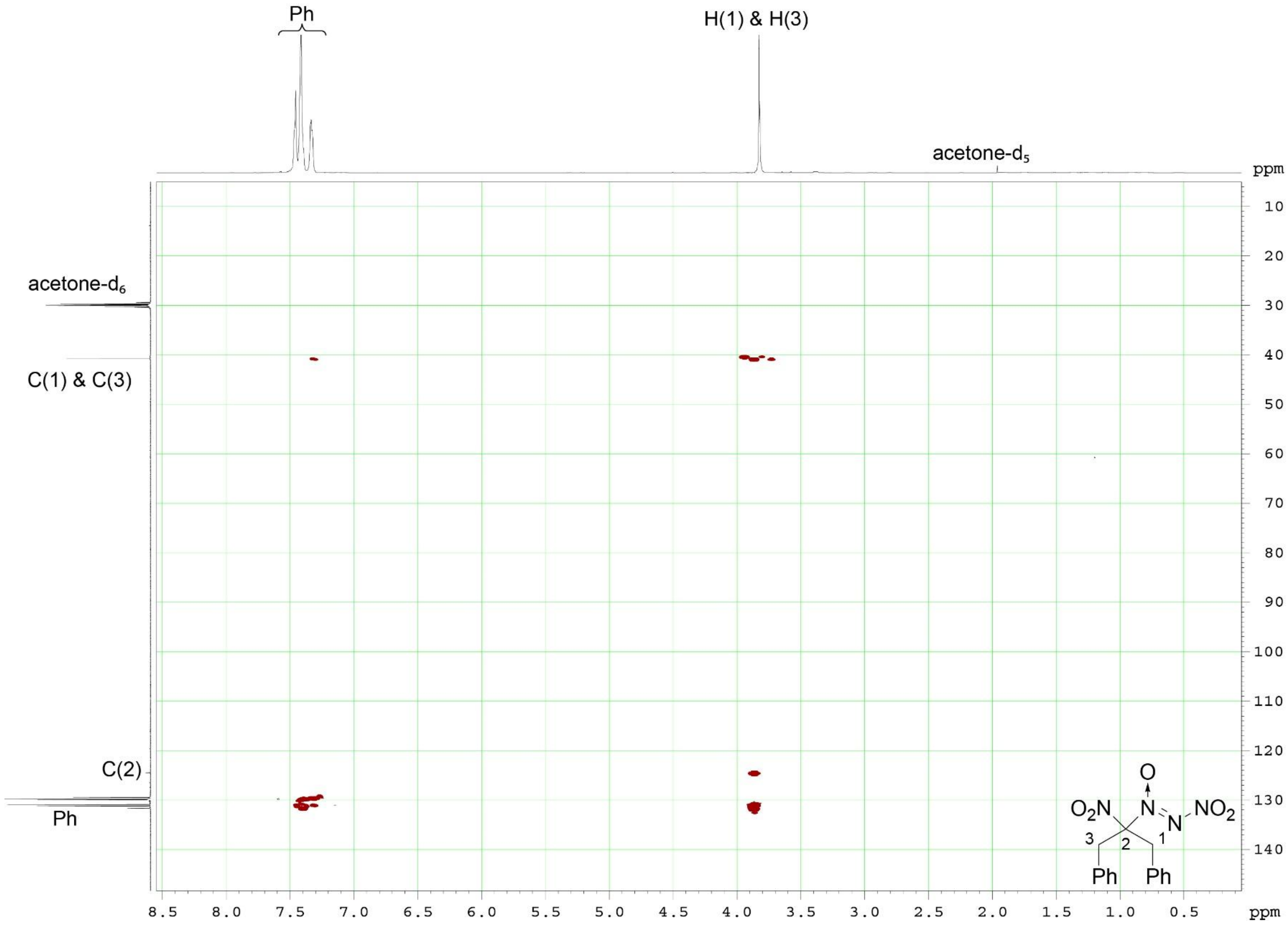

10.4.5 $^{14}N$ NMR spectrum of compound 2e [36.14 MHz, [D$_6$]acetone]

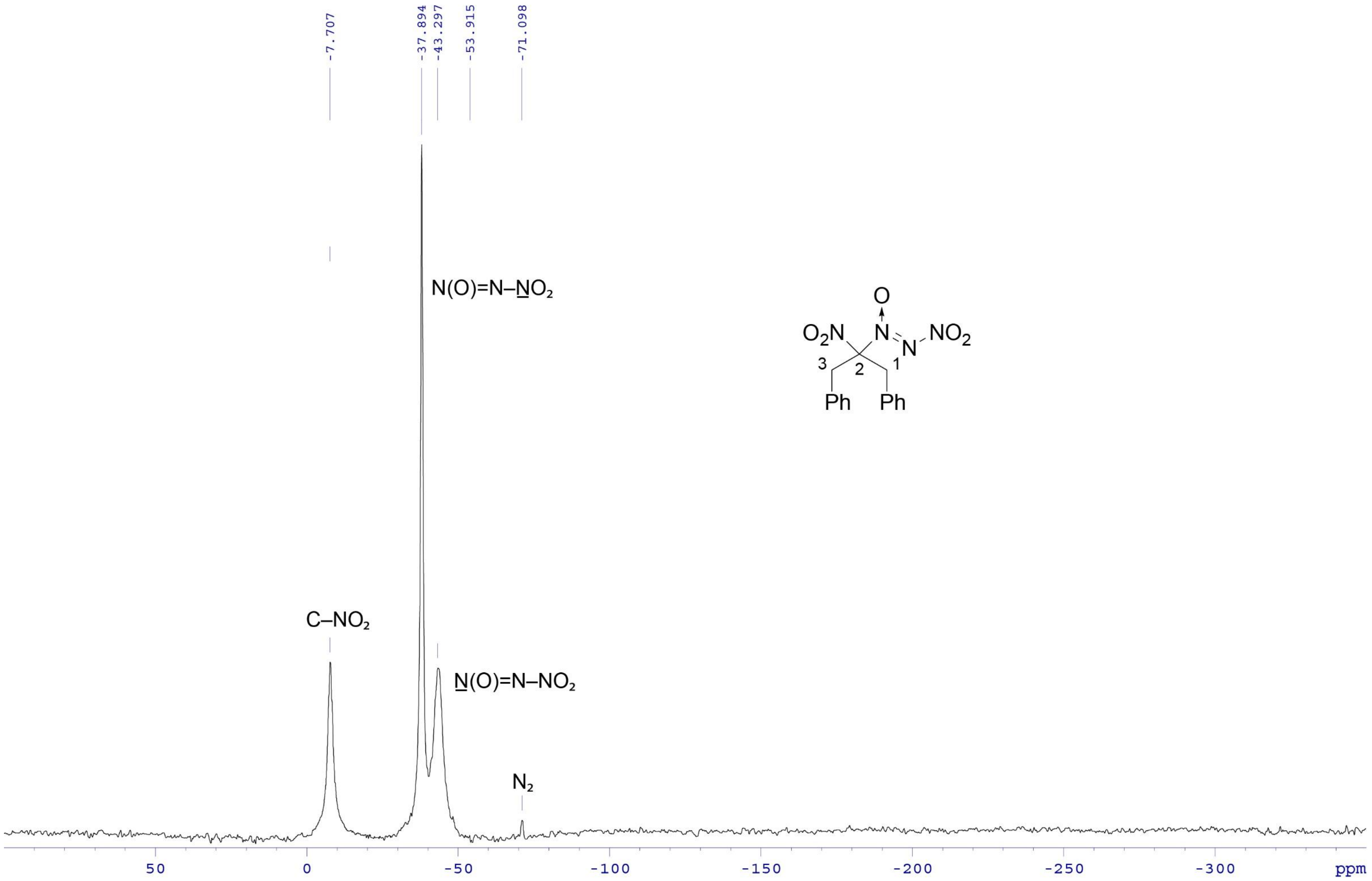

### 10.5.1 $^{1}$H NMR spectrum of compound 2f [500.13 MHz, [D$_6$]acetone]

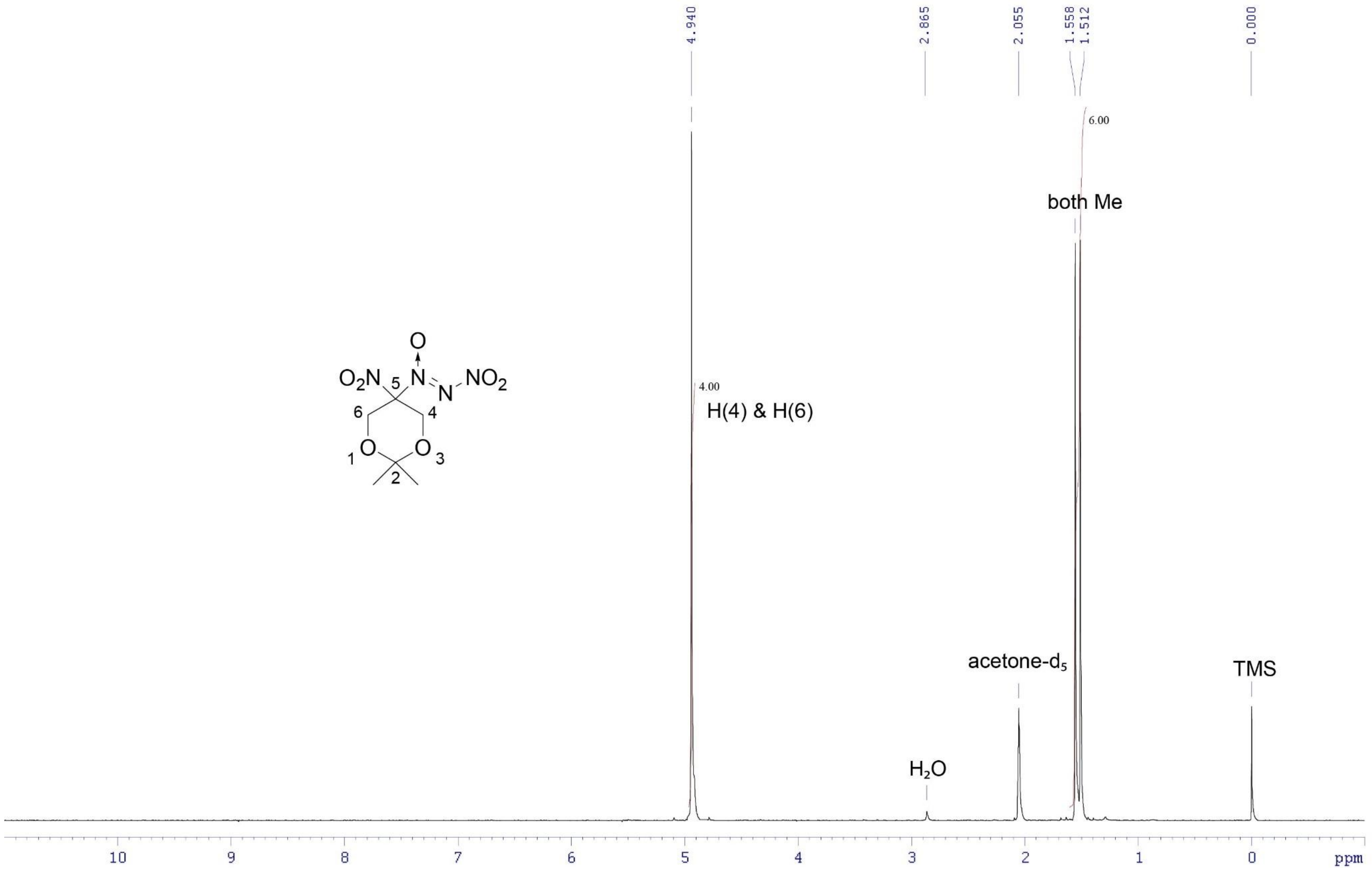

10.5.2 $^{13}C$ NMR spectrum of compound 2f [125.76 MHz, [$D_6$]acetone]

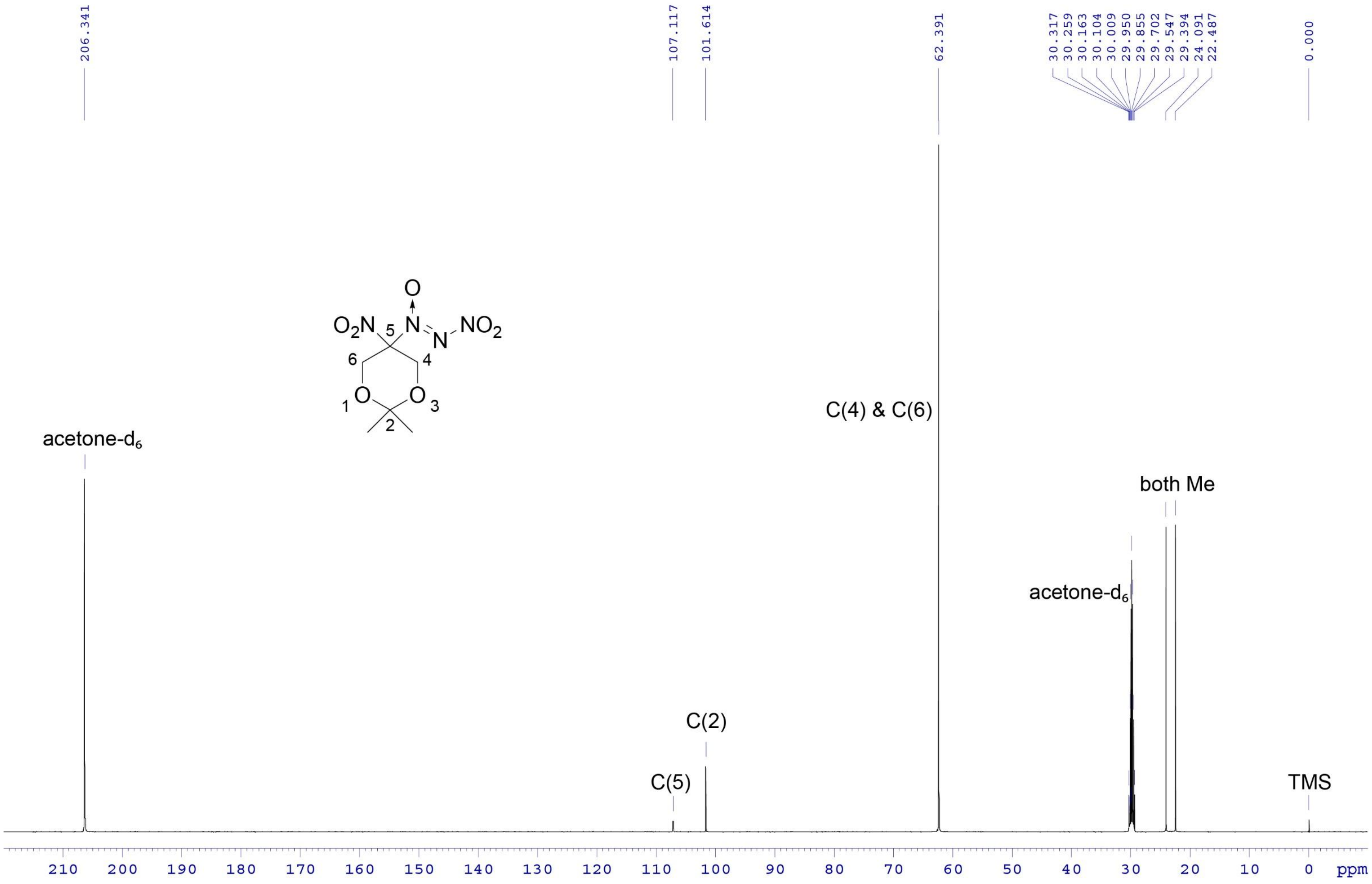

### 10.5.3 {$^1H$–$^{13}C$} HSQC spectrum of compound 2f [500.13 MHz, [D6]acetone]

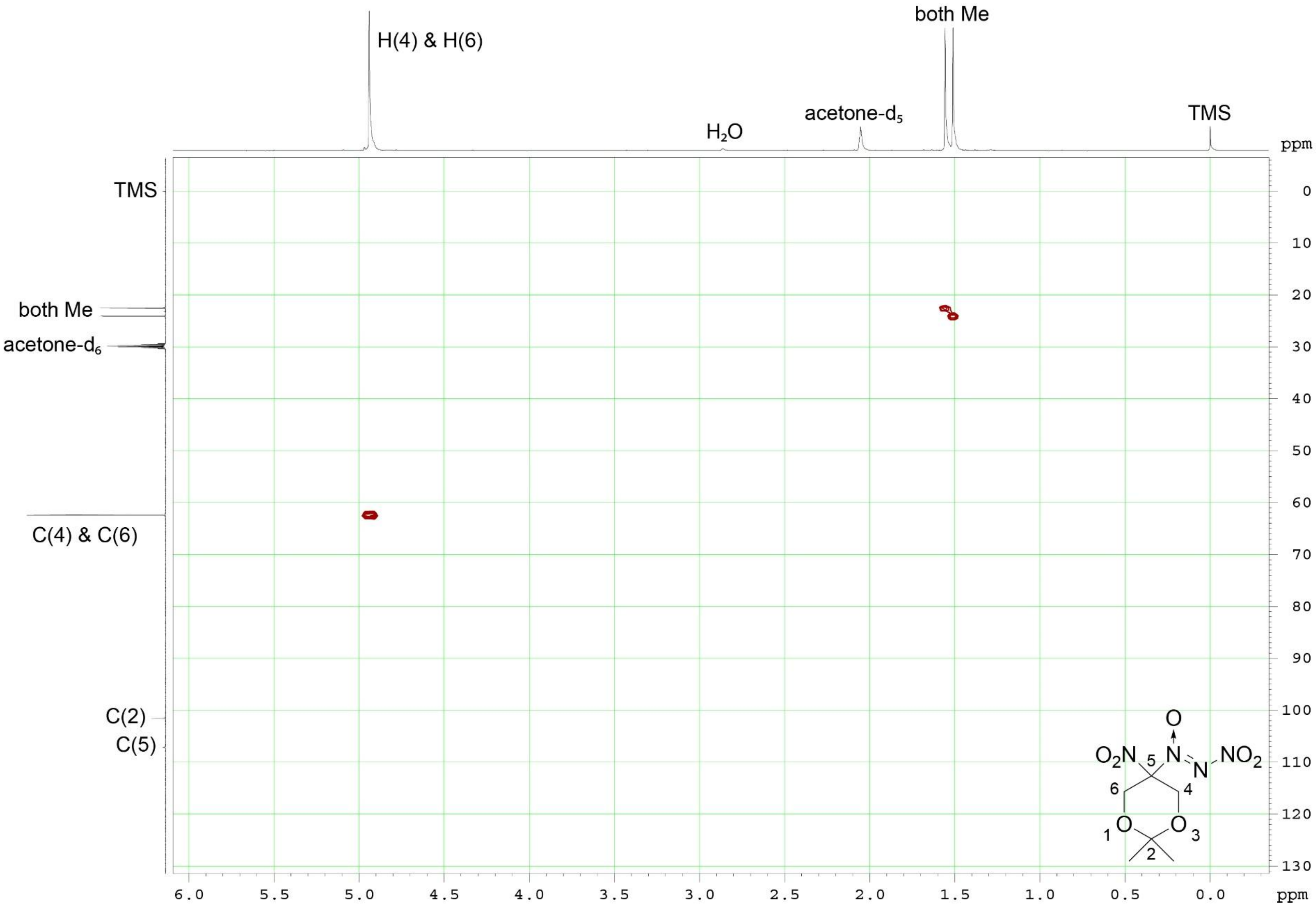

**10.5.4 {$^{1}$H–$^{13}$C} HMBC spectrum of compound 2f [500.13 MHz, [D6]acetone]**

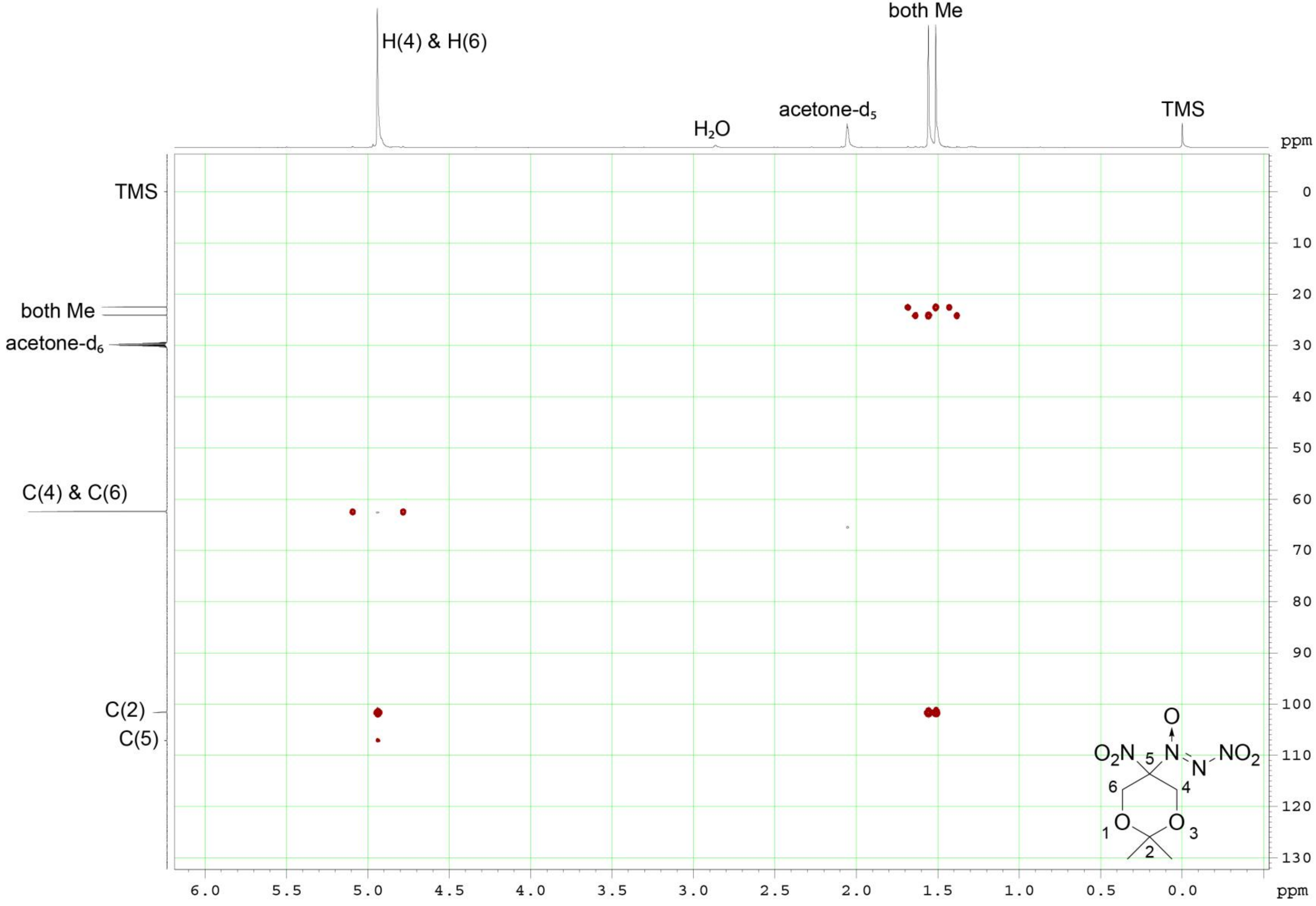

**10.5.5 $^{14}$N NMR spectrum of compound 2f [36.14 MHz, [D$_6$]acetone]**

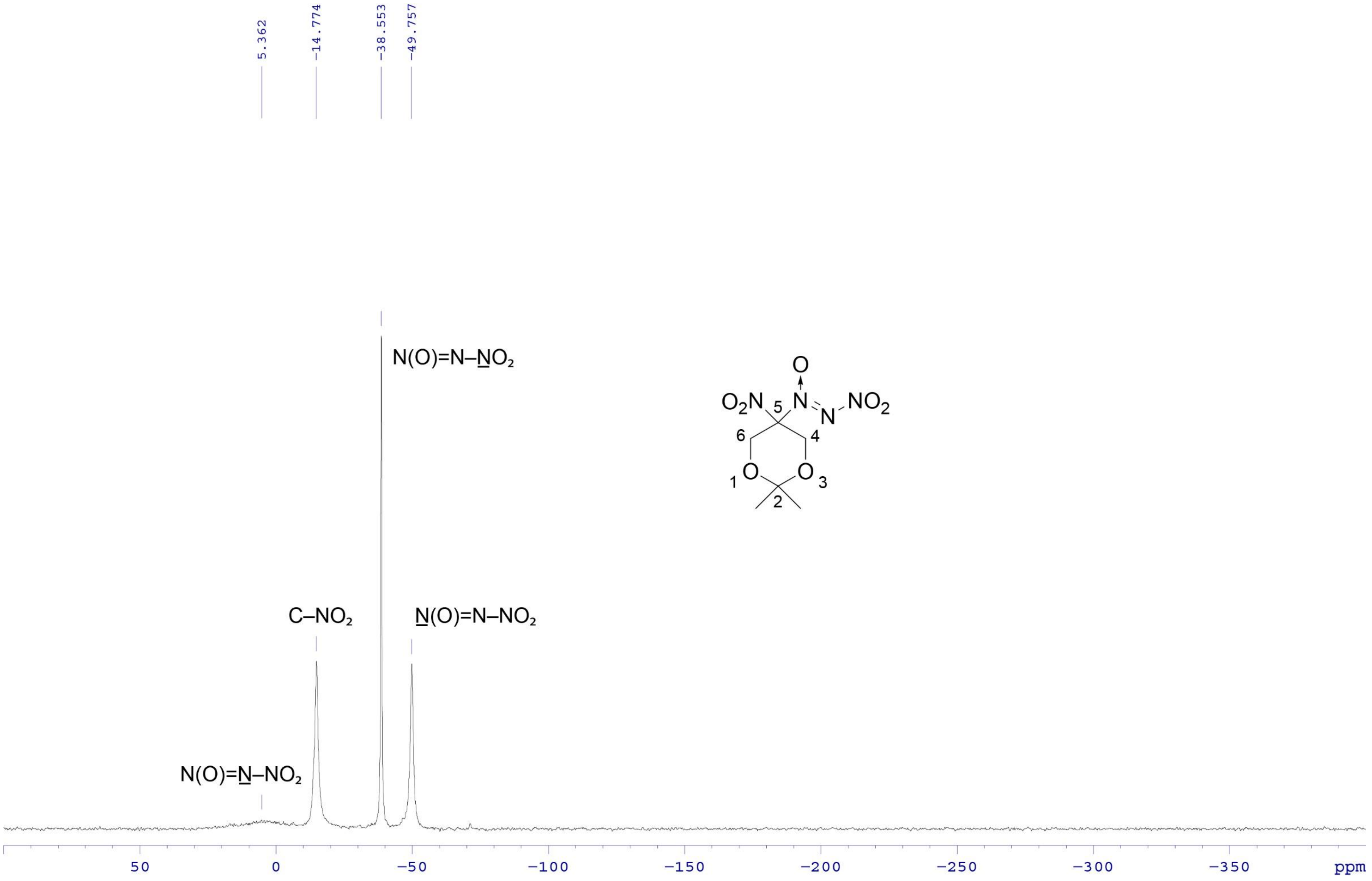

**10.6.1 $^{1}$H NMR spectrum of compound 2g [600.13 MHz, $CDCl_3$]**

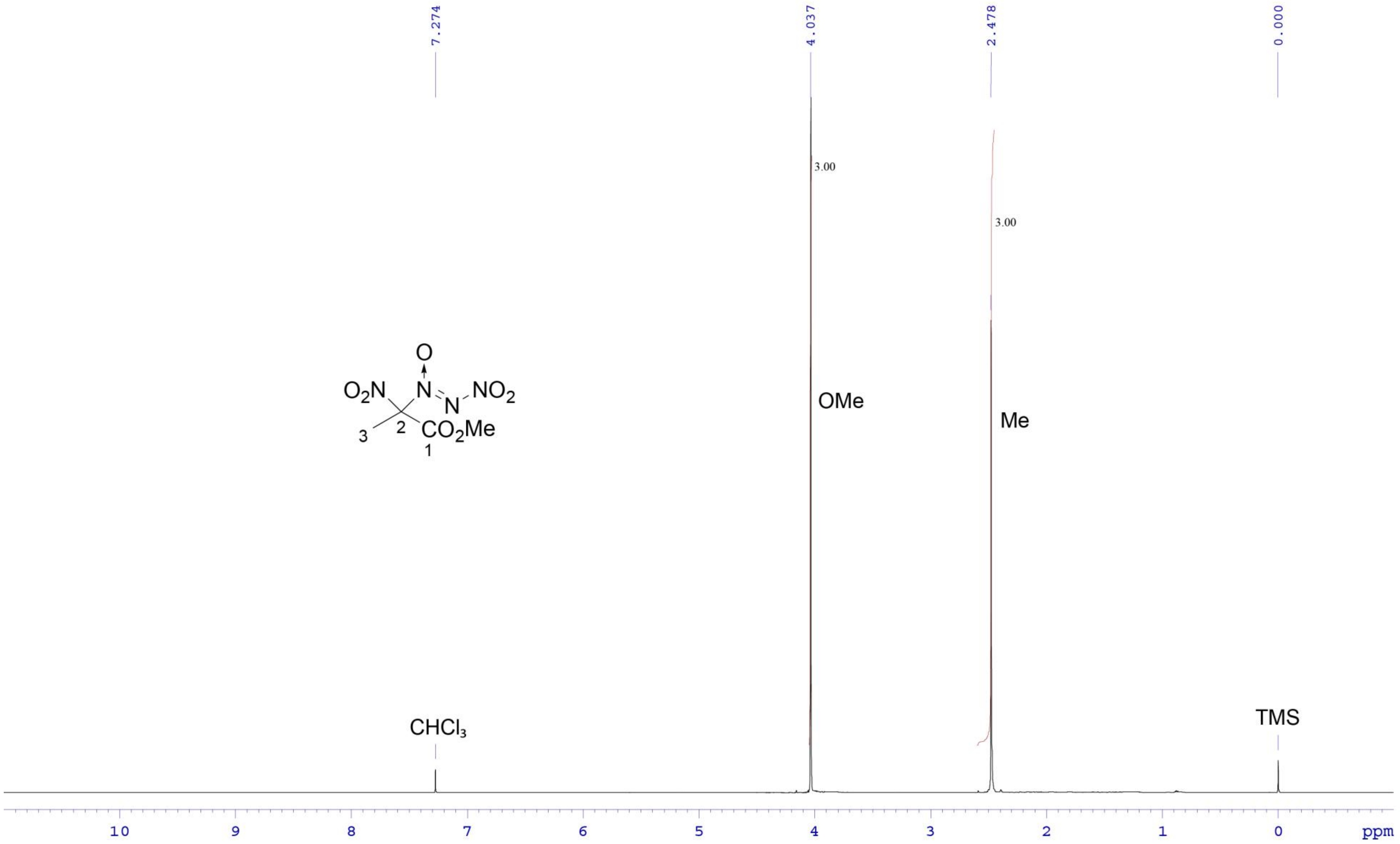

**10.6.2 $^{13}$C NMR spectrum of compound 2g [150.90 MHz, $CDCl_3$]**

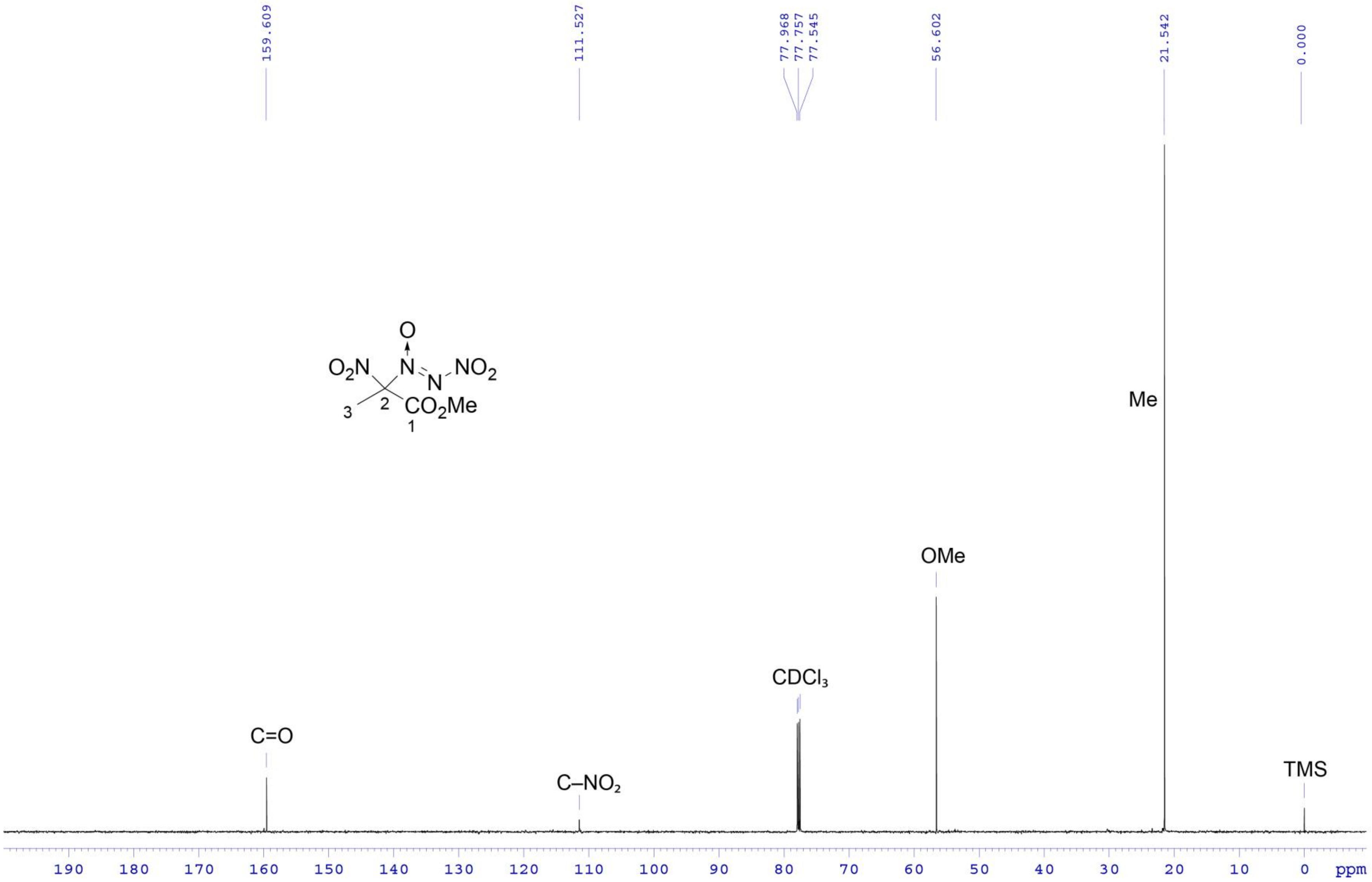

### 10.6.3 {$^{1}$H–$^{13}$C} HSQC spectrum of compound 2g [600.13 MHz, $CDCl_3$]

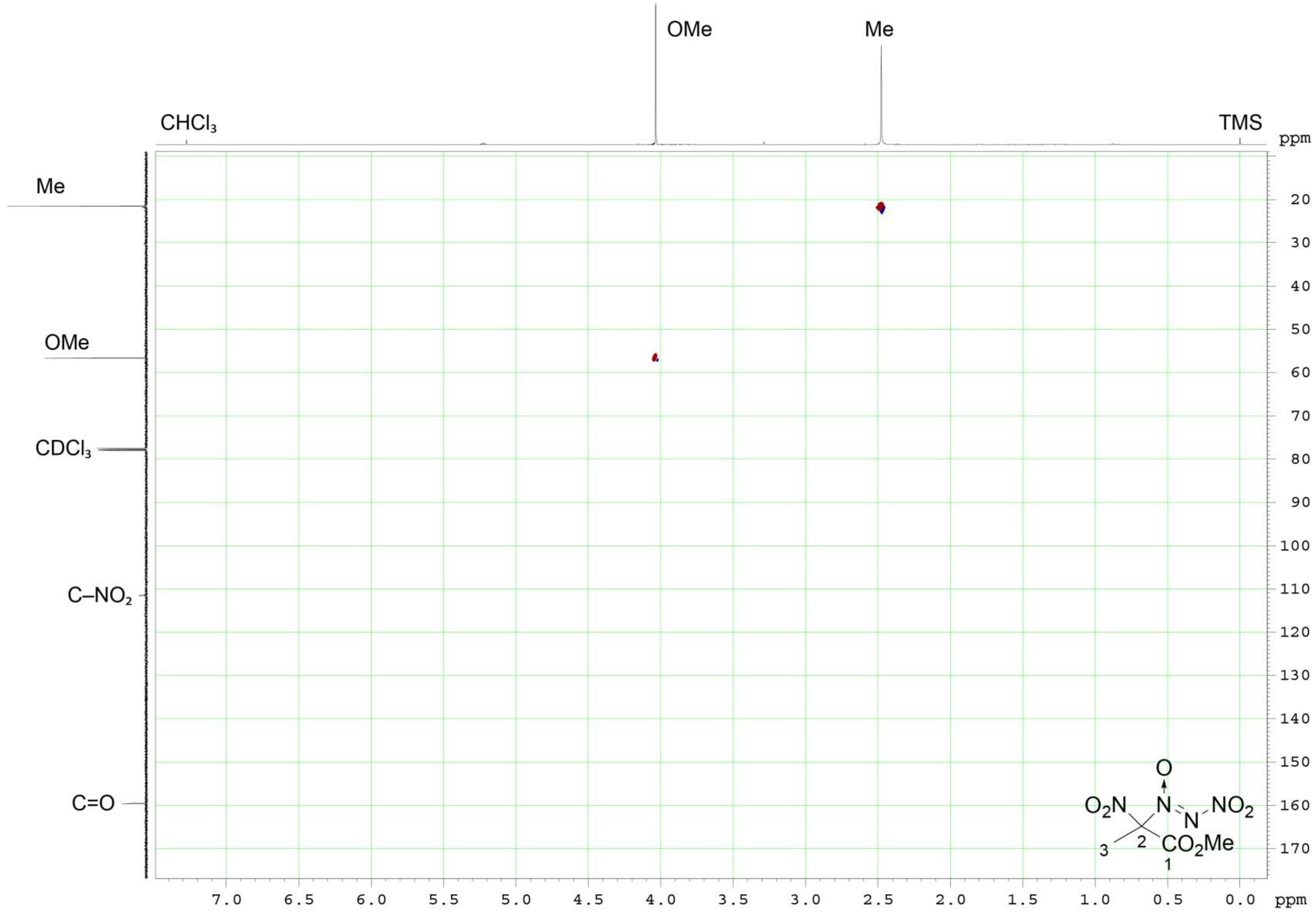

### 10.6.4 {$^1$H–$^{13}$C} HMBC spectrum of compound 2g [600.13 MHz, $CDCl_3$]

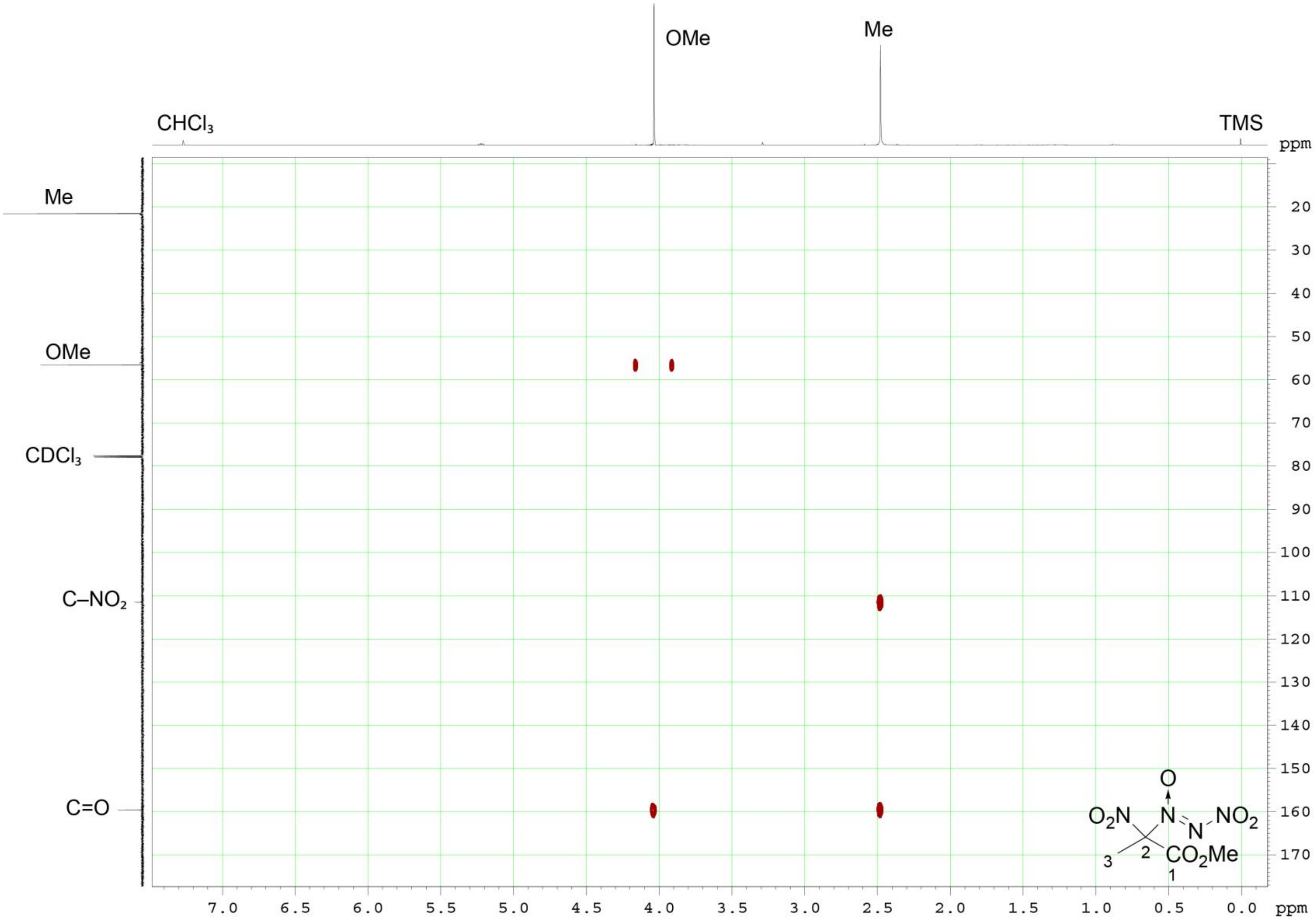

**10.6.5 $^{14}$N NMR spectrum of compound 2g [43.37 MHz, $CDCl_3$]**

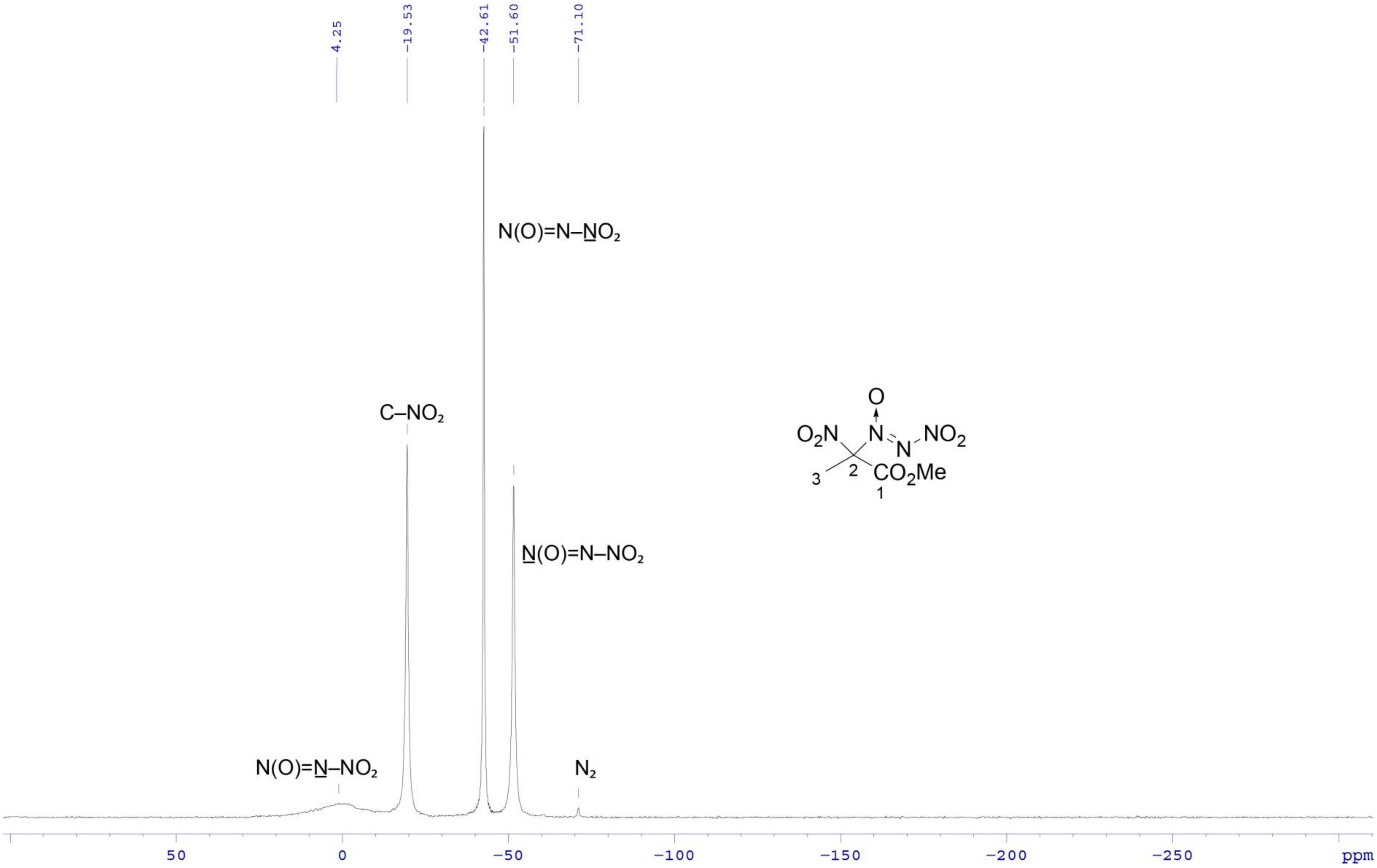

### 10.7.1 $^{1}$H NMR spectrum of compound 2h [600.13 MHz, $CDCl_3$]

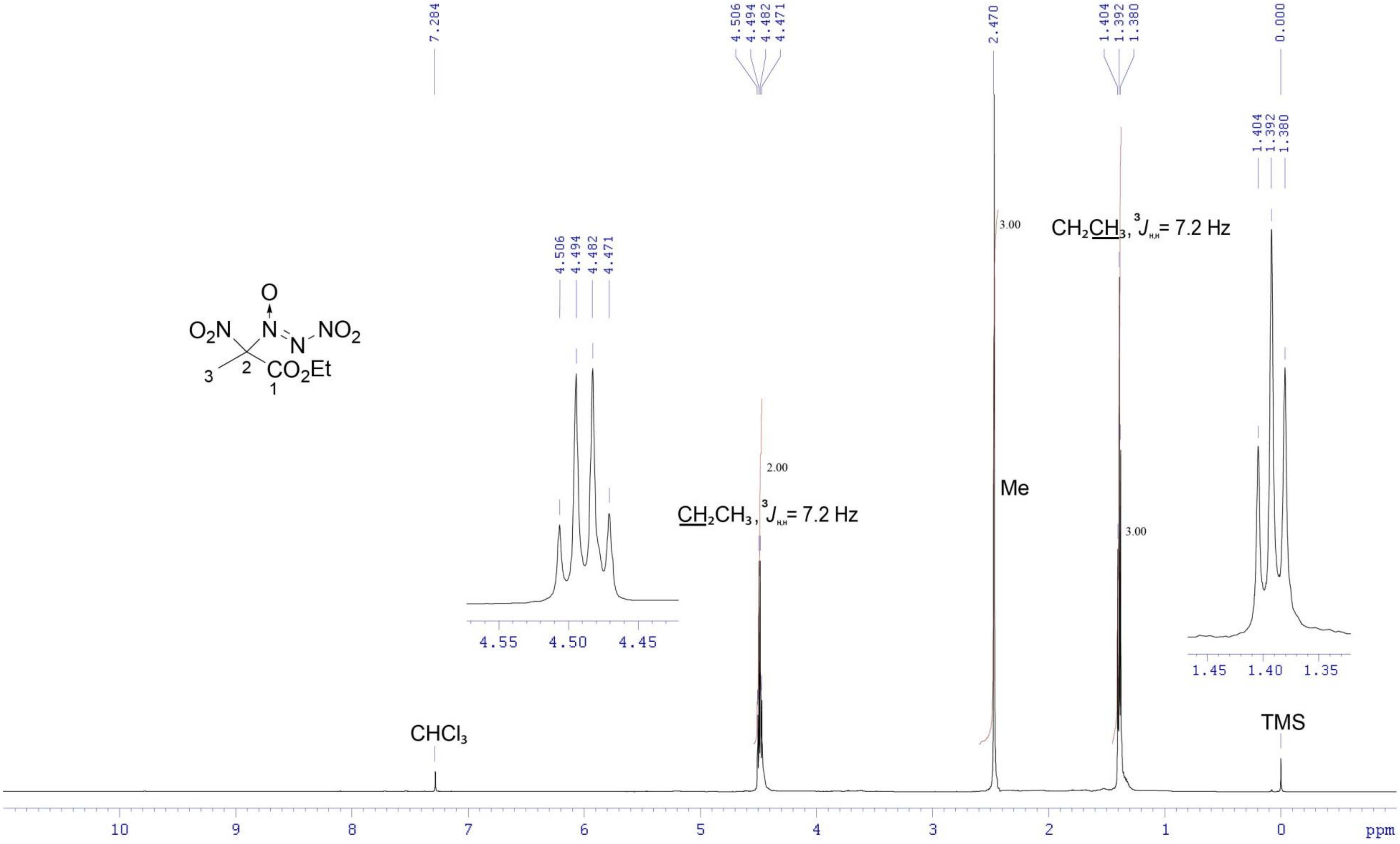

**10.7.2 $^{13}C$ NMR spectrum of compound 2h [150.90 MHz, $CDCl_3$]**

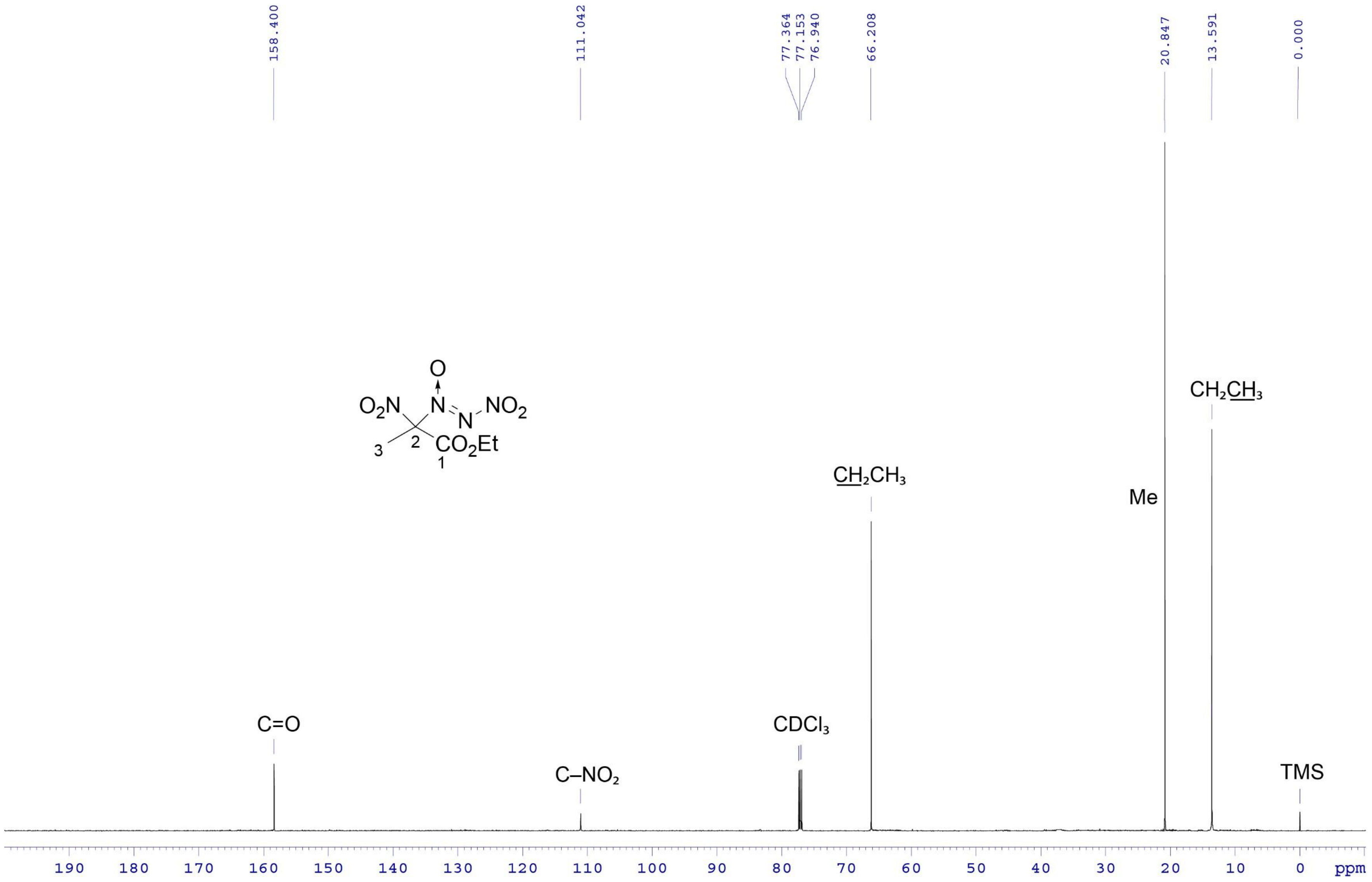

10.7.3 {$^{1}$H–$^{13}$C} HSQC spectrum of compound 2h [600.13 MHz, $CDCl_3$]

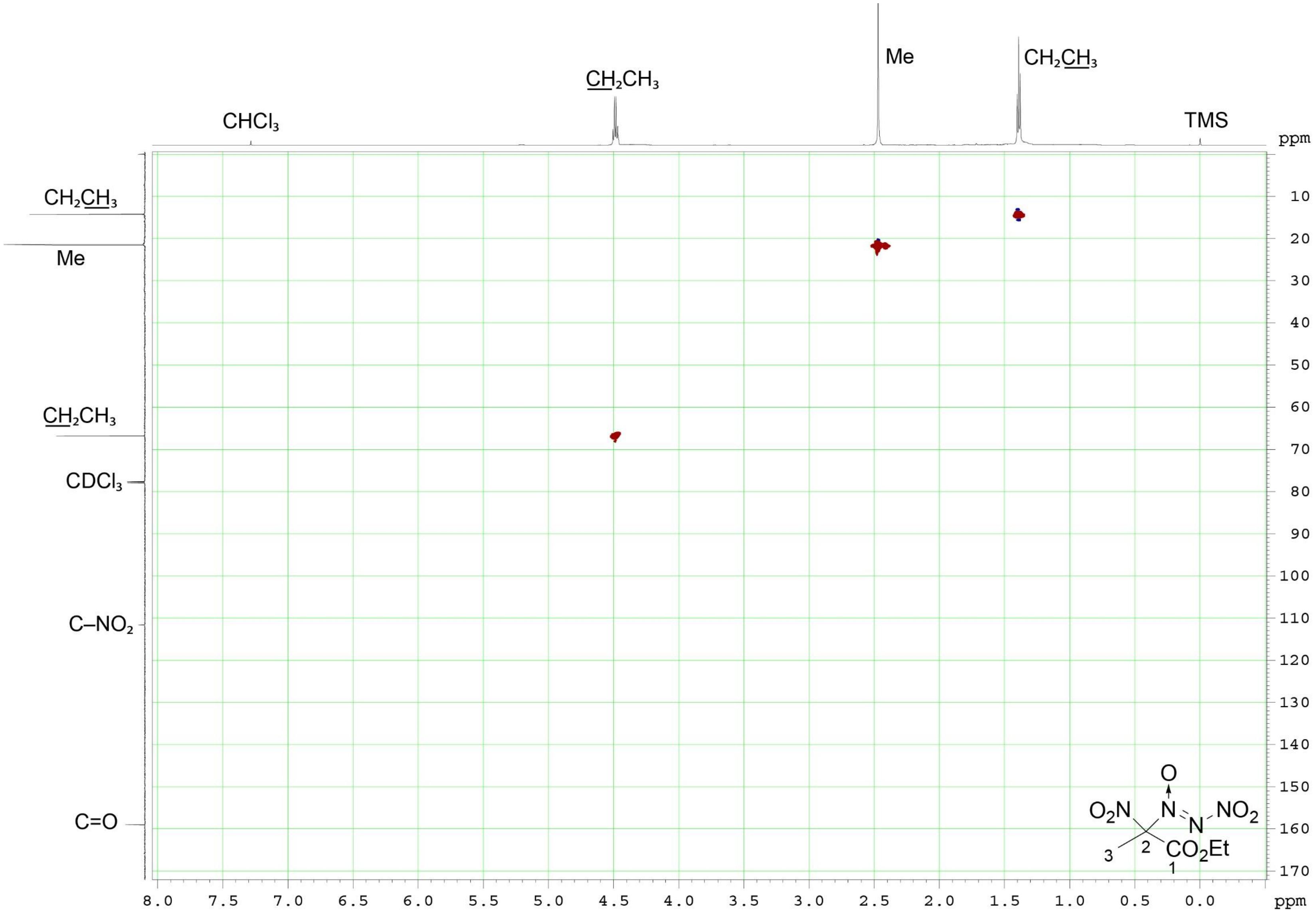

### 10.7.4 {$^1H$–$^{13}C$} HMBC spectrum of compound 2h [600.13 MHz, $CDCl_3$]

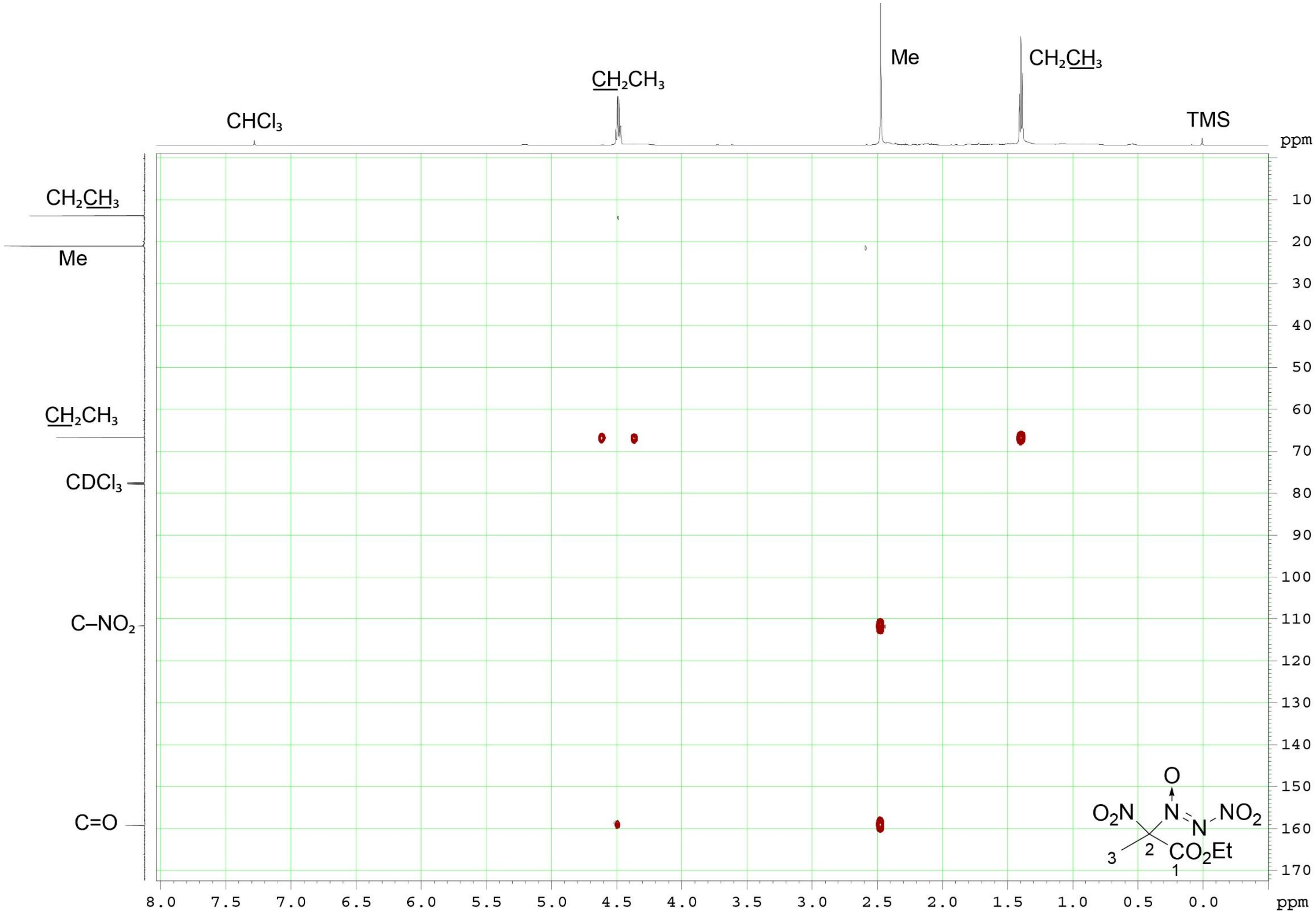

### 10.7.5 $^{14}$N NMR spectrum of compound 2h [43.14 MHz, $CDCl_3$]

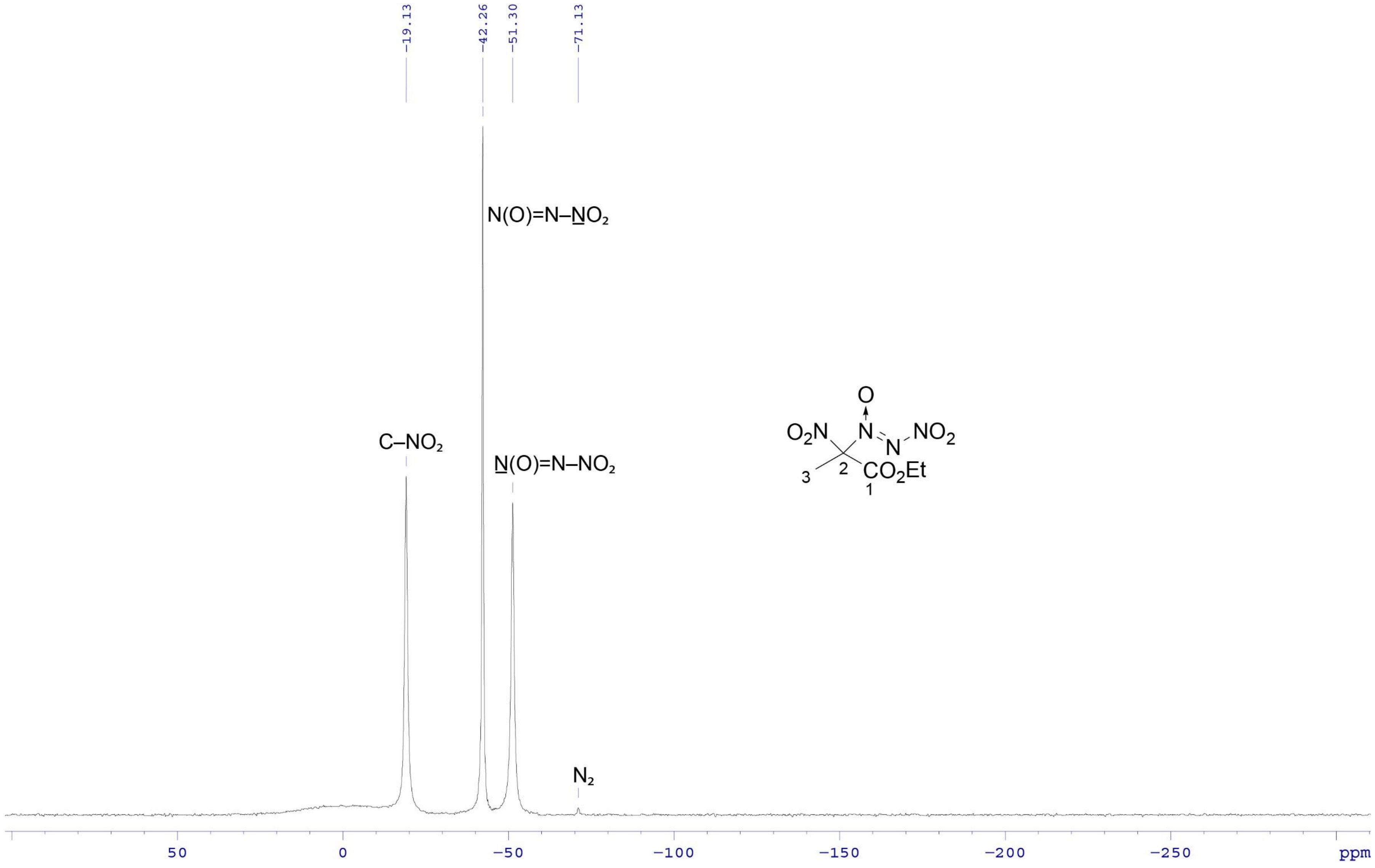

### 10.8.1 $^{1}H$ NMR spectrum of compound 2i [500.13 MHz, $CDCl_3$]

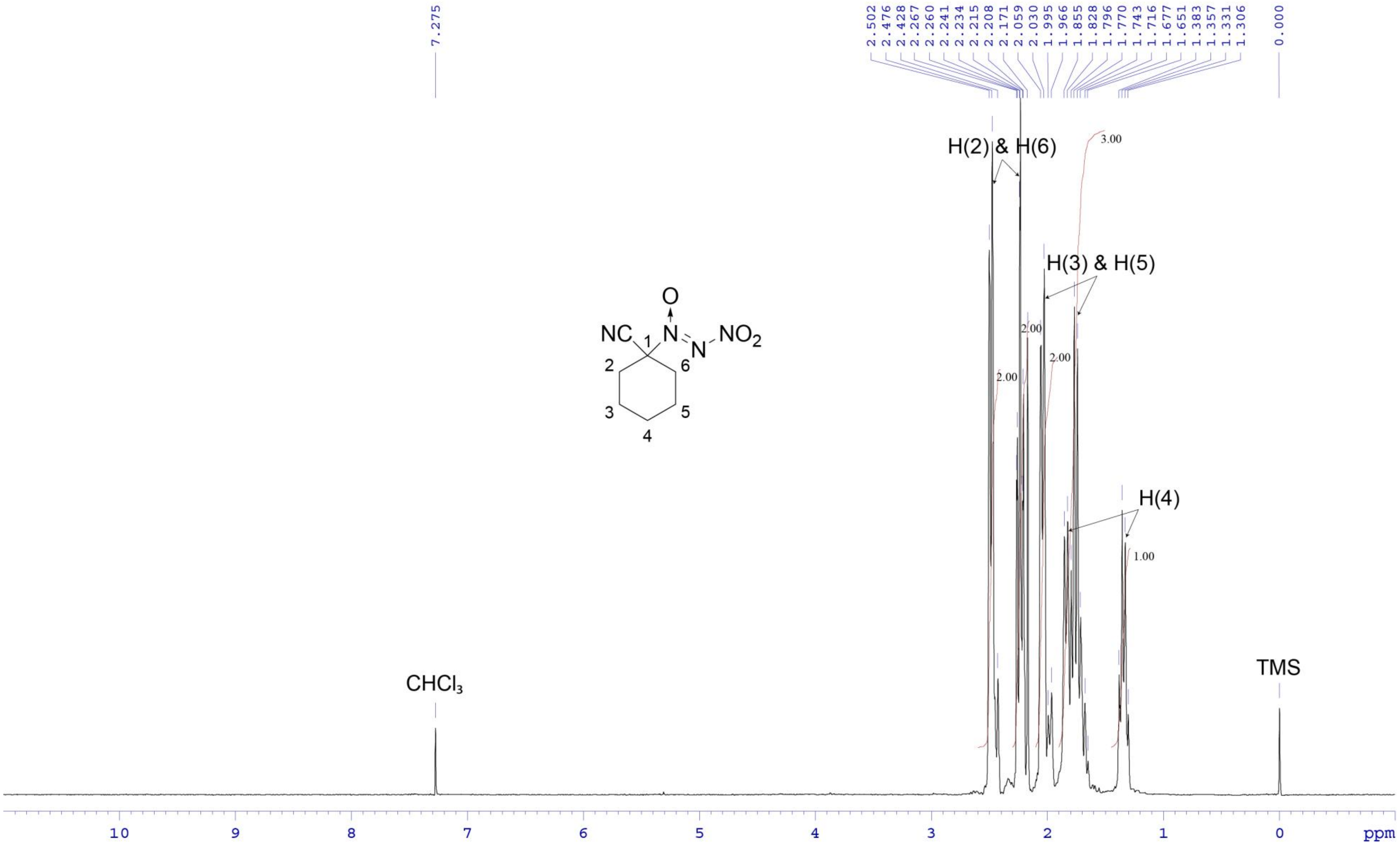

**10.8.2 $^{13}C$ NMR spectrum of compound 2i [125.76 MHz, $CDCl_3$]**

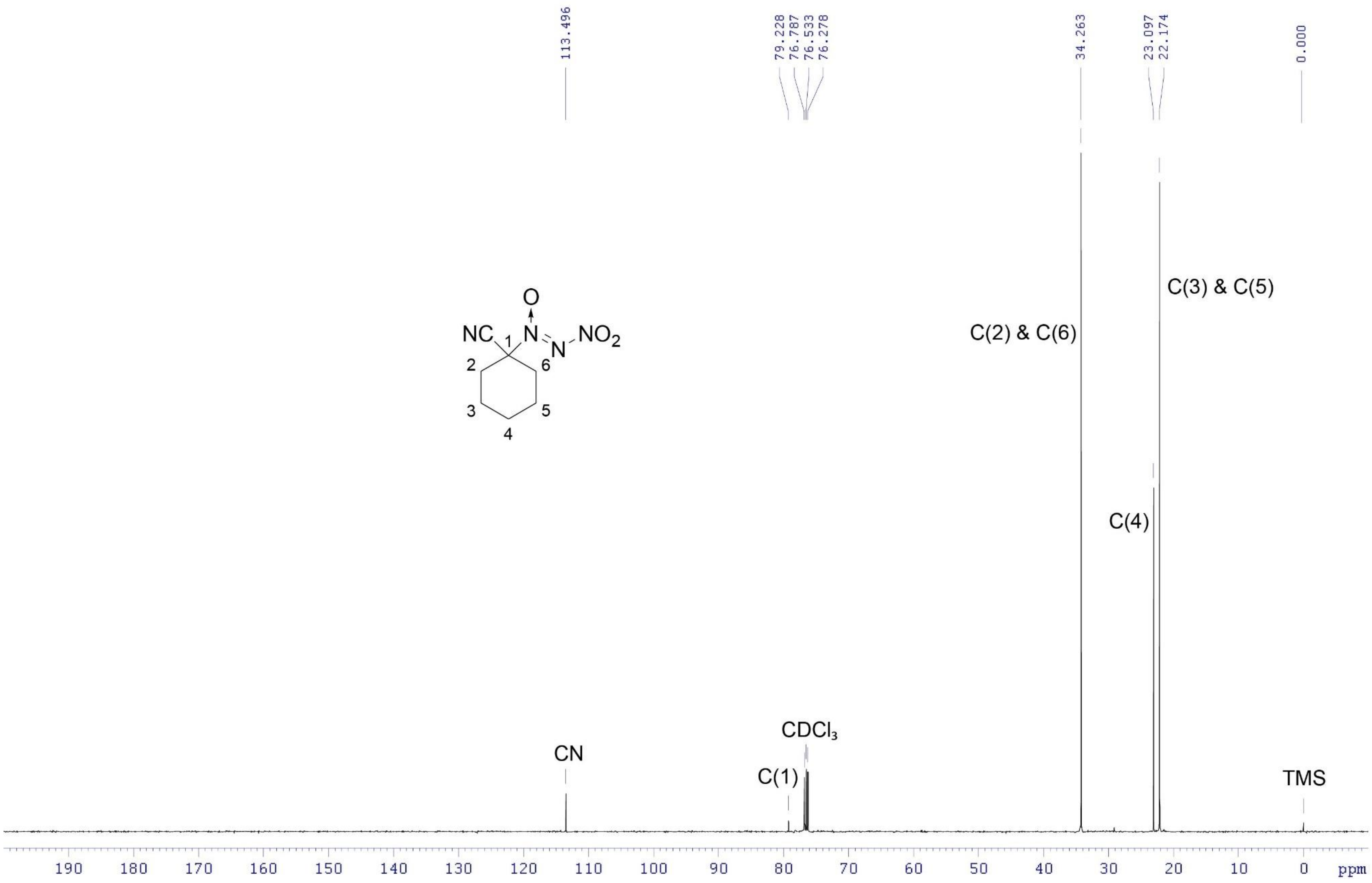

**10.8.3 {$^1H$–$^{13}C$} HSQC spectrum of compound 2i [500.13 MHz, $CDCl_3$]**

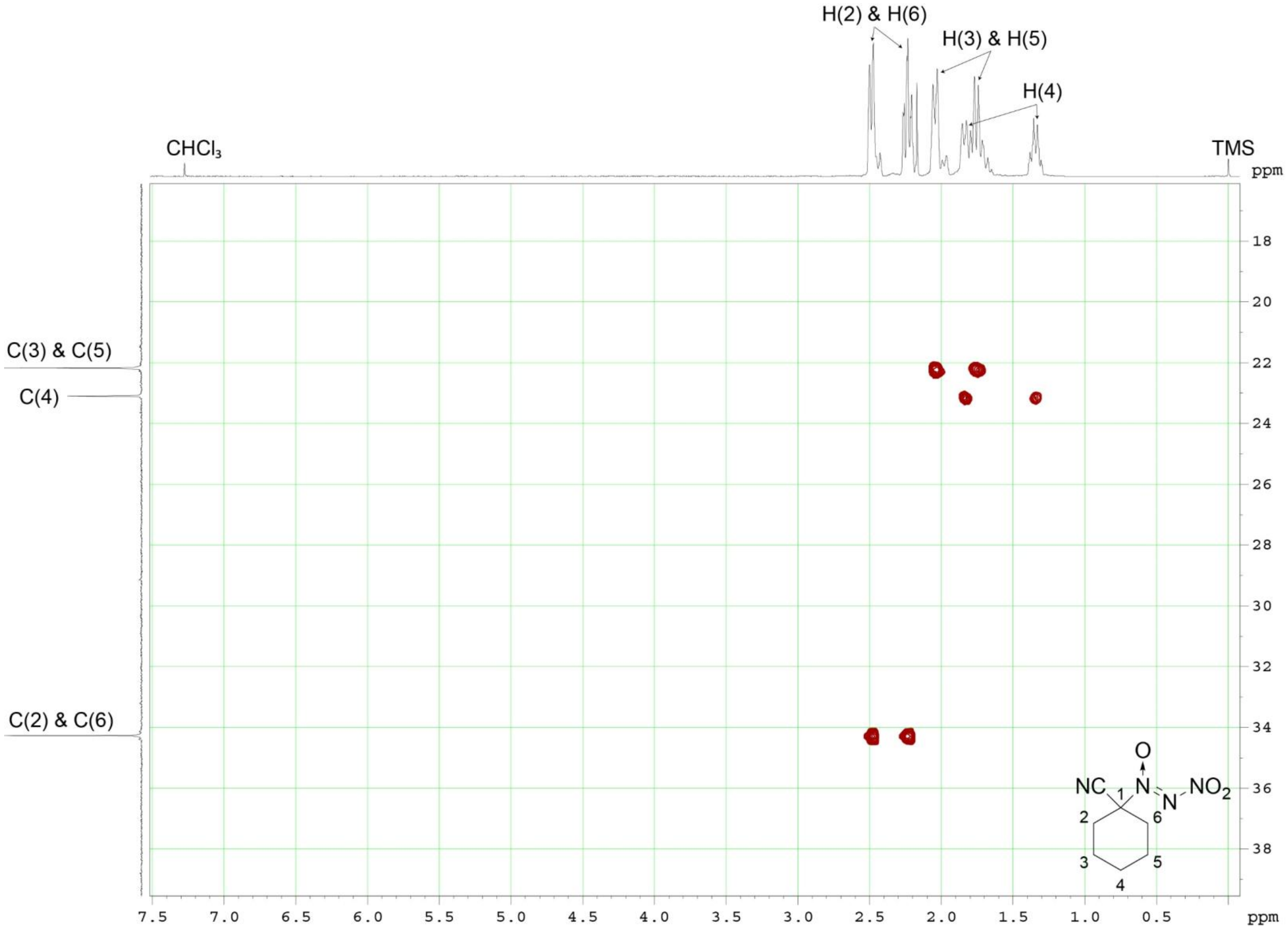

**10.8.4 {$^{1}H–^{13}C$} HMBC spectrum of compound 2i [500.13 MHz, $CDCl_3$]**

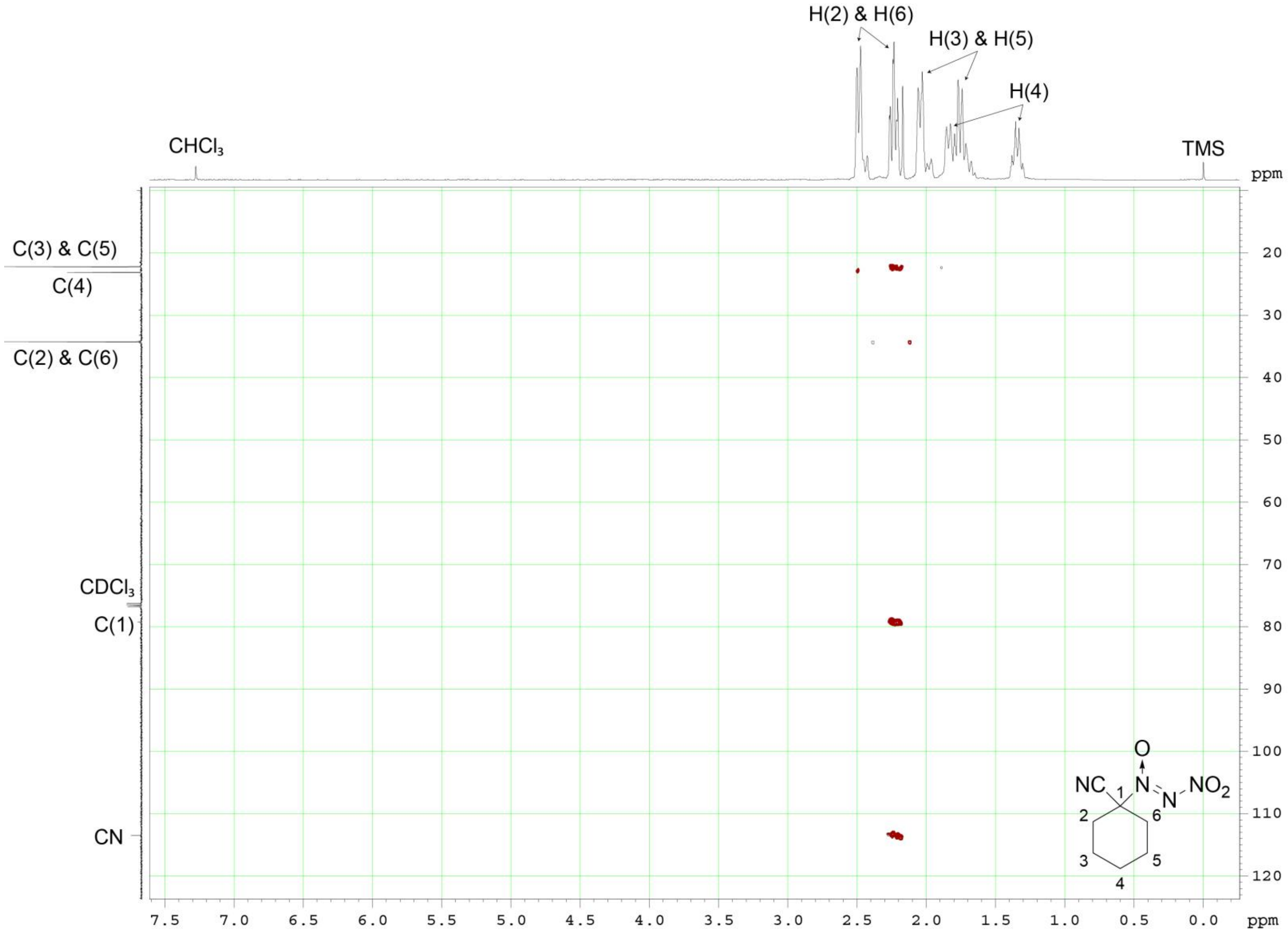

**10.8.5 $^{14}$N NMR spectrum of compound 2i [36.14 MHz, $CDCl_3$]**

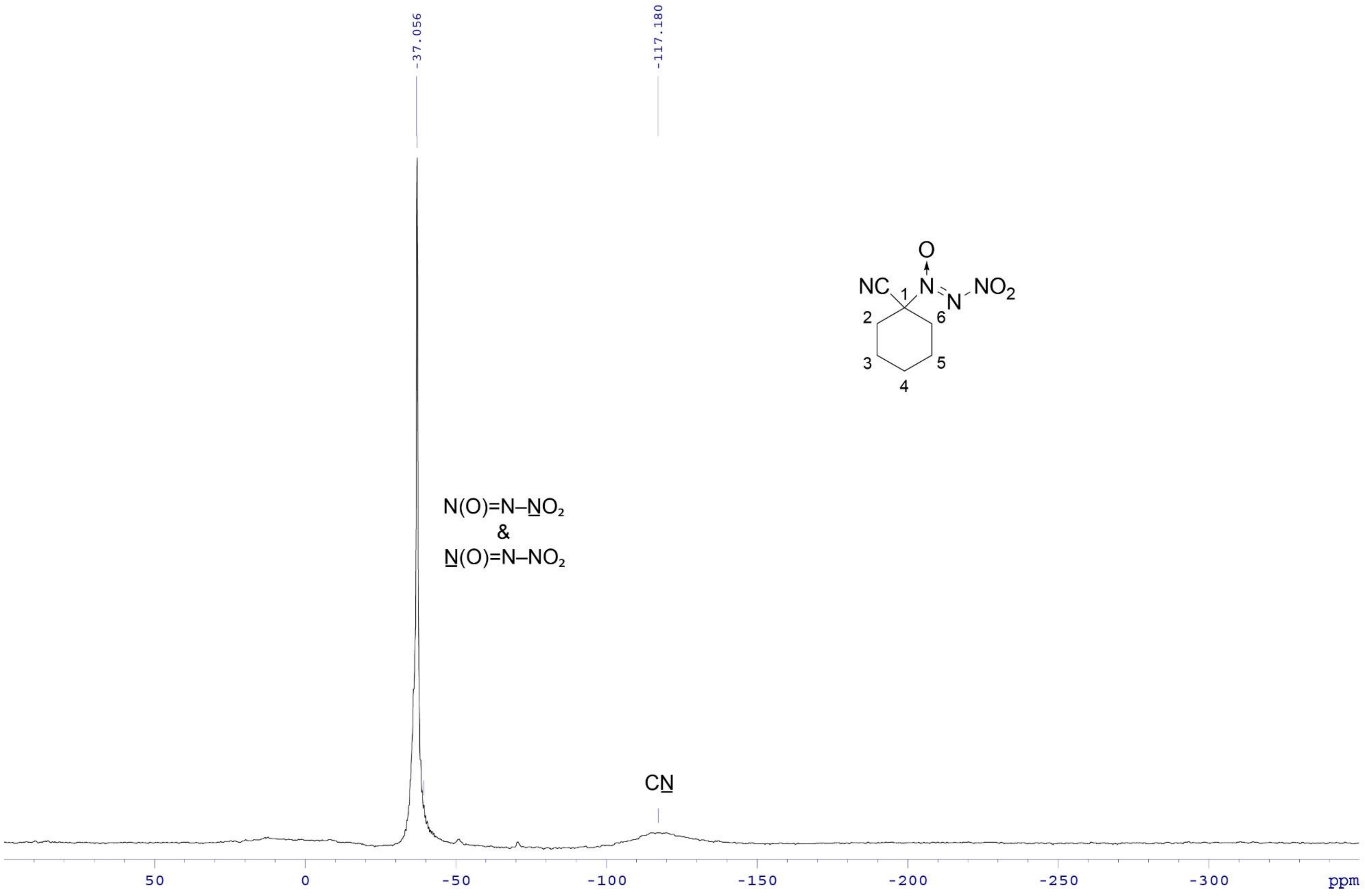

### 10.9.1 $^1$H NMR spectrum of compound 3f [500.13 MHz, [D$_6$]acetone]

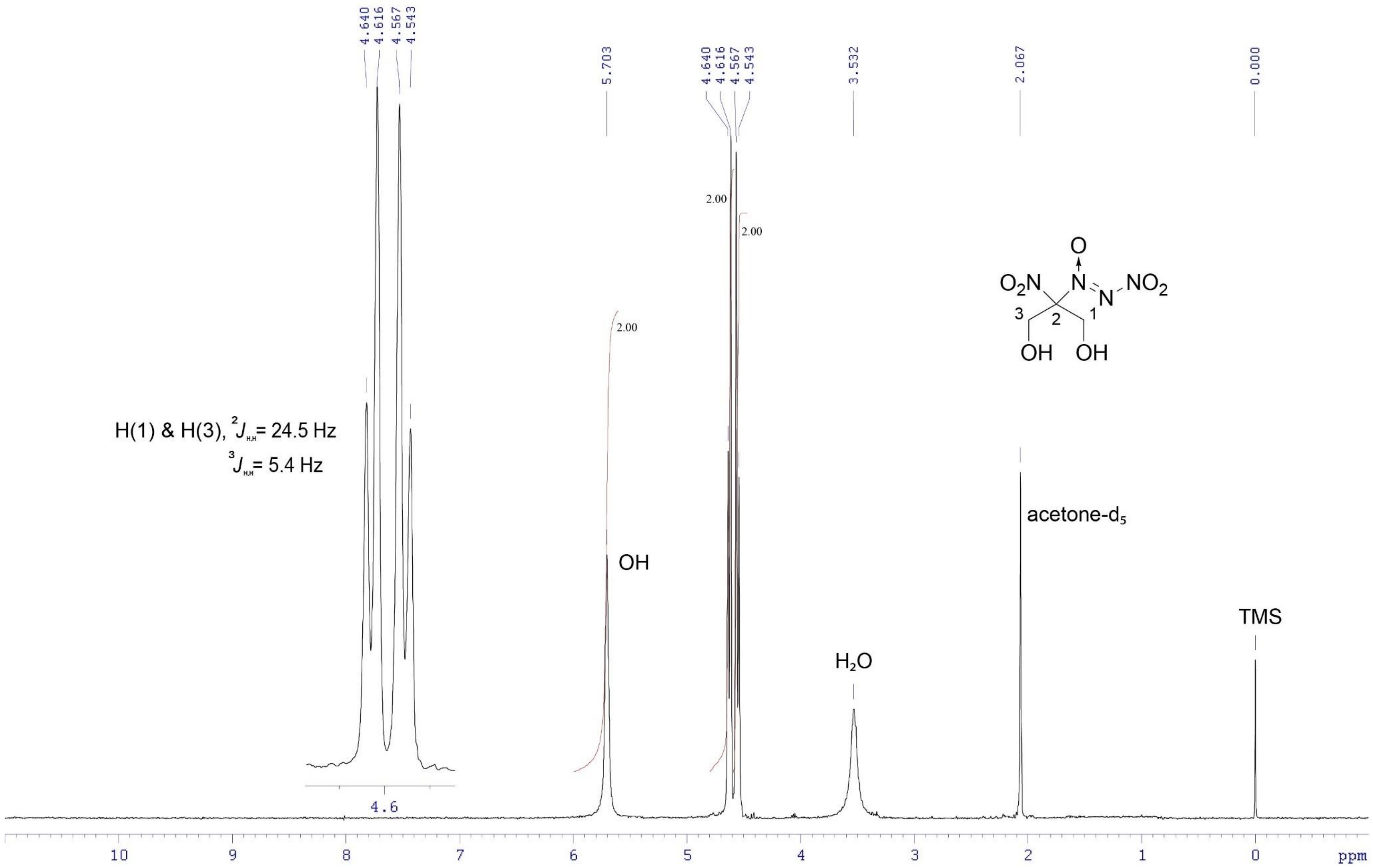

**10.9.2 $^{13}C$ NMR spectrum of compound 3f [125.76 MHz, [D$_6$]acetone]**

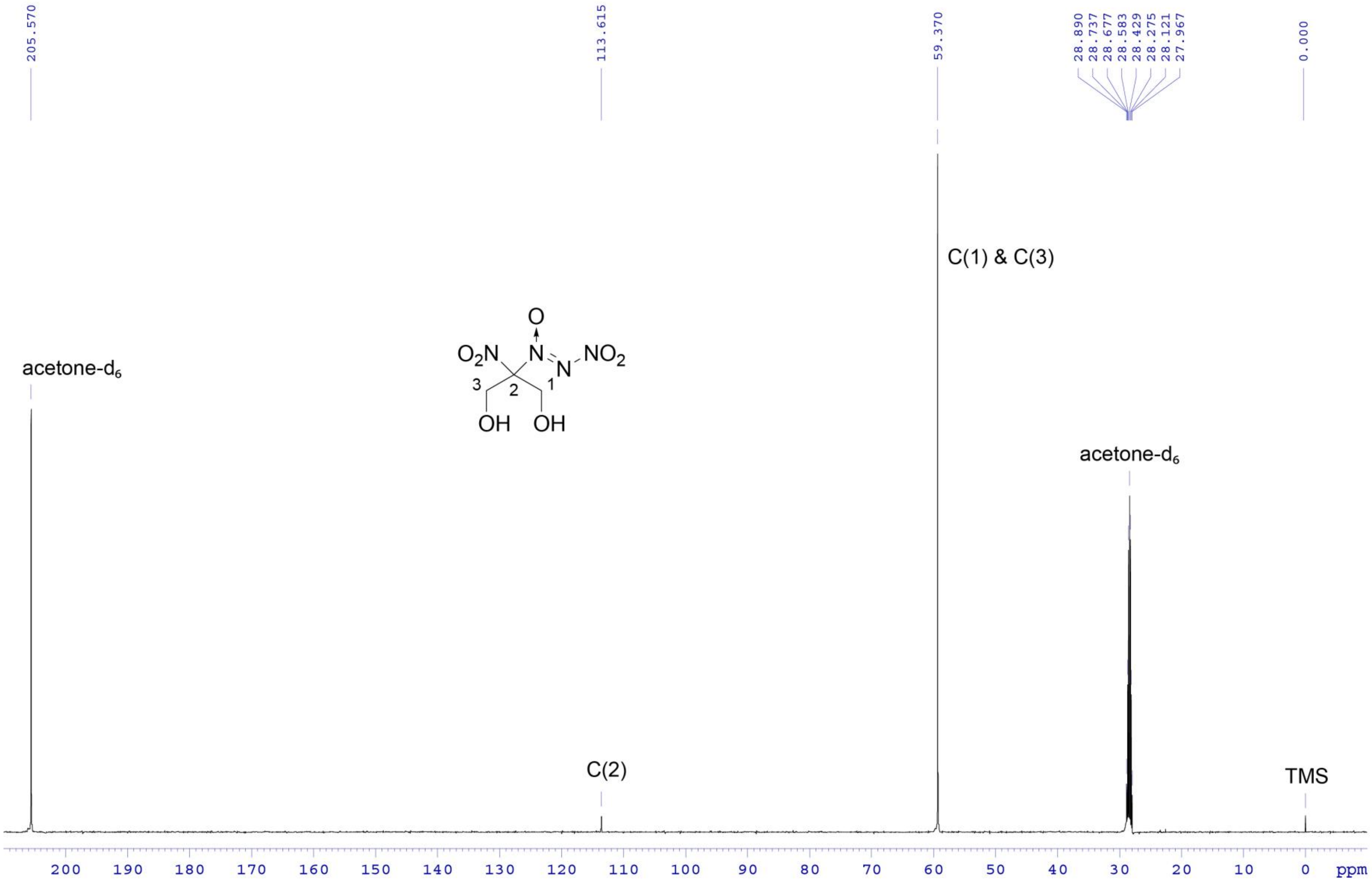

### 10.9.3 {$^{1}$H–$^{13}$C} HSQC spectrum of compound 3f [500.13 MHz, [D6]acetone]

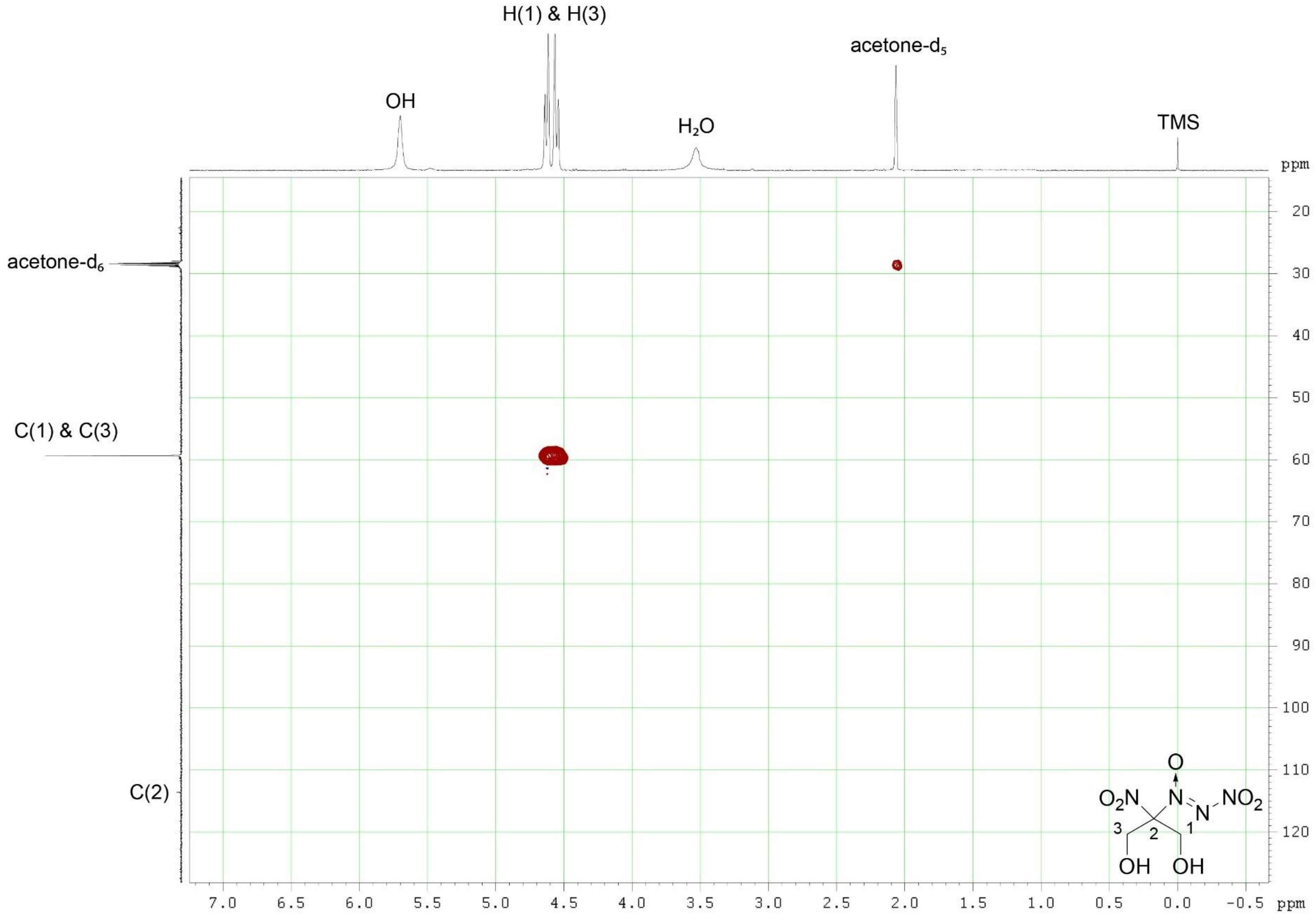

### 10.9.4 {$^1H$–$^{13}C$} HMBC spectrum of compound 3f [500.13 MHz, [D$_6$]acetone]

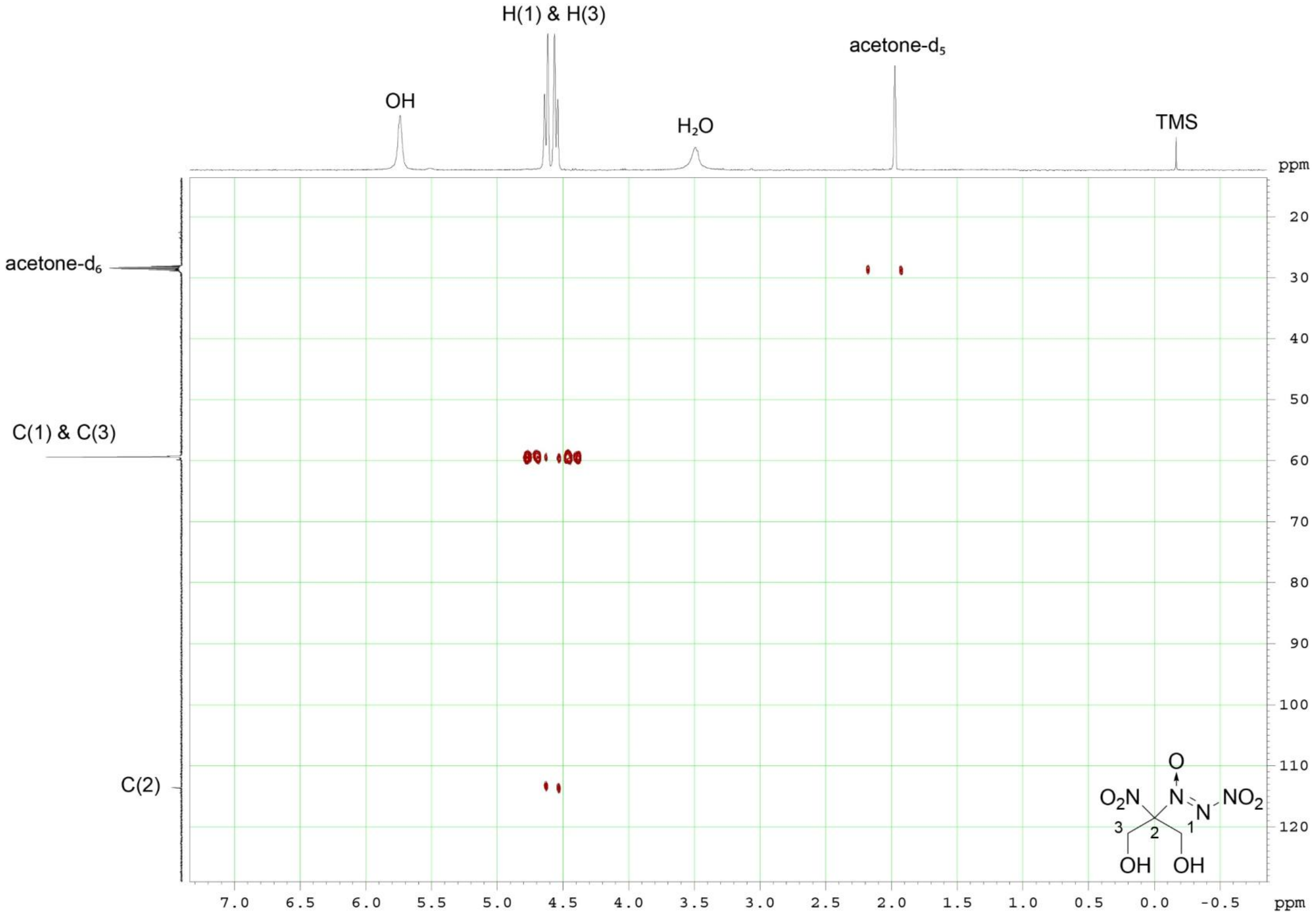

**10.9.5 $^{14}$N NMR spectrum of compound 3f [36.14 MHz, [D$_6$]acetone]**

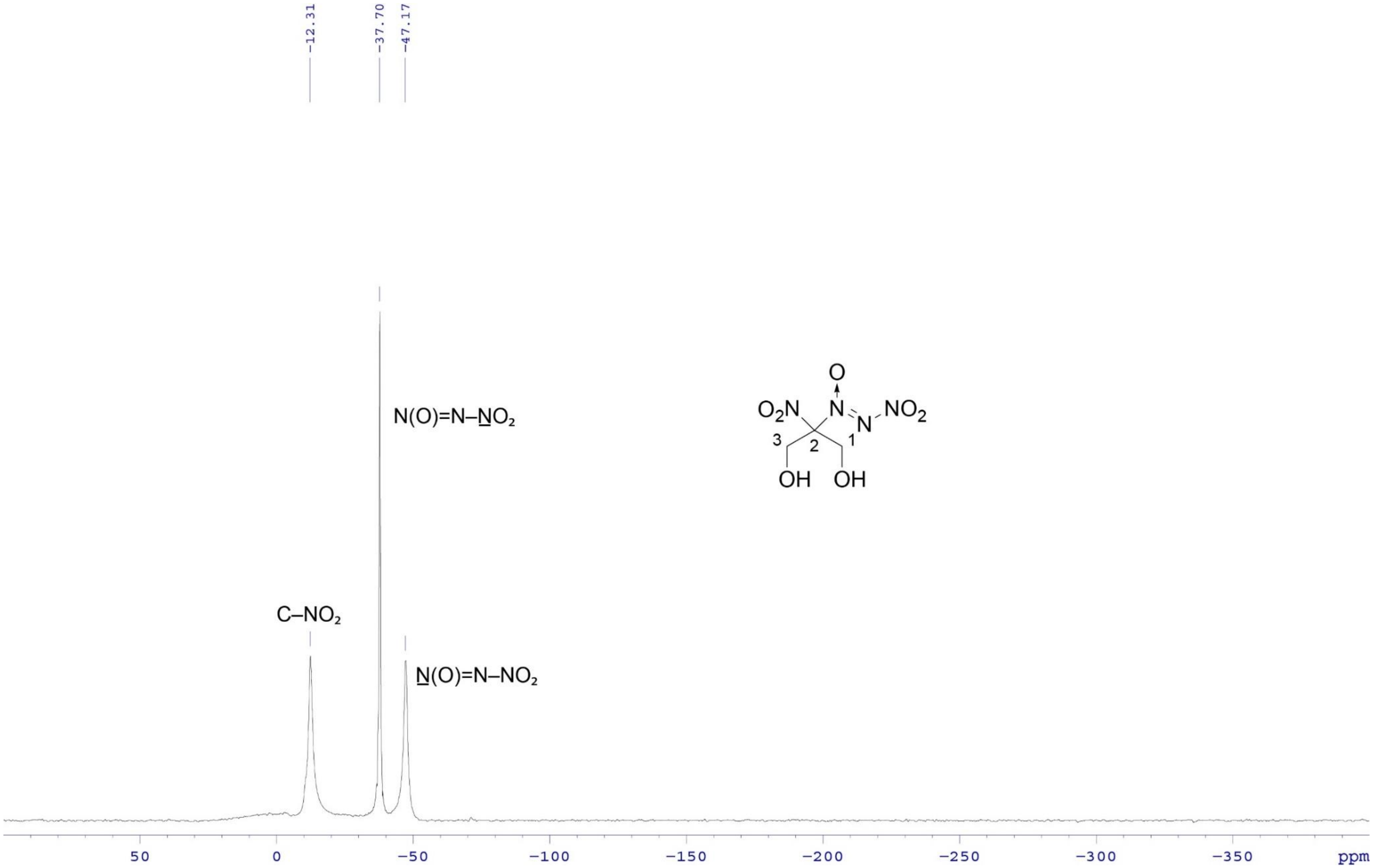

**10.10.1 $^{1}$H NMR spectrum of compound 4f [500.13 MHz, $CDCl_3$]**

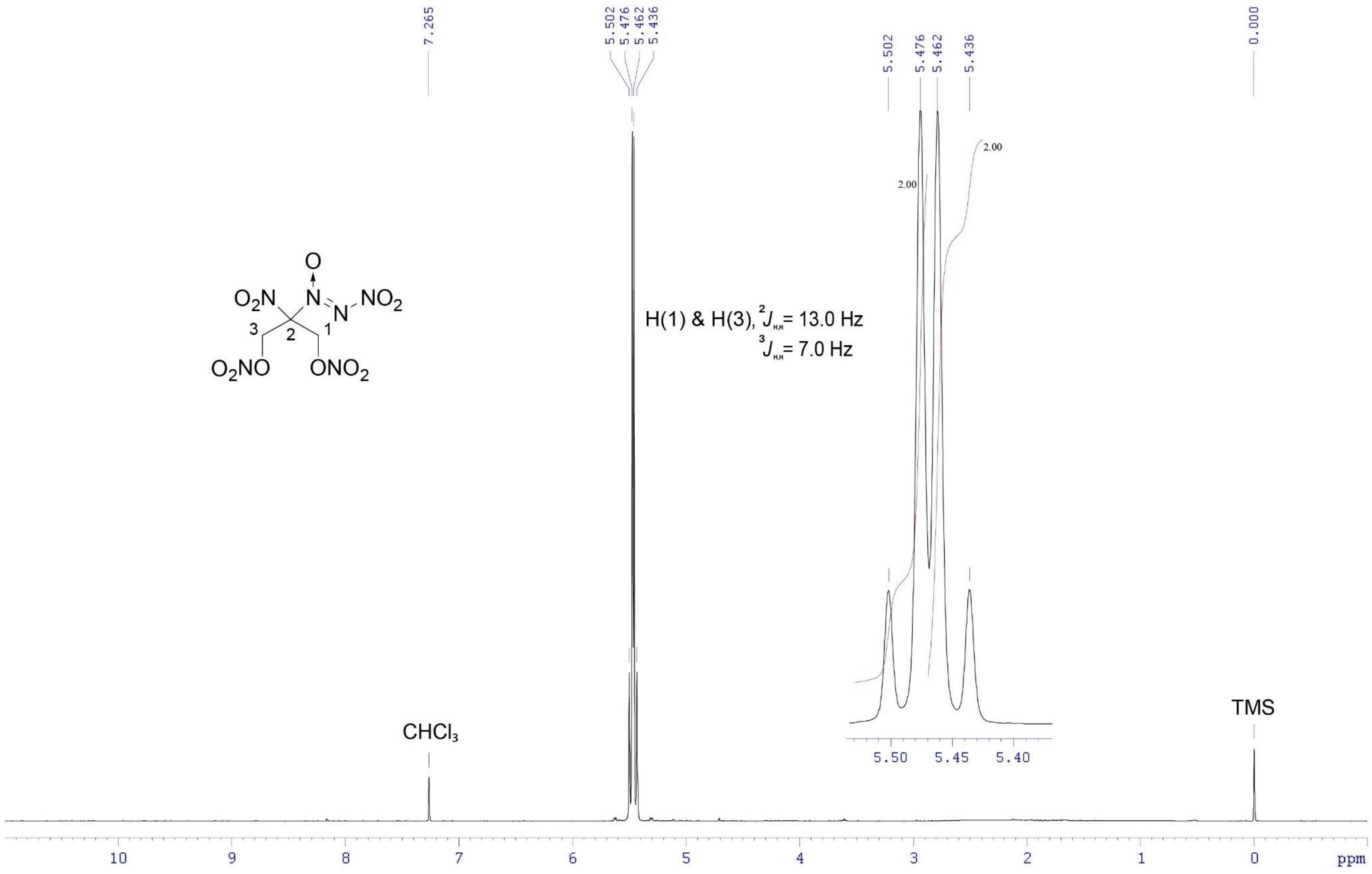

10.10.2 $^{13}C$ NMR spectrum of compound 4f [125.76 MHz, $CDCl_3$]

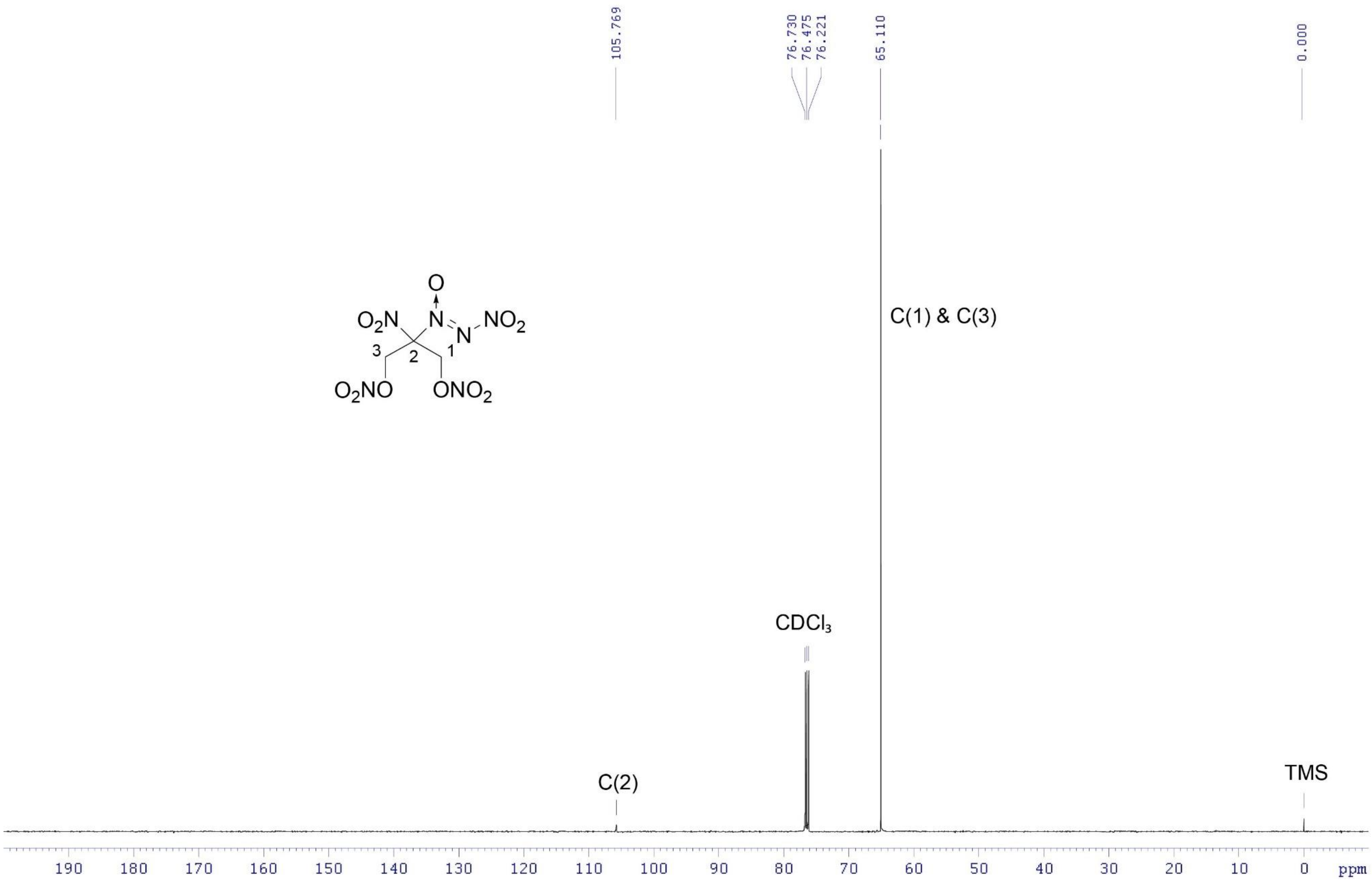

### 10.10.3 {$^{1}$H–$^{13}$C} HSQC spectrum of compound 4f [500.13 MHz, $CDCl_3$]

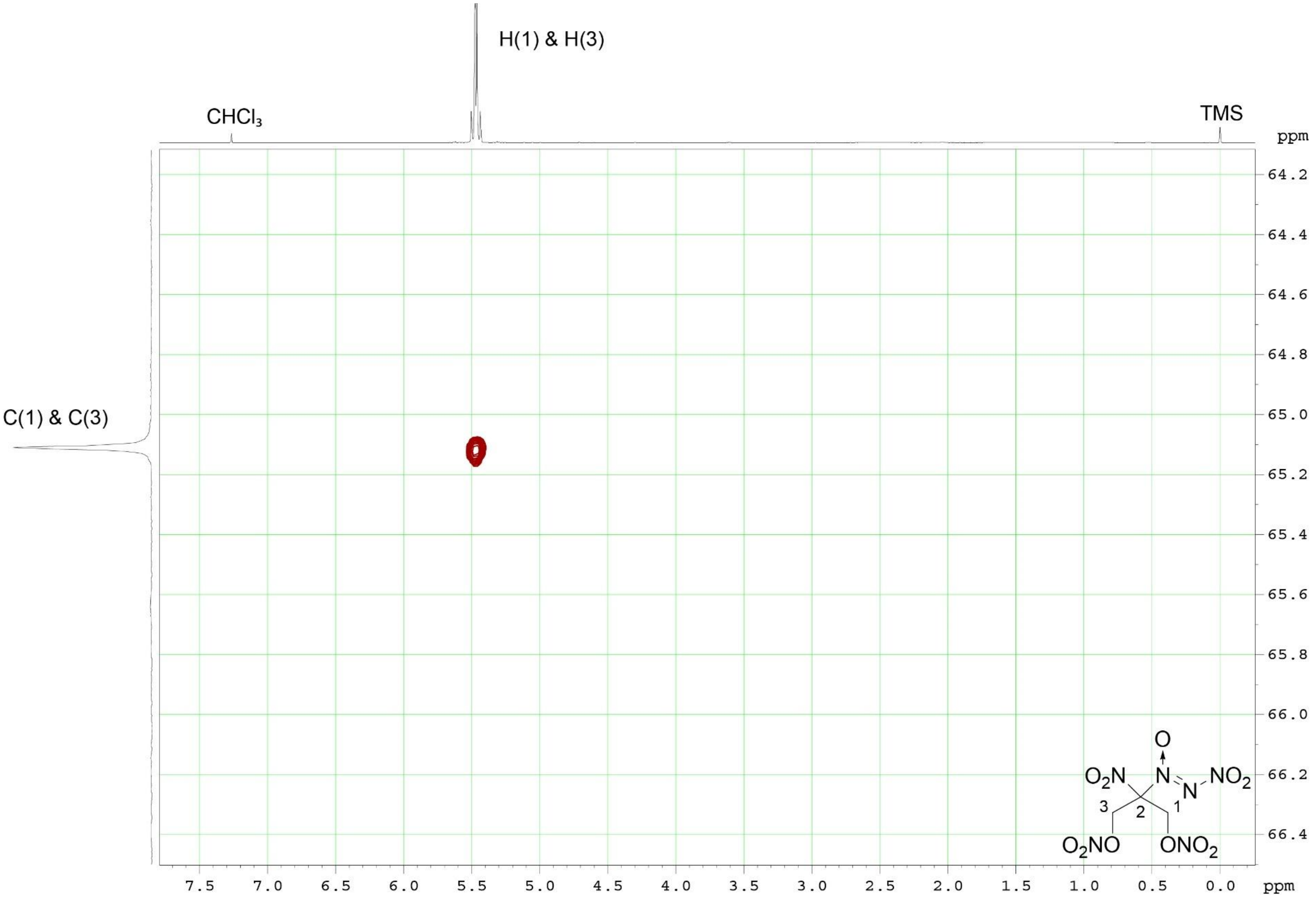

**10.10.4 {$^1$H–$^{13}$C} HMBC spectrum of compound 4f [500.13 MHz, $CDCl_3$]**

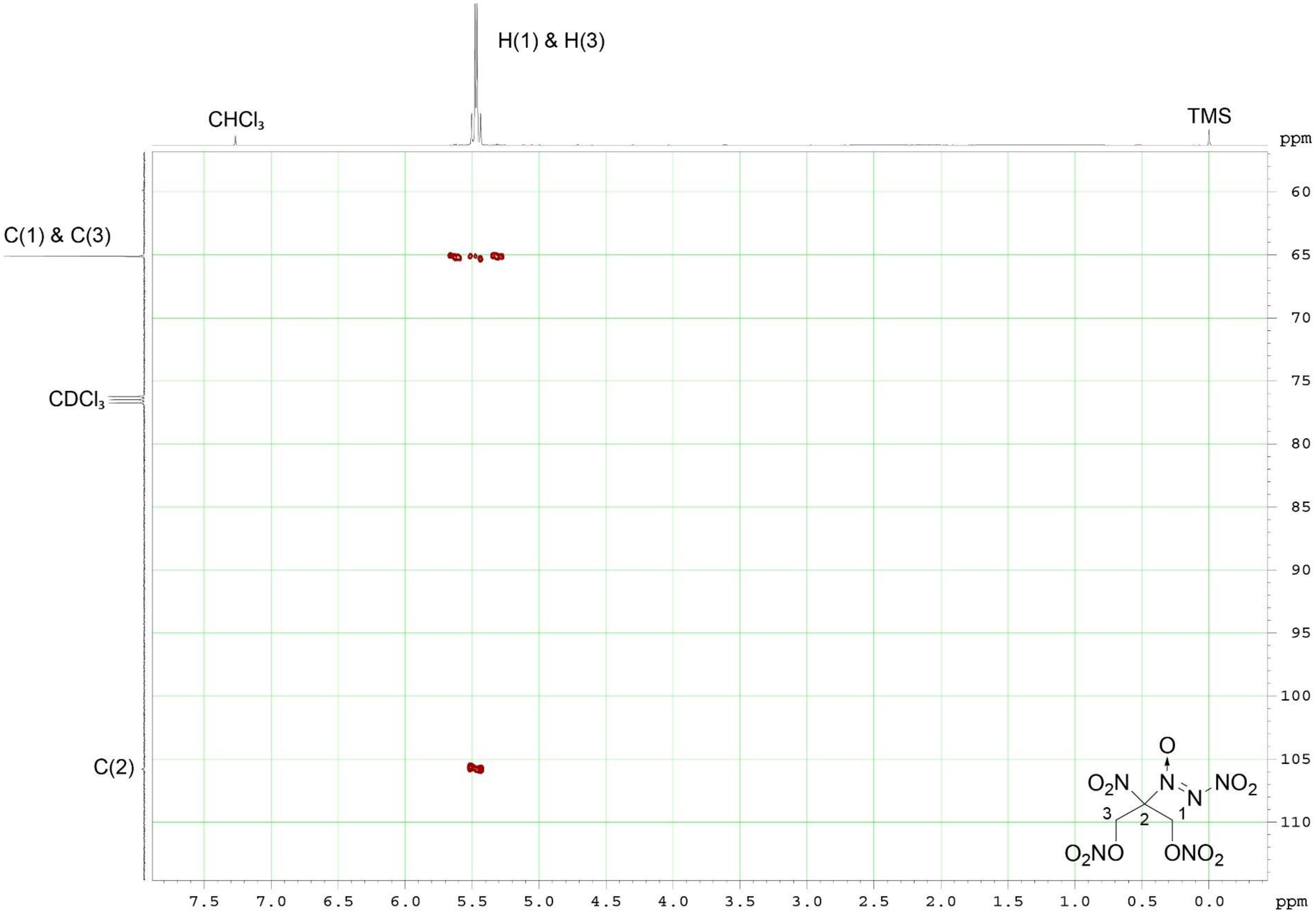

**10.10.5 $^{14}$N NMR spectrum of compound 4f [36.14 MHz, $CDCl_3$]**

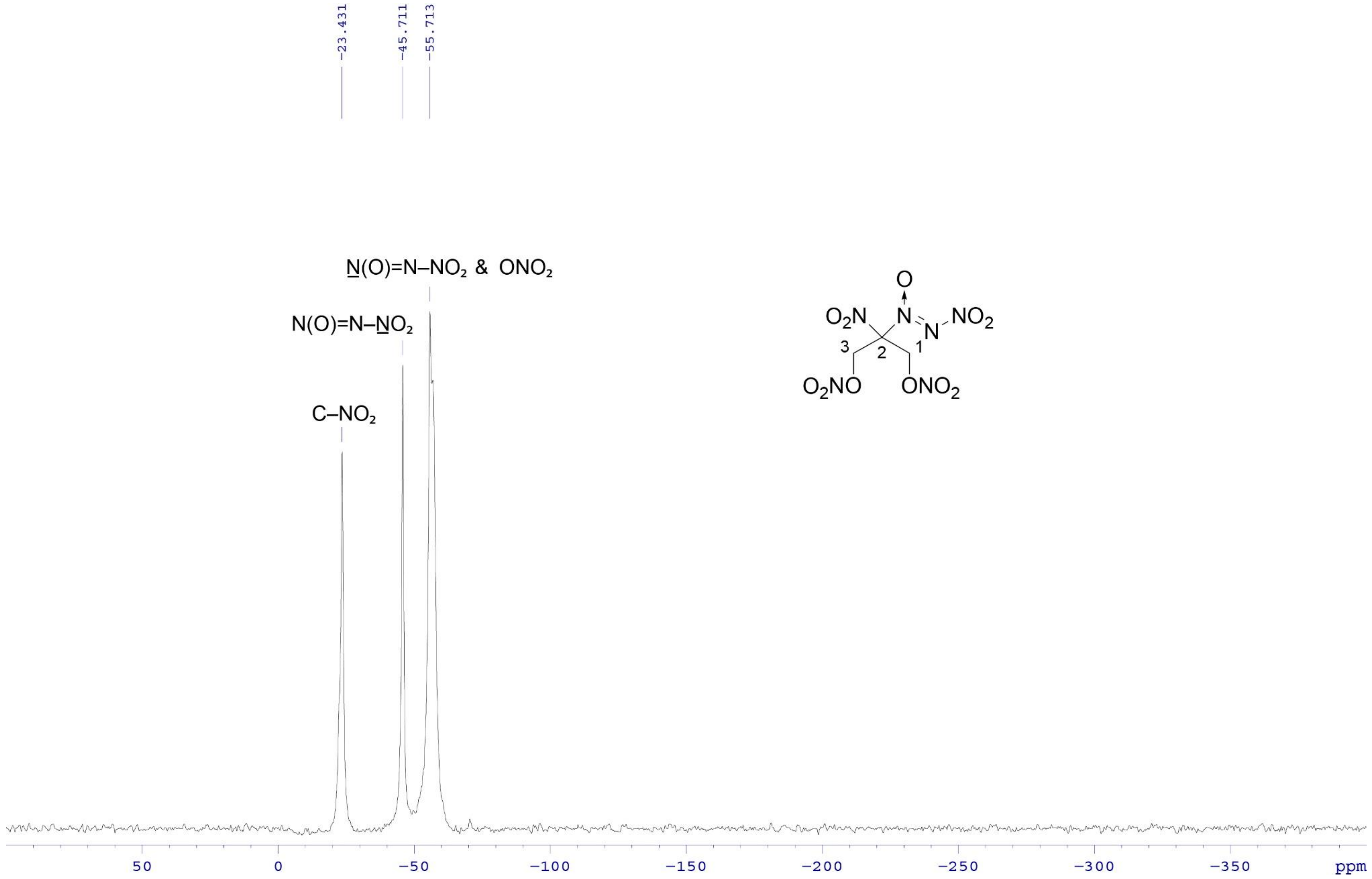

Supplement: File 1 — General information on materials and instruments, experimental procedures, and characterization data for all compounds, XRD of 2c, computational details, and copies of 1H, 13C, 14N, 1H,13C HSQC, and 1H,13C HMBC NMR spectra. [file Beilstein_J_Org_Chem-21-2739-s001.pdf]
