# Supplementary material for: Sustainable electrochemical synthesis of aliphatic nitro-NNO-azoxy compounds employing ammonium dinitramide and their in vitro evaluation as potential nitric oxide donors and fungicides
Source: Beilstein J Org Chem. 2025 Dec 29;21:2739–54. doi: 10.3762/bjoc.21.211 (PMC12766168; doi:10.3762/bjoc.21.211)

BEILSTEIN JOURNAL OF ORGANIC CHEMISTRY

Supporting Information

for

Sustainable electrochemical synthesis of aliphatic nitro-*NNO*-azoxy compounds employing ammonium dinitramide and their in vitro evaluation as potential nitric oxide donors and fungicides

Alexander S. Budnikov, Nikita E. Leonov, Michael S. Klenov, Andrey A. Kulikov, Igor B. Krylov, Timofey A. Kudryashev, Aleksandr M. Churakov, Alexander O. Terent'ev and Vladimir A. Tartakovsky

Beilstein J. Org. Chem. **2025,** *21,* 2739–2754. doi:10.3762/bjoc.21.211

CheckCIF report for the data of 2c

License and Terms: This is a supporting information file under the terms of the Creative Commons Attribution License (https://creativecommons.org/licenses/by/4.0). Please note that the reuse, redistribution and reproduction in particular requires that the author(s) and source are credited and that individual graphics may be subject to special legal provisions.
The license is subject to the *Beilstein Journal of Organic Chemistry* terms and conditions: (https://www.beilstein-journals.org/bjoc/terms)

checkCIF/PLATON report

Structure factors have been supplied for datablock(s) akaschz70

THIS REPORT IS FOR GUIDANCE ONLY. IF USED AS PART OF A REVIEW PROCEDURE FOR PUBLICATION, IT SHOULD NOT REPLACE THE EXPERTISE OF AN EXPERIENCED CRYSTALLOGRAPHIC REFEREE.

No syntax errors found. CIF dictionary Interpreting this report

Datablock: akaschz70

```
Bond precision:   C-C = 0.0018 A           Wavelength=1.54184

Cell:           a=8.5283(1)       b=10.5437(1)      c=10.9886(2)
                alpha=74.054(1)   beta=89.798(1)    gamma=86.786(1)
Temperature:    100 K

                 Calculated                   Reported
Volume           948.51(2)                    948.51(2)
Space group      P -1                         P -1
Hall group       -P 1                         -P 1
Moiety formula   C6 H10 N4 O5                 C6 H10 N4 O5
Sum formula      C6 H10 N4 O5                 C6 H10 N4 O5
Mr               218.18                       218.18
Dx,g cm-3        1.528                        1.528
Z                4                            4
Mu (mm-1)        1.163                        1.163
F000             456.0                        456.0
F000'            457.80
h,k,lmax         10,13,14                     10,13,14
Nref             4123                         4086
Tmin,Tmax        0.691,0.765                  0.232,1.000
Tmin'            0.551

Correction method= # Reported T Limits: Tmin=0.232 Tmax=1.000
AbsCorr = GAUSSIAN

Data completeness= 0.991           Theta(max)= 79.655

R(reflections)= 0.0387( 3898)                          wR2(reflections)=
                                                       0.0953( 4086)
S = 1.054                   Npar= 320
```

The following ALERTS were generated. Each ALERT has the format
test-name_ALERT_alert-type_alert-level.
Click on the hyperlinks for more details of the test.

Alert level B

```
PLAT430_ALERT_2_B Short Inter D...A Contact  O1        ..O3B      .       2.72 Ang.
                                              1-x,-y,2-z  =      2_657 Check
```

Author Response: This is due to the unique crystal structure.

```
PLAT430_ALERT_2_B Short Inter D...A Contact  N1        ..O3B      .       2.78 Ang.
                                              1-x,-y,2-z  =      2_657 Check
```

Author Response: This is due to the unique crystal structure.

Alert level C

```
PLAT906_ALERT_3_C Large K Value in the Analysis of Variance ......      2.074 Check
PLAT911_ALERT_3_C Missing FCF Refl Between Thmin & STh/L=    0.600          4 Report
                5 -9  3,   6 -8  3,    4 -9  4,    5 -8  4,
```

Alert level G

```
PLAT002_ALERT_2_G Number of Distance or Angle Restraints on AtSite         32 Note
PLAT003_ALERT_2_G Number of Uiso or U(i,j)  Restrained non-H-Atoms          2 Report
PLAT154_ALERT_1_G The s.u.'s on the Cell Angles are Equal ..(Note)      0.001 Degree
PLAT171_ALERT_4_G The CIF-Embedded .res File Contains EADP Records         15 Report
PLAT176_ALERT_4_G The CIF-Embedded .res File Contains SADI Records         17 Report
PLAT186_ALERT_4_G The CIF-Embedded .res File Contains ISOR Records          1 Report
PLAT191_ALERT_3_G A Non-default SADI Restraint Value has been used     0.0030 Report
PLAT191_ALERT_3_G A Non-default SADI Restraint Value has been used     0.0030 Report
PLAT191_ALERT_3_G A Non-default SADI Restraint Value has been used     0.0030 Report
PLAT191_ALERT_3_G A Non-default SADI Restraint Value has been used     0.0030 Report
PLAT191_ALERT_3_G A Non-default SADI Restraint Value has been used     0.0030 Report
PLAT191_ALERT_3_G A Non-default SADI Restraint Value has been used     0.0030 Report
PLAT191_ALERT_3_G A Non-default SADI Restraint Value has been used     0.0030 Report
PLAT191_ALERT_3_G A Non-default SADI Restraint Value has been used     0.0030 Report
PLAT191_ALERT_3_G A Non-default SADI Restraint Value has been used     0.0030 Report
PLAT191_ALERT_3_G A Non-default SADI Restraint Value has been used     0.0030 Report
PLAT191_ALERT_3_G A Non-default SADI Restraint Value has been used     0.0030 Report
PLAT191_ALERT_3_G A Non-default SADI Restraint Value has been used     0.0030 Report
PLAT191_ALERT_3_G A Non-default SADI Restraint Value has been used     0.0030 Report
PLAT191_ALERT_3_G A Non-default SADI Restraint Value has been used     0.0030 Report
PLAT191_ALERT_3_G A Non-default SADI Restraint Value has been used     0.0030 Report
PLAT191_ALERT_3_G A Non-default SADI Restraint Value has been used     0.0030 Report
PLAT191_ALERT_3_G A Non-default SADI Restraint Value has been used     0.0030 Report
PLAT301_ALERT_3_G Main Residue  Disorder .............(Resd    1)        60% Note
PLAT301_ALERT_3_G Main Residue  Disorder .............(Resd    2)        40% Note
PLAT367_ALERT_2_G Long?  C(sp?)-C(sp?) Bond  C7       - C8       .       1.51 Ang.
PLAT367_ALERT_2_G Long?  C(sp?)-C(sp?) Bond  C7       - C12      .       1.51 Ang.
PLAT811_ALERT_5_G No ADDSYM Analysis: Too Many Excluded Atoms ....          ! Info
```

```
PLAT860_ALERT_3_G Number of Least-Squares Restraints ............         29 Note
PLAT912_ALERT_4_G Missing # of FCF Reflections Above STh/L=  0.600        33 Note
PLAT969_ALERT_5_G The 'Henn et al.' R-Factor-gap value ..........      3.985 Note
              Predicted wR2: Based on SigI**2  2.39 or SHELX Weight  9.05
PLAT978_ALERT_2_G Number C-C Bonds with Positive Residual Density.        11 Info
```

```
   0 ALERT level A = Most likely a serious problem - resolve or explain
   2 ALERT level B = A potentially serious problem, consider carefully
   2 ALERT level C = Check. Ensure it is not caused by an omission or oversight
  32 ALERT level G = General information/check it is not something unexpected

   1 ALERT type 1 CIF construction/syntax error, inconsistent or missing data
   7 ALERT type 2 Indicator that the structure model may be wrong or deficient
  22 ALERT type 3 Indicator that the structure quality may be low
   4 ALERT type 4 Improvement, methodology, query or suggestion
   2 ALERT type 5 Informative message, check
```

It is advisable to attempt to resolve as many as possible of the alerts in all categories. Often the minor alerts point to easily fixed oversights, errors and omissions in your CIF or refinement strategy, so attention to these fine details can be worthwhile. In order to resolve some of the more serious problems it may be necessary to carry out additional measurements or structure refinements. However, the purpose of your study may justify the reported deviations and the more serious of these should normally be commented upon in the discussion or experimental section of a paper or in the "special_details" fields of the CIF. checkCIF was carefully designed to identify outliers and unusual parameters, but every test has its limitations and alerts that are not important in a particular case may appear. Conversely, the absence of alerts does not guarantee there are no aspects of the results needing attention. It is up to the individual to critically assess their own results and, if necessary, seek expert advice.

Publication of your CIF in IUCr journals

A basic structural check has been run on your CIF. These basic checks will be run on all CIFs submitted for publication in IUCr journals (*Acta Crystallographica*, *Journal of Applied Crystallography*, *Journal of Synchrotron Radiation*); however, if you intend to submit to *Acta Crystallographica Section C* or *E* or *IUCrData*, you should make sure that full publication checks are run on the final version of your CIF prior to submission.

Publication of your CIF in other journals

Please refer to the *Notes for Authors* of the relevant journal for any special instructions relating to CIF submission.

PLATON version of 02/02/2025; check.def file version of 02/02/2025

Datablock akaschz70 - ellipsoid plot

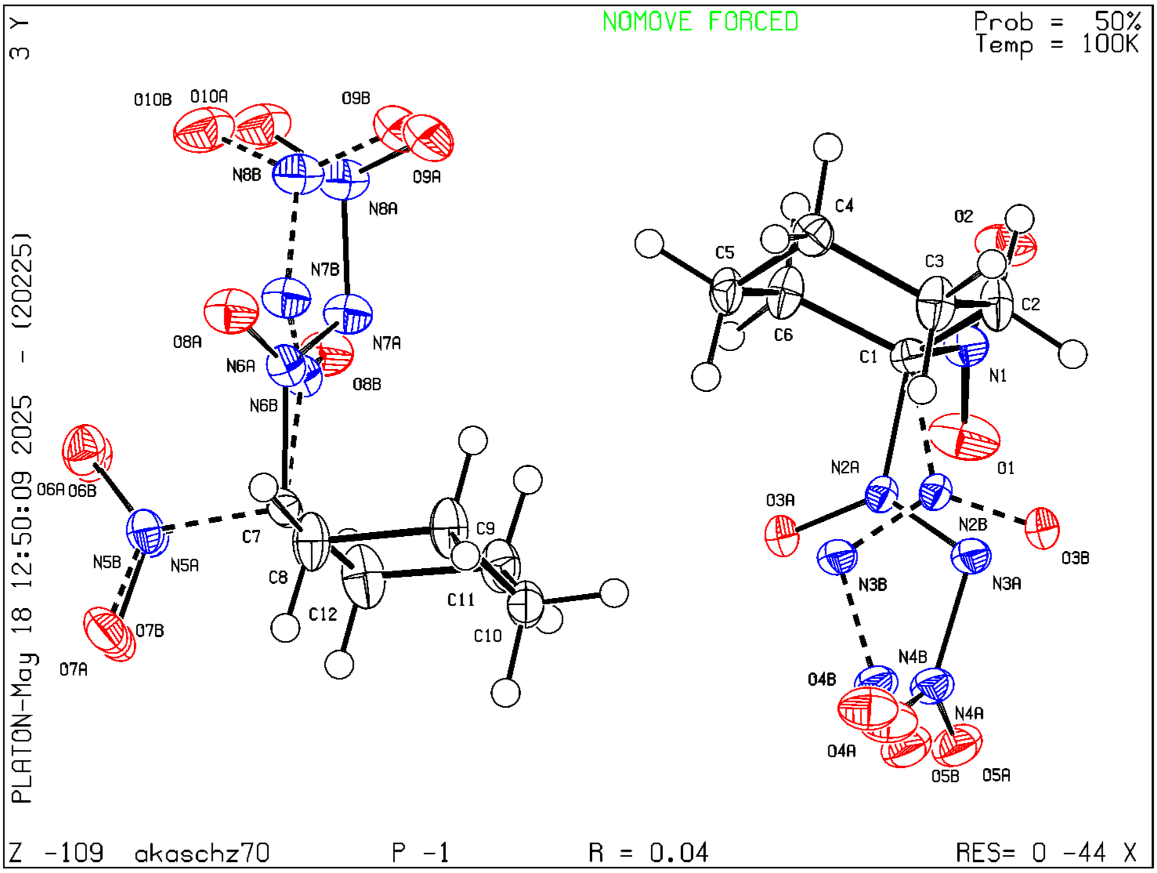

Supplement: File 3 — CheckCIF report for the data of 2c. [file Beilstein_J_Org_Chem-21-2739-s003.pdf]
